# SARS-CoV-2 accelerated clearance using a novel nitric oxide nasal spray (NONS) treatment: A randomized trial

**DOI:** 10.1016/j.lansea.2022.100036

**Published:** 2022-06-29

**Authors:** Monika Tandon, Wen Wu, Keith Moore, Stephen Winchester, Yuan-Po Tu, Christopher Miller, Rahul Kodgule, Amol Pendse, Shabbir Rangwala, Shashank Joshi, Study Group

**Affiliations:** a Glenmark Pharmaceuticals Limited, Mumbai, India; b SaNOtize Research & Development Corp., Vancouver, British Columbia, Canada; c Frimley Health NHS Foundation Trust, Surrey, England; d The Everett Clinic-Part of Optum, Everett, Washington; e Glenmark Pharmaceuticals Limited, UK; f Lilavati Hospital and Research Centre, Mumbai, India

**Keywords:** COVID-19, Anti-viral treatment, Nasal Spray, SARS-CoV-2, Viral load

## Abstract

**Background:**

Additional outpatient therapies which are readily accessible will be essential to reduce COVID-19 illness progression in high risk individuals. Especially as the virus continues to mutate with greater transmissibility despite increased global vaccination.

**Methods:**

A randomized, double-blind, multicentre, parallel group, placebo-controlled phase III clinical trial evaluated the ability of nitric oxide (NO) to rapidly eradicate nasal SARS-CoV-2 RNA. Adults (18–70 years) with mild symptomatic COVID-19 were randomized, confirmed by laboratory SARS-CoV-2 reverse transcription polymerase chain reaction (RT-PCR) nasal swab. Randomisation was 1:1, NONS (*N* = 153) vs placebo (*N* = 153). NO generated by a nasal spray (NONS) was self-administered six times daily as two sprays per nostril (0⋅45 mL of solution/dose) for seven days. Patients at high risk of illness progression, defined as unvaccinated, ≥ 45 years of age or having comorbidities, were the primary analysis population.

**Findings:**

Overall, mean SARS-CoV-2 RNA concentrations (6·96 log10 copies/mL in the NONS group and 7·16 log10 copies/mL in the placebo group) were comparable at baseline. Primary endpoint mean treatment difference SARS-CoV-2 RNA change from baseline to the end of treatment (EOT) was -0·52 copies/mL (SE 0·202, 95% CI -0·92 to -0·12; *p* = 0·010) with NONS compared to placebo. Secondary endpoint assessments demonstrated a greater proportion of patients receiving NONS (82·8%) cleared SARS-CoV-2 (RT-PCR negative) by EOT compared to placebo (66·7%, *p* = 0·046), with no virus RNA detected a median of four days earlier compared to placebo (three vs seven days; *p* = 0·044).

**Interpretation:**

Use of NONS in patients recently infected with SARS-CoV-2 accelerates nasal virus clearance.

**Funding:**

Funding provided by Glenmark Pharmaceuticals Limited. Study medication provided by SaNOtize.

**Research in context**

**Evidence before this study**

Prior to this study, evidence from several sources, including book citations, journal publications and SaNOtize's laboratory investigations have recognized the antiviral effect of nitric oxide. Minimal clinical research exploring the impact of intranasal NO administration on COVID-19 infections from SARS-CoV-2 has been uncovered, except for a comparable Phase II trial conducted in the UK (January to February 2021).

**Added value of this study**

Nitric oxide nasal spray (NONS) administrated six times daily for seven days was efficacious in accelerating the reduction of SARS-CoV-2 RNA from the nasal cavity. This has implications for treatment of recent or established COVID-19 infection to reduce the transmission of virions using a novel treatment and delivery system.

**Implications of all the available evidence**

Evidence uncovered supports the hypothesis of using intranasal NO to accelerate the reduction of SARS-CoV-2 from the nasal cavity. Implications include decreasing the duration of COVID-19 infectivity, possibly reducing hospital admissions, diminishing disease severity and disease transmission. The findings from this study can be used as supporting evidence for the use of NONS for patients with recent infections to reduce their risk of illness progression.

Alt-text: Unlabelled box

## Introduction

COVID-19 remains an ongoing global concern.^1^ A newly developed nitric oxide (NO) releasing solution accelerated the clearance of nasal SARS-CoV-2 RNA in a recently conducted randomized clinical Phase II trial.^2^ The outpatient therapy has been further improved as a small volume (0⋅45 mL/dose) nitric oxide nasal spray (NONS) self-administered into each nostril using a 25 mL spray bottle. NONS targets the virus within the nasal cavity at the point of initial contact before nasal host cell entry and post-replication release from host cells.

Laboratory investigations have demonstrated an immediate virucidal action of NO against all VOCs of SARS-CoV-2 and other respiratory pathogens (unrelated to the other ingredients in the product).^3^ Mechanistically, NO immediately alters the structural integrity of viral proteins through nitrosylation and palmitoylation reduction; interferes with the fusion of spike (S) protein to its cognate host receptor angiotensin converting enzyme 2 (ACE-2); and impedes viral protease activity resulting in the inhibition of viral RNA replication.^4^, ^5^, ^6^, ^7^ The inherent low pH (3⋅5) and virion trapping capacity (hypromellose) of NONS augments the antiviral activity of NO.

Here we present the results of a Phase III randomised double-blind placebo-controlled trial in non-hospitalized patients with mild COVID-19 infection. The primary outcome measure of nasal SARS-CoV-2 RNA accelerated clearance was used to assess the efficacy of this transformational nitric oxide nasal spray in high risk patients (unvaccinated, ≥ 45 years of age, or had one or more comorbidities) after 7 days of treatment. Secondary outcomes were to achieve a greater proportion of high risk patients rapidly cleared of viral RNA, with the intent to improve their clinical status and minimize COVID-19 illness progression.

## Methods

### Study design

In this multicentre, double-blind, parallel-group efficacy and safety study, symptomatic adults within three days of symptoms onset with a positive rapid SARS-CoV-2 antigen test were randomized to receive NONS or placebo. Nasal SARS-CoV-2 RNA presence was confirmed by a positive reverse transcription polymerase chain reaction (RT-PCR) screening swab test (results were not available until post-randomization).

Subjects self-administered nitric oxide nasal spray or placebo two sprays per nostril; 0·45 mL/dose six times a day, at least two to three hours apart while awake for seven treatment days (Day one to Day eight [end of treatment (EOT)]). All participants received standard supportive care in accordance with latest guidelines issued by Ministry of Health and Family Welfare from the Government of India, which included antipyretics for the treatment of fever and pain, antitussives for cough, and adequate hydration/nutrition.

The protocol received approval from each Investigator's site Institutional Ethics Committee (IEC). The trial was carried out in accordance with the Good Clinical Practice (GCP), principles of the Declaration of Helsinki, and all applicable national and local regulatory requirements. The trial was initiated with 20 clinical sites across India; 15 sites enrolled subjects in the study from 10 August 2021 to 25 January 2022. A low incidence of COVID-19 infections and other priority studies prevented recruitment in five sites. A summary of the key protocol amendments based on discussion with the regulatory authority Drug Controller General of India (DCGI) is described in the Table S1 (Supplementary Material; Appendix).

### Participants

Eligible adult men and women (aged 18–70 years) with mild COVID-19 symptoms and a positive nasal rapid antigen test for SARS-CoV-2 were enrolled into the study regardless of their vaccination status. Mild symptoms of COVID-19 included fever, cough, sore throat, malaise, headache, nasal congestion, muscle pain, gastrointestinal symptoms, lack of taste or smell without shortness of breath or dyspnea. The maximum permitted difference in the time of onset of symptoms and the time of treatment was ≤ 72 hours. Blood oxygen saturation (SpO2) > 94 % and respiratory rate < 24 breaths/min were also enrolment criterion.

For women to be enrolled, evidence of post-menopause, or a negative pre-treatment urine pregnancy test (for pre-menopausal subjects) was required. Women of child-bearing age (female or male with female partner of child-bearing age) agreed to take effective contraceptive measures (including hormonal contraception, barrier methods or abstinence) with his/her partner during the study period and for at least seven days following the EOT. All exclusion criteria and prohibited medications are listed in Table S2 (Appendix).

Participants had to be capable of providing informed consent, self-administering the nasal spray, and recording clinical signs indicative of COVID-19 symptoms as defined by FDA COVID-19 Guidance Document.^8^ Written consent was obtained from all participants.

### Randomisation and masking

Three hundred and six eligible participants were planned to be randomised in a 1:1 ratio to receive NONS or matching identical placebo nasal spray using a computer-generated randomisation scheme (Table S3, Appendix). Blinding of the treatment allocation was achieved by using treatment kits identified by a numeric code; treatment allocation was based on kit numbers. A data safety monitoring board reviewed the trial and study data.

### Procedures

A detailed clinical history, physical examination, vaccination status, vital signs, SpO2 (measured non-invasively by Rad-57 Signal Extraction Pulse CO-Oximeter; Masimo International, US), 12-lead ECG, chest x-ray, blood (chemistries and haematology)/urine collections for laboratory evaluation and eligibility assessment were performed after obtaining informed consent (Table S2, Appendix). Rapid Antigen Test (Meril Diagnostics Pvt. Ltd, sensitivity 96·6%, specificity 100%) for COVID-19 was conducted as part of the screening assessment by trained site personnel. Subjects with a negative COVID-19 antigen result were considered a screen failure. Subjects with a positive COVID-19 antigen result were randomized. Swabs from both nostrils were also taken for quantitative and qualitative RT-PCR assessments at baseline prior to therapy by trained site personnel. The quantitative virus RNA was assessed at a central laboratory (Metropolis, Mumbai). Assay data are presented in units of SARS-CoV-2 genome equivalent copies per mL log10 values. A complete description of the assay method is provided in Table S2 (Appendix).

Participants were randomized into one of the two treatment groups, supplied a blinded study medication 25 mL nasal spray bottle, and instructed on how to self-administer each dose. The NONS and placebo spray bottles were identical in appearance (shape and size [round], colour [white], with a 22mm tapered white nasal tip actuator covered by a clear dust cap), smell and taste. NONS contained two NO generating ingredients, gelling agent (hypromellose, [HPMC]), preservative (benzalkonium chloride 0·01%) in normal saline (0·9% sodium chloride). The placebo contained preservative (benzalkonium chloride 0·01%) in normal saline. *In vitro* antiviral activity of NONS and placebo are described in Table S4 (Appendix). Standard supportive care was provided to all subjects during the study.

Subject diaries were dispensed at the baseline visit (Day one). Subjects recorded their health status and COVID-19 related symptoms daily during study participation; including adverse events (AEs), use of concomitant medications and study drug compliance. Investigators recorded the participants’ clinical status using the World Health Organization (WHO) Clinical Progression Scale (CPS) Score at baseline. The WHO CPS is based on an 11-point scale ranging from a score of 0 (asymptomatic/no viral RNA detected) to 10 (death).^9^ COVID-19 related symptoms scores were obtained using the patient reported outcomes (PRO) US-FDA symptoms questionnaire format. Methemoglobin (MetHb; Pulse CO-Oximeter) was measured in a subgroup of subjects.

On Day two (≤ 24 hours on treatment), Day four (72 h on treatment), and Day eight (seven days on treatment; EOT) study site procedures included AE review, concomitant medication review, study medication compliance review, nasal swabs for RT-PCR, SpO2, MetHb, vital signs and investigator's WHO CPS score; Day three (48 h on treatment) nasal swab for RT-PCR; Day four included a chest x-ray or CT scan (investigator's discretion); Day eight also included a physical examination, 12-lead ECG and blood/urine laboratory evaluations.

Any subjects having a negative baseline qualitative RT-PCR swab result were withdrawn from the study once the result was available (within one day post randomisation). For subjects having a negative qualitative RT-PCR (RT-PCR negative) result at Day four, no RT-PCR assessments were done on Day eight.

The last subject contact was on Day 19 ± two days (with standard of care continued), or any time between Day eight and Day 19 if the subject's COVID-19 symptom status and RT-PCR result became negative. The contact was either a telephonic visit or site visit. Concomitant medications, AEs and WHO CPS scores were recorded. For subjects who were RT-PCR positive on Day eight, a nasal swab from both nostrils was collected every two to three days until their qualitative RT-PCR assessment became negative or study end reached. Subjects were asked on Day one (baseline), Day two, Day four, Day 8 (EOT), and their last day about the total number of immediate contacts (at least eight hours of close daily contact) that had become infected with COVID-19 (test confirmed). The total number of immediate contacts with symptoms suggestive of COVID-19 infection was captured.

### Efficacy outcomes

The primary objective and endpoint were set to demonstrate the ability of NONS to reduce SARS-CoV-2 RNA rapidly and significantly, using the change from baseline in log viral RNA through EOT measured by quantitative RT-PCR. The secondary objectives and endpoints were focused on clinical and viricidal improvement support measures, including the proportion of subjects with negative conversion of SARS-CoV 2 RT-PCR on Day two, three, four, and eight; time required to achieve a RT-PCR negative status; proportion of patients achieving a two point change in WHO CPS Score on Day two, four, eight and 19; proportion of subjects requiring oxygen use or hospitalization for the treatment of COVID-19 by Day 19 and change from baseline in COVID-19 related symptoms at Day two, three, four, eight and 19.

### Safety outcomes

Safety and tolerability of NO nasal spray treatment (secondary endpoints) included the number and types of AEs and serious adverse events (SAEs). An AE was defined as any unfavourable and unintended sign (including an abnormal laboratory finding), symptom or disease (new or exacerbated) associated with the use of the study drug, whether or not related to the study drug. An SAE included any untoward medical occurrence that resulted in a death; was life-threatening; required a hospitalization or prolongation of an existing hospitalization; resulted in disability/incapacity or was an important medical event that may have jeopardized the subject or required medical/surgical intervention to prevent one of the other outcomes previously listed.

Change from baseline in vital signs, ECG parameters, SpO2, MetHb, laboratory (chemistry, hematology, and urine) valuations and physical examinations at EOT and/or the study end (Day 19) were also determined.

### Statistical analysis

Primary and secondary efficacy analyses were conducted on data from subjects who had been randomized, received at least one dose of study medication, had non-missing baseline measurements (excluded 58 subjects with negative RT-PCR at screening and 35 with negative RT-PCR up to one day post-randomisation) and at least one post-baseline efficacy measurement for the primary efficacy variable (modified intent-to-treat [mITT] population). The regulatory authority, DCGI, recommended analysis be focused on those at risk of illness progression, i.e., high risk population. The high risk population was defined as subjects that were unvaccinated, age ≥ 45 years or who had comorbidities. Analyses was also conducted in the unvaccinated and mITT populations. The per protocol (PP) population included all subjects who were randomized, received at least one dose of study medication, completed the study, and did not have any major protocol deviations. The high risk PP population analysis conducted was supportive. Major protocol deviations were discussed and decided at the blinded data review meeting before the database lock.

The primary efficacy endpoint used the quantitative change from baseline in SARS-CoV-2 RNA (log10 copies per mL) over seven days of therapy and was analysed by both an average (least square mean [SE]) and normalised AUC method. The AUC analysis of viral RNA utilized the time-weighted average change from baseline to each visit calculated for each subject as the area under the log10 concentration–time curve, with the use of the linear trapezoidal rule for change from baseline (CFB) divided by the time interval of the observation period.^10^

The comparisons of nitric oxide versus placebo were analysed using the mixed model repeated measures (MMRM) method. The MMRM model included data from all visits until EOT with the following covariates: treatment, visit, baseline value (log10 copies per mL), risk factor (high risk yes/no), centre, and treatment by visit interaction; unstructured covariance matrix were used thus allowing adjustment for correlations between the time points within subjects.^11^

The secondary efficacy analysis included the proportion of subjects with negative conversion of SARS-CoV 2 RT PCR on Day two, four or eight based on qualitative RT-PCR positive/negative results. The time to event, a negative SARS-CoV2 RT-PCR (conversion) result, was analysed using the Kaplan-Meier method and log-rank. The proportion of patients achieving a 2-point change in WHO CPS score on Day two, four, eight and through 19 (study end), and proportion of subjects requiring hospitalization or oxygen use were analysed using the chi-square test or Fisher's exact test. An exploratory evaluation was conducted on the proportion of the immediate study subjects’ contacts becoming infected with COVID-19 (i.e., % of contacts per evaluation Day/treatment group for symptoms or SARS-CoV-2 positive test).

Safety analyses were conducted on all randomized subjects who had received at least one dose of study drug (safety population).

The statistical and analytical plans for this study are described in the protocol and statistical analysis plan (SAP). All analyses were performed using SAS® software V 9·4. Datasets were prepared using headings from Clinical Data Interchange Standard Consortium (CDISC) Analysis Data Model (ADaM). The SAP was prepared prior to final study data analysis. Any modification to the analysis plan was updated in the SAP. In general, all data were summarized with descriptive statistics (number of subjects, mean, and standard deviation, minimum, median and maximum) for continuous endpoints, and frequency and percentage for categorical endpoints. Any other summary statistics are described on an individual basis.

### Sample size determination

Sample size calculations were based on estimated AUC data (Day one to six) from the Phase II Nitric Oxide Nasal Spray treatment trial conducted in the United Kingdom (Clinical Study Report IRAS ID 287727 NONS COVID Study).^2^ The AUC estimate was the best available data, albeit applied to a slightly longer SARS-CoV-2 assessment interval used in this study (end of treatment). As AUC is not easily interpretable^8^ and considered proportional to the mean change in viral RNA from baseline, the primary endpoint focused on the mean change from baseline analysis. The current trial assumed a standardised effect size of 0⋅5; 128 to 172 evaluable subjects (64 to 86 subjects per arm) would provide a power of 80% to 90% with two-sided significance level of 5%. For the secondary endpoint (RT-PCR conversion), assuming an RT-PCR conversion of 40% in the placebo group by EOT, 194 to 260 subjects (97 to 130 subjects in each arm) would provide a power of 80 to 90% at two-sided significance level of 5%, to detect a treatment difference of 20% in proportion of subjects achieving RT-PCR conversion. Assuming a dropout rate of 15%, 306 total subjects (153 subjects per arm) was the enrollment target.

Subjects using ≤ 80% of their total expected doses of study medication over the treatment period were considered non-compliant. The trial is registered with Clinical Trials Registry India with identifier: CTRI/2021/08/035432.

**Role of the funding source:** Glenmark Pharmaceuticals Limited is the sponsor of the study.

## Results

**Figure 1:**
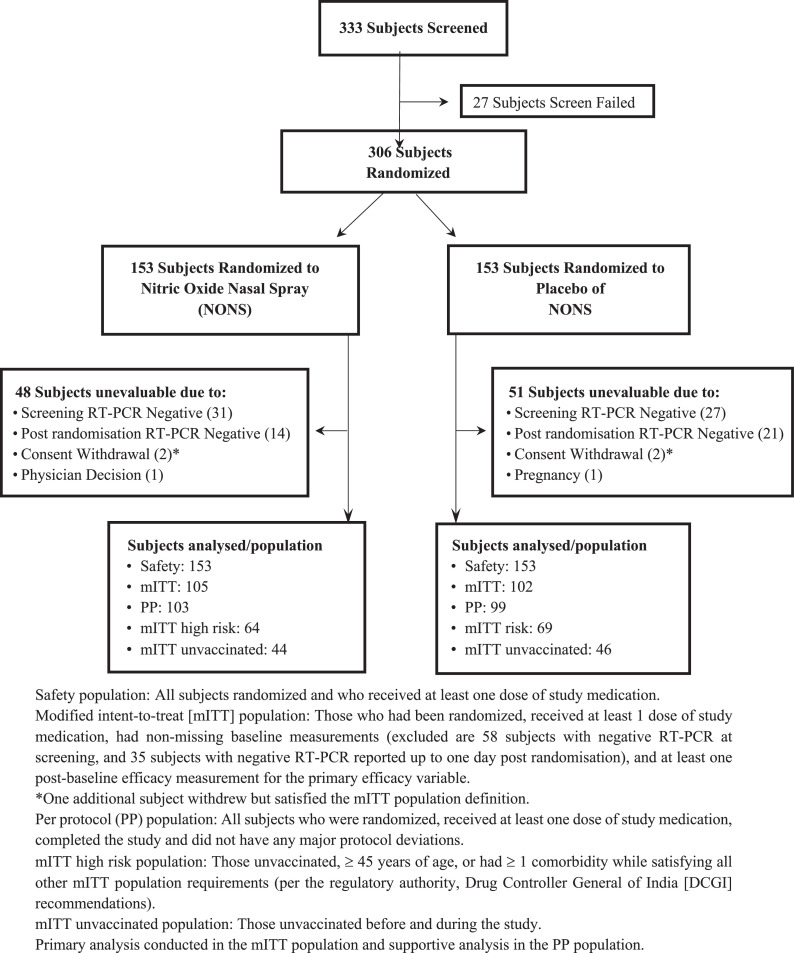
**NONS Study Trial Disposition**.

A total of 333 adults were screened, 58% during the second (Delta predominant) VOC wave and 42% during the third (Omicron/Delta predominant) VOC wave (27 screen failures not randomized). Of the 306 subjects randomized, 153 subjects were randomized to the NONS group, and 153 subjects were randomized to placebo group (Figure 1). Sixty-six (21·6%) subjects were withdrawn from the study after randomization; 58 (19·0%) due to a negative RT-PCR screening result reported post-randomization (subjects followed through their quarantined days). Of the 306 randomized subjects, all 306 (100·0%) subjects were included in safety analysis population.

The number of subjects completing the trial was comparable between the NONS and placebo groups, 118 subjects/NONS and 122 subjects/Placebo, respectively. Eight subjects did not complete the study. The most common reason for discontinuation was withdrawal by the subject [six (2·0%), followed by one (0·3%) each due to physician's decision and a pregnancy]. The mITT population analyses included 207 subjects (*N =* 105 in NONS; *N =* 102 in placebo, with all subjects having a positive RT-PCR value at screening and up to one day post randomization (35 additional subjects excluded from the primary analyses for a negative RT-PCR), high risk mITT population analyses included 133 subjects (*N =* 64 analysed in NONS; *N =* 69 in placebo). The unvaccinated mITT population analyses included 90 subjects (*N =* 44 analysed in NONS; *N =*46 in placebo).

Patients started NONS or placebo within 3 days of symptoms onset. Patients had mild symptoms at the time of randomisation. Both groups had comparable baseline and demographic characteristics (Safety population; Table 1). Baseline and demographic characteristics for the high risk mITT population, unvaccinated mITT population and mITT population are listed in Table S5 (Appendix). In the NONS group, 39⋅2% had received two doses of COVID-19 vaccine; 37⋅3% in the placebo group. Greater than 99% of subjects in either group were treatment drug compliant.

**Table 1:** Characteristics of the patients at baseline (safety population).

Parameter	Statistics	NONS (*N =* 153)	PLACEBO (*N =* 153)	Total (*N =* 306)
Age (Year)	N	153	153	306
	Mean	37·4	38·1	37·8
	SD	12·63	11·63	12·13
	Median	35·0	36·7	36·4
	Range	18–69	19–68	18–69
Weight (Kg)	N	153	153	306
	Mean	63·87	63·26	63·57
	SD	9·494	9·215	9·345
	Median	64·60	62·40	63·45
	Range	37·0–94·0	40·0–94·0	37·0–94·0
Age subgroup (Year)				
< 45	n (%)	118 (77·1)	106 (69·3)	224 (73·2)
≥ 45	n (%)	35 (22·9)	47 (30·7)	82 (26·8)
Sex				
Female	n (%)	51 (33·3)	58 (37·9)	109 (35·6)
Male	n (%)	102 (66·7)	95 (62·1)	197 (64·4)
Ct value at baseline	n (%)	122 (79·7)	125 (81·7)	247 (80·7)
Median^a^		23	23	23
< 30	n (%)	107 (69·9)	115 (75·2)	222 (72·5)
≥ 30	n (%)	15 (9·8)	10 (6·5)	25 (8·2)
High Risk^b^				
No	n (%)	45 (29·4)	43 (28·1)	88 (28·8)
Yes	n (%)	108 (70·6)	110 (71·9)	218 (71·2)
Co-morbidities				
No	n (%)	132 (86·3)	137 (89·5)	269 (87·9)
Yes (any co-morbidities^c^)	n (%)	21 (13·7)	16 (10·5)	37 (12·1)
Vaccination Status				
Unvaccinated Subjects	n (%)	83 (54·2)	82 (53·6)	165 (53·9)
Vaccinated Subjects	n (%)	70 (45·8)	71 (46·4)	141 (46·1)
Dose 1	n (%)	70 (45·8)	71 (46·4)	141 (46·1)
Dose 2	n (%)	60 (39·2)	57 (37·3)	117 (38·2)

Percentages were based on the total number of subjects in each treatment.

a Range of Ct values (14–35) were the same for both treatment groups.

b High-risk defined those unvaccinated or have co-morbidity or who are ≥ 45 years of age.

c Diabetes, hypertension, obesity, cardiovascular risk, or cerebrovascular risk.

### Efficacy

The high risk mITT adult population analyses results are the primary focus of the report, with other population and sensitivity results presented in the Appendix.

### Viral SARS-CoV-2 RNA reduction

SARS-CoV-2 RNA (mean [SD]) log10 copies/mL were reduced from 6·96 [1·51] (screening) to 5·86 [1·51] and 5·09 [1·93] in the NONS group at Day two (≤ 24 hours) and Day three (48 hours) on treatment, respectively. Mean viral RNA log10 copies/mL were reduced from 7·16 [1·53] (screening) to 6·71 [1·56] and 5·98 [1·70] in the placebo group at Day 2 and Day 3 on treatment, respectively (Table S6, Appendix).

After adjustment for baseline by MMRM, the mean viral RNA change from baseline was -1·20 [93·7% reduction (original anti-log scale)] and -1·98 [99·0% reduction] log10 copies/mL in the NONS group at Day 2 and Day 3 on treatment, respectively. The adjusted change from baseline in viral RNA log10 copies/mL at Day two and Day three on treatment with NONS were statistically superior to the change in placebo with a mean treatment difference [SE] of -0·82 [0·260] (6⋅6 fold improvement); 95% CI -1·33, -0·31 (*p* = 0·002) and -0·87 [0·270] (7⋅4 fold improvement); 95% CI -1·61, -0·12 (*p =* 0·022), respectively.

The primary efficacy endpoint was achieved in patients randomized to receive NONS compared to placebo in the high risk analysis population. The mean [SE] change from baseline in SARS-CoV-2 RNA burden through Day eight was -2·62 [0·145] log10 copies/mL in the NONS group (p<0·001). The average change from baseline in log viral RNA through Day eight for NONS was statistically superior to the change in the placebo group with a mean treatment difference [SE] of -0·52 [0·202] log10 copies/mL; 95% CI -0·92, -0·12; *p =* 0·010; (Table 2). The reductions observed were comparable in the unvaccinated mITT population and mITT population from baseline through Day eight (Table S7, Appendix). The high risk PP sensitivity analysis results support the primary endpoint (Table S8, Appendix). The effect size after adding vaccinated status as an additional covariate to the model (Table S9, Appendix), was comparable to the original model mITT high risk population (Table 2) and mITT population (Table S7, Appendix).

**Table 2:** Mean SARS-CoV-2 viral load (log10 copies per mL) change from baseline through day 8 in adult COVID-19 infected patients (mITT high risk population: MMRM).

Parameter	Statistics	NONS (*N =*64)	PLACEBO *N =*69)
Baseline (Day 1)	mean (SD)	6·96 (1·506)	7·16 (1·532)
	median	6·79	7·30
	minimum, maximum	2·56, 10·10	2·58, 10·81
Mean CFB through Day 3	LSM (SE)	-1·57 (0·199)	-0·74 (0·193)
	95% CI	-1·96, -1·17	-1·13, -0·36
	Difference: LSM (SE)	-0·82 (0·277)	
	95% CI of difference	-1·37, -0·27	
	*p*-value of difference	0·003	
Mean CFB through Day 4	LSM (SE)	-2·15 (0·171)	-1·48 (0·167)
	95% CI	-2·49, -1·82	-1·80, -1·15
	Difference: LSM (SE)	-0·68 (0·240)	
	95% CI of difference	-1·15, -0·21	
	*p*-value of difference	0·005	
Mean CFB through Day 8	LSM (SE)	-2·62 (0·145)	-2·10 (0·141)
	95% CI	-2·91, -2·34	-2·38, -1·83
	Difference: LSM (SE)	-0·52 (0·202)	
	95% CI of difference	-0·92, -0·12	
	*p*-value of difference	0·010	

CFB = Change from baseline.

Mean change from baseline in viral load log values through Day eight (7 days of therapy) was analysed by MMRM. The difference LSM(SE) between groups was calculated for NONS vs placebo (NONS-placebo). Patients were required to have a positive RT-PCR at Day one (screening) and Day two (≤ 24 post randomisation). The 95% confidence interval (CI) for the LSM mean difference between groups was calculated for NONS minus placebo. *p*-values were calculated for the comparison of treatment groups with treatment as main effect and by considering visit, baseline value, risk factor (high risk yes/no), and treatment by visit interaction as covariates.

There was a statistically significant change from baseline in the area under the curve (AUC) of the SARS-CoV-2 log10 viral RNA in the NONS group on Days three, four and eight (p < 0·001 on all days). Compared to the placebo group, the absolute change from baseline in the viral RNA normalized AUC was larger in the NONS group on Days three, four and eight with a mean [SE] treatment difference of -0·83 [0·250] log10 copies/mL on Day three (95% CI -1·32, -0·34; p<0·001), -0·70 [0·235] log10 copies/mL on Day four (95% CI -1·17, -0·24; *p =*0·003) and -0·54 [0·214] log10 copies/mL on Day eight (95% CI -0·96, -0·11; *p =*0·013) which confirms the finding of the mean primary endpoint (Table 3).

**Table 3:** Normalized AUC SARS-CoV-2 viral load (log10 copies per mL) change from baseline through day 8 in adult COVID-19 infected patients (mITT high risk population: MMRM).

Parameter	Statistics	NONS (*N =*64)	PLACEBO (*N =*69)
Mean AUC Day 2 - Day 3	LSM (SE)	-1·66 (0·178)	-0·83 (0·174)
	95% CI	-2·01, -1·31	-1·17, -0·48
	Difference: LSM (SE)	-0·83 (0·250)	
	95% CI of difference	-1·32, -0·34	
	p-value of difference	<0·001	
Mean AUC Day 2 - Day 4	LSM (SE)	-2·18 (0·168)	-1·48 (0·163)
	95% CI	-2·51, -1·85	-1·80, -1·15
	Difference: LSM (SE)	-0·70 (0·235)	
	95% CI of difference	-1·17, -0·24	
	p-value of difference	0·003	
fMean AUC Day 2 - Day 8	LSM (SE)	-2·66 (0·154)	-2·12 (0·149)
	95% CI	-2·96, -2·35	-2·41, -1·83
	Difference: LSM (SE)	-0·54 (0·214)	
	95% CI of difference	-0·96, -0·11	
	p-value of difference	0·013	

AUC  =  normalized area under the curve.

Difference LSM(SE) between groups was calculated for NONS vs placebo (NONS-placebo) by MMRM. Patients were required to have a positive RT-PCR at Day one (screening) and Day two (≤ 24 post randomisation). The 95% confidence interval (CI) for the LSM mean difference between groups was calculated for NONS minus placebo. *p*-values were calculated for the comparison of treatment groups with treatment as main effect and by considering visit, baseline value, risk factor (high risk yes/no), and treatment by visit interaction as covariates. Mean [SD] NONS baseline SARS-CoV-2 viral load was 6·96 [1·506] log10 copies/mL, and 7·16 [1·532] log10 copies/mL in the placebo group.

### Extent and rapidity of virologic recovery

**Figure 2:**
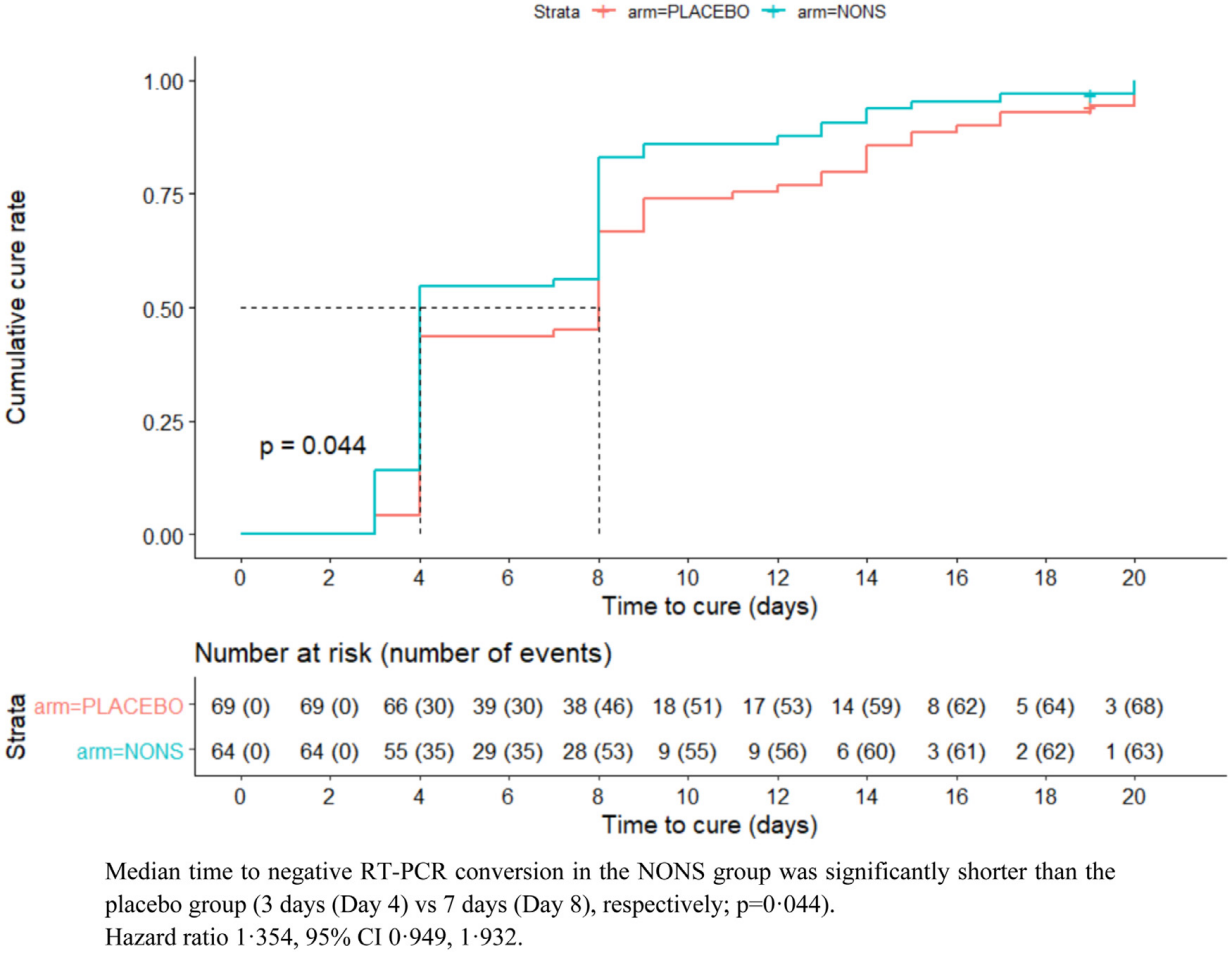
**Kaplan–Meier curve for time to negative conversion of SARS-CoV 2 RT PCR (mITT high risk population)**.

Secondary endpoint analysis revealed that a greater proportion of patients in the NONS group became qualitative RT-PCR negative compared to placebo. In the NONS group 53 (82·8%) subjects became negative compared to 46 (66·7%) by Day eight and this difference was statistically significant (16·1%, 95% CI 0·2, 32·1; *p =* 0·046). The median time to qualitative RT-PCR conversion, from positive to negative was significantly shorter in the NONS group by four days compared to placebo (three vs seven days [Day four vs Day eight]), Figure 2; *p =* 0·044. The median improvement time of four days to a negative conversion was also observed in the unvaccinated mITT population (*p =* 0·024) and mITT population (*p =* 0·351) (Table S10, Appendix).

### Clinical status improvement – investigator determined

**Figure 3:**
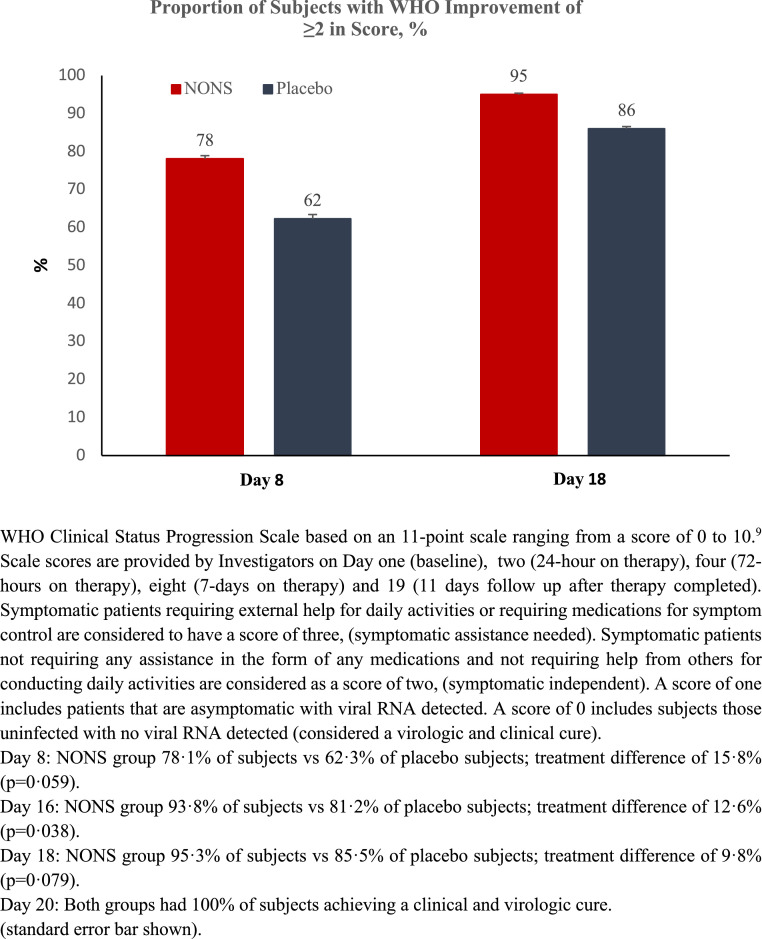
**Proportion of adult COVID-19 infected patients with ≥2 point clinical status improvement in WHO progression scale score (mITT high risk population)**.

A secondary endpoint analysis was conducted to evaluate the 2-point improvement in WHO CPS score in patients with baseline score of two or more (e.g., reduction lower from baseline). A greater proportion of subjects in the NONS group demonstrated a clinical status improvement compared to the placebo group over the 18-day study duration (Figure 3). In the NONS group, 50 (78·1%) subjects demonstrated improvement in WHO CPS score compared to 43 (62·3%) subjects in the placebo group by Day eight with a treatment difference of 15·8% (95% CI -1·0, 32·6; *p* = 0·059). By Day 16, 93·8% of NONS subjects demonstrated improvement in WHO CPS score compared to 81·2% subjects in the placebo group (treatment difference of 12·6%, 95% CI 0·1, 25·1; *p =* 0·038 [p-values provided for information only; not corrected for multiplicity]). By Day 18 the difference in clinical status improvement continued to converge; by Day 20 both groups had achieved a comparable clinical improvement without detectable nasal virologic RNA. The improvement in clinical status for the unvaccinated mITT population and mITT population although numeric followed the same course as the high risk mITT population (Table S11, Appendix).

### Hospitalizations required for the treatment of COVID-19

No adult COVID-19 infected patient in the study required hospitalization (or supplemental oxygen) for the treatment of COVID-19 by study end.

### Immediate contacts with patients becoming infected with COVID-19

**Figure 4:**
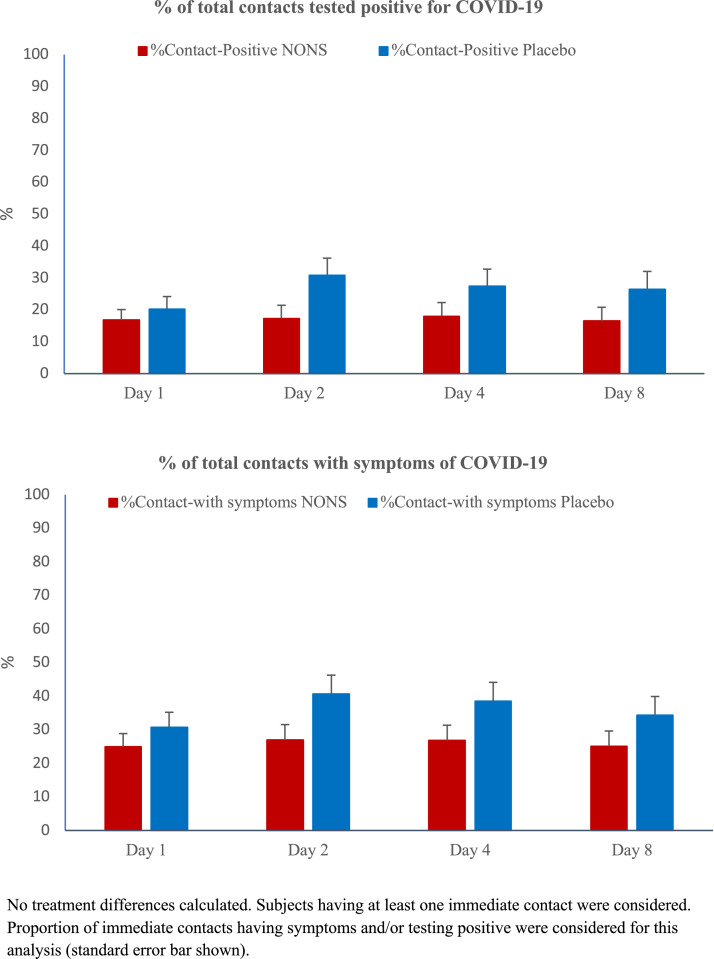
**Trial patients’ immediate contacts reporting testing COVID-19 positive and immediate contacts having COVID-19 symptoms (mITT high risk population)**.

The exploratory evaluation of the proportion of immediate contacts having a positive COVID-19 test or becoming symptomatic, remained nearly the same in the NONS group while it numerically increased in the placebo group over the treatment period (Figure 4).

### Safety

**Figure 5:**
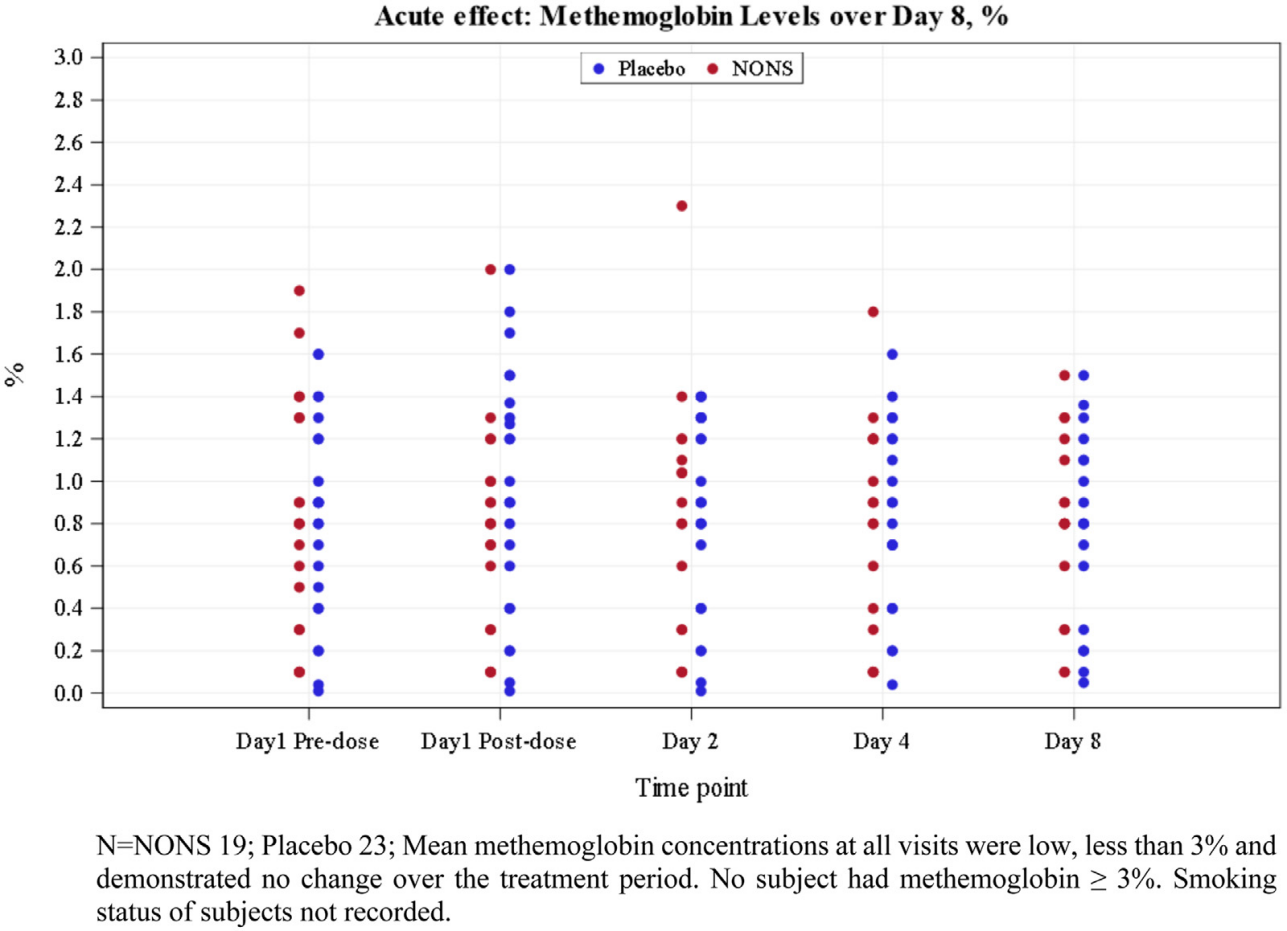
**Methemoglobin levels with NONS treatment at each visit day in adult COVID-19 patients**.

No SAE was reported. All AEs were mild in severity (Table 4). Nasal discomfort was the only infrequently observed respiratory AE in NONS subjects. No treatment related trends were observed in any vital sign, 12 Lead ECG parameter, laboratory parameter (chemistry, hematology, or urinalysis), SpO2 or physical examination. No clinically significant change from baseline in methemoglobin (MetHb) to each relevant time point was observed (Figure 5). No nasal vasodilation symptoms and no systemic vasodilation signs were observed in either treatment (Table S12, Appendix). No significant safety issue was associated with the use of nitric oxide nasal spray (NONS); the spray was well tolerated.

**Table 4:** Summary of adult COVID-19 infected patients with adverse events by treatment group (safety population).

System organ class preferred term	Statistics	NONS (*N =* 153) n (%)	PLACEBO (*N =* 153) n (%)	Total (*N =* 306) n (%)
Number of subjects with AEs	n (%) (y)	10 (6·5) (10)	3 (2·0) (3)	13 (4·2) (13)
Abdominal pain	n (%) (y)	1 (0·7) (1)	0	1 (0·3) (1)
Dyslipidaemia	n (%) (y)	2 (1·3) (2)	0	2 (0·7) (2)
Hypertriglyceridaemia	n (%) (y)	1 (0·7) (1)	0	1 (0·3) (1)
Myalgia	n (%) (y)	0	1 (0·7) (1)	1 (0·3) (1)
Nasal discomfort	n (%) (y)	5 (3·3) (5)	2 (1·3) (2)	7 (2·3) (7)
Rash	n (%) (y)	1 (0·7) (1)	0	1 (0·3) (1)

Adverse event terms were coded using MedDRA version 23·0 or latest. At each level of summarization a subject was counted once if the subject reported one or more events in a given level of summarization. Percentages were based on the total number of subjects in safety population in each treatment. y is the total number of events in safety population in each treatment.

## Discussion

Nitric oxide nasal spray (NONS) self-administered, in non-hospitalized adult Asian patients with mild symptomatic COVID-19 infections in this study, had a statistically significant greater mean reduction in SARS-CoV-2 RNA log10 copies/mL over the seven days of treatment compared to placebo in all populations. Efficacy was comparable in the unvaccinated population who lacked the benefits of vaccine immunity. Those participants vaccinated demonstrated no impact on the treatment effect of NONS, as expected, due to the evolving COVID-19 disease state, i.e., increased virus transmissibility, shortened infectivity period and inconsistent vaccine effectiveness with each new SARS-CoV-2 VOC.^12^^,^^13^

However, those at high risk are the current target of outpatient treatments to reduce illness progression as the virus continues to mutate despite an increasing global vaccination rate.^13^^,^^14^ The high risk population (defined as unvaccinated, ≥ 45 years of age, or had one or more comorbidities in the present study), demonstrated a profound SARS-CoV-2 RNA burden reduction of 93·7% at 24 hours and 99·0% at 48 h with NONS. NONS had a rapid effect of viral RNA reduction, i.e., a 7⋅4 fold greater viral RNA reduction compared to placebo at 48 h of treatment. Clinically, this is striking in that our patients had high baseline viral RNA concentrations (10^7^ copies/mL) associated with presumably the highest risk for additional illness progression.^15^

The chief assessment for this study was NONS administered to the high risk population resulted in a greater decline in viral RNA from baseline (Day 1) through Day 8 compared to placebo (mean -0·52, 95% CI -0·92, -0·12). Other outpatient therapies have shown slightly better treatment differences. An intravenously-administered monoclonal antibody therapy (casirivimab/imdevimab, 1200 mg daily, reduced viral load from baseline through Day 7 compared to placebo, a mean of -0·70, 95% CI -0·90, -0·53).^15^ An oral combination antiviral therapy (ritonavir [100 mg]-boosted nirmatrelvir [300 mg] twice daily, demonstrated a -0⋅9 log10 copies/mL greater decline than placebo in nasopharyngeal viral RNA through Day 5).^16^ We hypothesize the smaller than expected treatment difference in the current trial is due to the use of an active placebo, performed during a morphing SARS-CoV-2 VOC landscape, in subjects also had free access to hydroxychloroquine and azithromycin publicly during the trial; although use of hydrochloroquine was exclusionary). Our study included participants with vaccine breakthrough (46⋅1%), demonstrating the inability of vaccination to prevention infections during an outbreak of the Delta VOC (58% of participants) or combined Omicron/Delta VOCs (42% of participants).

A significantly greater proportion of high risk COVID-19 infected subjects receiving NONS had no measurable SARS-CoV-2 RNA (RT-PCR negative) by the end of treatment (82·8% of NONS subjects vs 66·7% of placebo; treatment difference 16⋅1%, 95% CI 0·2, 32·1; *p =* 0·046). NONS subjects were 35·4 % more likely to achieve RT-PCR negative status 4 days sooner (72 h on treatment) than the placebo group (Day 8), which is expected to result in significant illness burden reduction and be clinically relevant to patients, clinicians, employers, insurers and governments. To our knowledge no other outpatient antiviral therapies have as quickly produced this negative RT-PCR conversion. Clinically, more subjects receiving NONS were asymptomatic with no detectable SARS-CoV-2 RNA, based on the investigators’ WHO Clinical Progression Scale score (two or more point reduction), near the end of the study compared to placebo (Day 16 treatment difference 12·6%, 95% CI 0·1, 25·1; *p =* 0·038).

Disease severity and risk of COVID-19 progression correlates with the concentration of upper respiratory tract SARS-CoV-2.^17^, ^18^, ^19^, ^20^ Others have suggested that a shorter time to elimination of viral RNA will reduce the time of potential infectivity.^17^ NONS rapidly works within the nose to inactivate SARS-CoV-2 to potentially shorten the duration of an individual's infectivity, clinical infection trajectory and virus transmissibility (per adhoc analysis).^21^^,^^22^ Clinically, the use of NONS is expected to result in less use of medical resources, less loss of productivity and days absence from work, and the maintenance of quality of life (physical, mental and social; to be assessed in further Phase III trials). NONS use is not expected to be associated with the development of drug resistance or systemic drug-drug interactions, unlike those associated with other anti-SARS-CoV-2 therapies. An additional benefit of NONS is its activity against a broad spectrum of respiratory pathogens, including influenza.^23^

Limitations to this study include an exclusion of those > 70 years and children < 18 years of age, and a lack of separation of symptoms resolution between treatments due to the wide variability in the type of symptoms and their severity at illness onset. There was insufficient sample size to compare the impact of participant symptoms resolution and hospitalisations (low statistical power). The primary endpoint did not include deaths or hospitalizations, as the current risk of hospitalisation or death across continents is lower with every new VOC.^14^ Sample size will continue to increase substantially to meet this objective if used as a primary endpoint. Rather we believe smaller samples sizes focused on the reduction of RNA and speed to a negative RT-PCR can equate to efficacy. Currently approved treatments have typically demonstrated efficacy through a reduction of hospitalisation and deaths which has been associated with an accelerated clearance of nasal viral RNA. Antivirals unable to achieve a reduction in viral RNA are not approved by regulatory authorities or found in treatment guidelines.^24^ The placebo nasal spray used in the study contained a preservative (benzalkonium chloride). Data suggests normal saline and benzalkonium chloride have virucidal activity.^25^ The smaller than expected clinically important effect size, based on the sample size calculation, was likely impacted by the use of an active placebo.

The remarkable safety profile may be beneficial if used in the elderly, children, immunocompromised and pregnant/breastfeeding women. The significant reduction in SARS-CoV-2 accompanied by an early virological cure and symptomatic improvement (investigator determined) with nitric oxide nasal spray demonstrated in this study support the use of NONS for treatment of COVID-19 patients in those at risk of illness progression.

## Contributors

MT, WW, RK, AP, SR, KM, and CM were involved in the design or organization of the trial, or both. SW, YPT, and SJ provided the optimal methods for nasal swab technique and laboratory PCR method analyses. SK, AK, CRJ, RG, SN, RR, NB, AG, VP, DA, AN, VR, SK, ZP, and SB trained the study staff and implemented the methods for recruitment the study participants, sample collections, data collection and assessed the impact of treatment on the study participants. RK and AP were involved in data management. WW oversaw the generation and analyses of the data. WW, MT, RK, KM and CM analysed the data. KM and SW wrote the first draft of the manuscript. All authors contributed to the final version of the paper. MT, WW, RK, KM, SW, YPT, CM and SJ had full access to all the data in the study and had final responsibility for the decision to submit the manuscript for publication.

## Data sharing statement

The study protocol is available in the appendix. Individual participant data that underlie the reported results are not available.

## Declaration of interests

We declare no competing interests.

## Appendix Supplementary materials

Image, application 1

Image, application 2

Image, application 3

Image, application 4
