# Correction to “SARS-CoV-2 accelerated clearance using a novel nitric oxide nasal spray (NONS) treatment: A randomized trial”

**DOI:** 10.1016/j.lansea.2022.100110

**Published:** 2022-11-06

**Authors:** Monika Tandon, Wen Wu, Keith Moore, Stephen Winchester, Yuan-Po Tu, Christopher Miller, Rahul Kodgule, Amol Pendse, Shabbir Rangwala, Shashank Joshi

**Affiliations:** a Glenmark Pharmaceuticals Limited, Mumbai, India; b SaNOtize Research & Development Corp., Vancouver, British Columbia, Canada; c Frimley Health NHS Foundation Trust, Surrey, England; d The Everett Clinic-Part of Optum, Everett, Washington; e Glenmark Pharmaceuticals Limited, UK; f Lilavati Hospital and Research Centre, Mumbai, India

The authors wish to update the following statements in their original Article:

## Role of the funding source

Funding was provided by Glenmark Pharmaceuticals. Study medication was provided by SaNOtize. Glenmark Pharmaceuticals and SaNOtize played a role in study design, data collection and analysis, and interpretation; in the writing of the manuscript; and in the decision to submit the paper for publication.

## Data sharing statement

Individual participant data are not available publicly. De-identified individual participant data can be shared on receipt of reasonable request (eg, by regulatory authorities).

## Declaration of interests

MT, WW, RK, AP and SR are employees of Glenmark Pharmaceuticals Limited. MT is Senior Vice President at Glenmark. KM is employee of and CM is Co-Founder & Chief Scientific Officer at SaNOtize Research & Development Corp. SJ has been a speaker for Abbott, Alkem, AstraZeneca, Micro, Boeringher Ingelheim, Novo Nordisk, MSD, Sanofi, PHFI, Lupin, Eli Lilly, Bayer Zydus, Zydus Cadila and DRL; and on advisory board for Abbott, Glenmark, Biocon, Zydus Cadila, Bayer Zydus Twin Health, Marico, Franco India, Cipla, Sun, Torrent and USV. Y-PT has received investigator-initiated research grant and payment for presentations from Abbott. SW does not have any competing interests to declare.

