# Supplementary material for: SARS-CoV-2 accelerated clearance using a novel nitric oxide nasal spray (NONS) treatment: A randomized trial
Source: Lancet Reg Health Southeast Asia. 2022 Jun 29;3:100036. doi: 10.1016/j.lansea.2022.100036 (PMC9239922; doi:10.1016/j.lansea.2022.100036)
Supplement: Supplementary file 1 [file mmc1.pdf]

NITRIC OXIDE NASAL SPRAY

GPL/CT/2021/004/III

A RANDOMIZED, DOUBLE-BLIND, PARALLEL ARM, MULTICENTER STUDY TO EVALUATE THE EFFICACY AND SAFETY OF NITRIC OXIDE NASAL SPRAY COMBINED WITH STANDARD SUPPORTIVE CARE IN ADULT NON-HOSPITALIZED PATIENTS WITH COVID-19

**Phase of Development**	Phase 3
**Sponsor**	Glenmark Pharmaceuticals Limited,<br>Glenmark House,<br>BD Sawant Marg, Chakala,<br>Andheri East, Mumbai 400 099<br>India
**Protocol Number**	GPL/CT/2021/004/III
**Approved by**	Dr. Monika Tandon, MD, DM.<br>Senior Vice President and Head, Clinical Development
**Protocol Version**	Version: 6.0
**Date**	31-Dec-2021
**Supersedes**	Version 5.0 dated 04-Oct-2021

This study must be conducted in accordance with International Conference on harmonization (ICH) guidelines on Good Clinical Practice (GCP) and with the applicable regulatory requirements.

INVESTIGATOR'S AGREEMENT

I have received and read the Investigational Brochure (IB) for nitric oxide nasal spray. I have read the GPL/CT/2021/004/III and agree to conduct the study as outlined. I agree to maintain the confidentiality of all information received or developed in connection with this protocol.

____

Printed Name of Investigator

____

Signature of Investigator

____

Date

SPONSOR'S SIGNATURE

This protocol reflects the Sponsor's current knowledge of nitric oxide nasal spray as applicable to this study. It has been designed to achieve the stated objectives while minimizing exposure to, and risk from, both the products being used and the assessments. The assessments are all considered to be appropriate, capable of validating the stated objectives of the study, and of providing the necessary information to ensure subject safety. The protocol has been designed according to the principles of the ICH guidelines for GCP, Declaration of Helsinki, and local rules, acts and guidelines. It has undergone both medical and scientific review by the Sponsor. The Sponsor is responsible to regulatory authorities for taking all reasonable steps to ensure the proper conduct of the study as regards ethics, protocol compliance, integrity and validity of the data recorded on the electronic case report forms (eCRFs).

- We hereby agree to conduct the study in accordance with this protocol and the above-mentioned guidance/ regulation.
- We agree to comply with all relevant standard operating procedures (SOP) required for the conduct of this study.
- We further agree to ensure that all associates assisting in the conduct of study are informed regarding their obligations.

Signed on behalf of the Sponsor, Glenmark Pharmaceuticals Ltd.

Dr Rahul Kodgule
General Manager Clinical Development
Clinical Lead
Andheri (E), Mumbai-400099
Phone: +91 22 4018 9999
E-mail: rahul.kodgule@glenmarkpharma.com

Date: 31-Dec-2021

Reviewed and Approved by:

Monika Tandon

Dr. Monika Tandon, MD, DM
Senior Vice President and Head-Clinical development
Global Specialty/Branded Portfolio
Glenmark Pharmaceuticals Ltd.
Andheri East, Mumbai- 400099
E-mail: Monika.Tandon@glenmarkpharma.com

Date: 31-Dec-2021

2. SYNOPSIS

**Name of Sponsor/Company:** Glenmark Pharmaceuticals Limited.	
**Name of Study Drug:** Nitric Oxide Nasal Spray.	
**Title of Study**: A Randomized, Double-blind, Parallel Arm, Multicenter Study To Evaluate The Efficacy And Safety Of Nitric Oxide Nasal Spray Combined With Standard Supportive Care In Adult non-hospitalized Patients With COVID-19.	
**IND no:** Not applicable	**EudraCT no:** Not applicable
**Phase of development:** 3	
**Indication:** Treatment of COVID-19	
**Objectives:**<br>**Primary:**<br>• The primary objective of this study is to evaluate the efficacy of Nitric Oxide Nasal Spray combined with standard supportive care compared with standard supportive care alone in adult subjects with COVID-19 not requiring hospitalization<br>**Secondary:**<br>• The secondary objective is to evaluate the safety and tolerability of Nitric Oxide Nasal Spray combined with standard supportive care compared with standard supportive care alone in adult subjects with COVID-19 not requiring hospitalization.	
**Study population:** Patients with COVID-19 (confirmed by RT-PCR), not requiring hospitalization for the treatment of COVID-19 symptoms	
**Study design:**<br>This is a randomized, double-blind, multi-center, parallel arm, clinical study evaluating the efficacy and safety of Nitric Oxide Nasal Spray with standard supportive care vs standard supportive care alone in patients with COVID-19 not requiring hospitalization.<br>306 eligible patients will be randomized in a 1:1 ratio into 2 groups: one group will receive Nitric Oxide Nasal Spray (NONS) along with standard supportive care and the control group will receive standard supportive care along with placebo of NONS. Standard supportive care will be provided in accordance with latest guidelines issued by Ministry of Health and family welfare; Government of India. Treatment duration is 7 days (up to Day 8 visit) and the total study duration will be maximum for 18 days from randomization (up to Day 19 visit). Viral load will be estimated on Day 1 (baseline), Day 2 (post-24 hours of dosing), Day 3 (post 48 hours of dosing), Day 4 (post 72 hours of dosing) and Day 8. Patients may be detained in a facility to facilitate protocol assessments for first 7 days or may be treated at home based on investigator discretion. Subjects who are treated at home will visit the study center on Day 1, 4, and 8 for study assessments and Day 2 visit (and swab sample collection for visit 2) will be conducted at home. Day 3 will be laboratory visit only for collection of swab from both sides in the nose for RT-PCR. If subjects are not able to visit the study center on any of the days, telephonic or video-conference and home based assessments can be conducted.<br>On Day 1 (screening and randomization), written informed consent will be obtained from subjects with symptoms of COVID-19 and having symptom onset within 48 hours before consent. After informed consent, following activities will be conducted: detailed clinical history and physical examination, vaccination status, vital signs, SpO_2 measurement, 12-lead ECG, chest x-ray, eligibility assessment and blood and urine collection for laboratory assessments (including C-Reactive Protein). Rapid	

Antigen Test for COVID-19 will be conducted as part of screening assessment and subjects with positive result for COVID-19 antigen will be randomized. Subjects with negative result for COVID-19 antigen will be considered as screen failure. Only swab from both sides in the nose will be taken as per the standard procedure for quantitative and qualitative RT-PCR assessments. If the result of qualitative RT-PCR of swab sample collected during screening is negative, subject will be withdrawn from the study. After screening procedures subjects will be randomized to one of the two treatment arms and investigational product (IP) will be dispensed. After training on nasal spray administration, first dose will be self-administered under supervision. Standard supportive care will be provided to all the subjects during the study.

On Day 2, 3 and 4 swab from both sides in the nose will be taken for quantitative and qualitative RT-PCR. On Day 2 and 4 following study procedures will be conducted: adverse event review, concomitant medication review, IP compliance review, SpO_2, vital signs. On Day 4 a chest x-ray or CT scan will be done (choice between x-ray and CT scan at Day 4 will be based on investigator discretion). Only for subjects who are not able to visit the site can conduct chest x-ray or CT scan at nearby facility. Day 3 will be a Laboratory visit during which swab will be collected from nose for RT-PCR. On Day 8 subject will visit the study site and following activities will be performed: adverse event review, concomitant medication review, IP compliance review, SpO_2, vital signs, physical examination, 12-lead ECG, blood and urine collection for laboratory assessments. Swab from both sides in the nose will be taken for qualitative and quantitative RT-PCR assessment, and IP will be retrieved. Qualitative and quantitative RT-PCR assessments will be done in all subjects on days 2, 3 and 4, irrespective of RT-PCR result (positive or negative) on days 2 and 3. Subjects who have negative result of qualitative RT-PCR (RT-PCR negative) at Day 4, RT-PCR (both qualitative and quantitative) will not be done on Day 8 and rest of the assessments as per visit schedule for Day 8 will be conducted. Visit 5 will be conducted on Day 19 ± 2 days or any time between Day 8 and Day 19, if the subject's COVID-19 symptom status and RT-PCR result becomes negative. Visit 5 will be a telephonic visit or clinic visit. During visit 5, history of adverse events and concomitant medication will be taken and in case of adverse event a clinic visit can be performed based on investigator's discretion for detailed assessments or additional tests. For subjects who are RT-PCR positive on Day 8, standard of care will be continued and swab from both sides in the nose will be collected at visit 5 for qualitative RT-PCR assessment. On Day 1, 2, 4, and 8; and at visit 5 information about immediate contacts and their COVID-19 status will be collected. During the study, following events will be recorded: first time use of high flow supplemental oxygen, hospitalization for treatment purposes (COVID-19 related), non-invasive ventilation, mechanical ventilation, and extracorporeal membrane oxygenation and symptom scales. Methemoglobin will be measured non-invasively in a subset of patients on Day 1 [before randomization (baseline), and 5 minutes after administration of first dose of NONS], Day 2, Day 4 and Day 8. Subject diary will be used to record information related to adverse events, use of concomitant medications and study drug compliance. Subject diary will be dispensed at screening/randomization visit (visit 1) and retrieved at Day 8 (visit 4). Subjects will record their health status and COVID-19 related symptoms daily during the study participation using the subject diary. Investigator will record the score on the WHO Progression Scale at visits 1, 2, 3, 4 and 5. After Day 8 visit, additional qualitative RT-PCR tests can be conducted as unscheduled investigations in subjects who are RT-PCR positive on Day 8. Subjects whose baseline COVID-19 symptoms have resolved by Day 8 and whose RT-PCR is negative by Day 8, will not undergo any further assessment (including visit 5) and their study participation will be considered as complete. For subjects whose baseline COVID-19 symptoms are present at Day 8 and/or whose RT-PCR is positive at Day 8 will be continued in the study till maximum of Day 19. Between Day 8 and Day 19 on or after the day the subject becomes RT-PCR negative and has baseline COVID-19 symptoms resolved, visit 5 can be conducted and subject's study participation can be considered complete. All subjects study participation, irrespective of RT-PCR and symptom status will end on maximum of Day 19±2 days (visit 5).

Study endpoints:

Primary Endpoint:

- Change from baseline in log viral load through Day 8

Secondary Endpoints:

- Proportion of subjects with negative conversion of SARS-CoV 2 RT PCR on Day 2, 3, 4, and 8.
- Determine effect of NO nasal spray on clinical recovery [Time Frame: 18 days]. Determine the time to clinical recovery in participants with COVID-19 by measuring the proportion of patients from enrollment to resolution of baseline flu-like symptoms.
- Proportion of subjects requiring hospitalization for the treatment of COVID-19 [Tim frame: 18 days]
- Proportion of patients achieving a 2 point worsening in WHO Progression scale on Day 2, 4, 8 and 19
- Time from randomization to first time use of high flow supplemental oxygen/non-invasive ventilation/mechanical ventilation/ extracorporeal membrane oxygenation.
- Change from baseline in COVID-19 related symptom score of participants with COVID- 19 at Day 2, 3, 4, 8 and 19
- Change from baseline in log Viral load at Day 2, 3, 4, and 8 [Time Frame: 7 days]
- Safety and tolerability of 7 day administration of NO nasal spray treatment over 18 days [Time Frame: 18 days]. Measure the tolerability of the NO nasal spray treatment as determined by number of adverse events, pain, discomfort or discontinuations of treatment.

Number of subjects (planned): 306 subjects will be randomized in the study

Main criteria for inclusion:

Each subject must meet all of the following criteria to be entered into the randomized treatment in the study:

1. Voluntarily participating in the clinical study; fully understanding and being fully informed of the study and having signed the Informed Consent Form (ICF); willingness and capability to complete all the study procedures
2. Age 18-70 years (inclusive) at the time of signing ICF
3. Patients with laboratory confirmation of infection with SARS-CoV-2 by positive Rapid Antigen Test for SARS-CoV-2 at screening.
4. Recent onset (within ~72 hours of time of consent) symptoms of mild COVID-19 with oxygen saturation (SpO_2 > 94 %) and respiratory rate < 24 breaths/min. Any of the COVID-19 like symptoms including fever, cough, sore throat, malaise, headache, nasal congestion, muscle pain, gastrointestinal symptoms, lack of taste or smell without shortness of breath or dyspnea; (the maximum permitted difference in the time of onset of symptoms and the time of consent is ~72 hours)
5. For female subjects: evidence of post-menopause, or, for pre-menopause subjects, negative pretreatment urine pregnancy test
6. Eligible subjects of child-bearing age (female or male with female partner of childbearing age) must agree to take effective contraceptive measures (including hormonal contraception, barrier

methods or abstinence) with his/her partner during the study period and for at least 7 days following the last study treatment.<br>7. Not participating in any other interventional drug clinical studies before completion of the present study.<br>**Main criteria for exclusion:**<br>A subject who meets any of the following criteria must not be entered into the randomized treatment in the study:<br>1. Where, in the opinion of the investigator, participation in this study will not be in the best interest of the subject, or any other circumstances that prevent the subject from participating in the study safely<br>2. Subjects with infection requiring oxygen support, invasive or non-invasive ventilator support, extracorporeal membrane oxygenation (ECMO) or shock requiring vasopressor support.<br>3. Current known pneumonia based on x-ray or computed tomography (CT) scan or history of pneumonia within 3 months before screening.<br>4. Requiring hospitalization for the treatment of COVID-19<br>5. Prolonged QT, defined as QTcF ≥ 450 milliseconds for men and as QTcF ≥ 470 milliseconds for women<br>6. History of known severely reduced left ventricular (LV) function (Ejection fraction < 30 %)<br>7. Requires ICU care for management of ongoing clinical status.<br>8. Known allergy or hypersensitivity to Nitric Oxide Nasal Spray.<br>9. History of known severe renal impairment [creatinine clearance (CrCl) < 30 mL/min] or having received continuous renal replacement therapy, hemodialysis or peritoneal dialysis;<br>10. Asthma, allergic rhinitis or chronic obstructive lung disease<br>11. Psychiatric disease that is not well controlled (controlled defined as stable on a regimen for more than one year).<br>12. Pregnant or lactating women;<br>13. Having used Nitric Oxide Nasal Spray or participated in any other interventional drug clinical study within 30 days prior to first dose of study drug.<br>14. Subjects who have received hydroxychloroquine within 7 days before screening or subjects who require hydrochloroquine treatment.<br>15. Subjects who have received intranasal medication/treatment within 7 days before screening or subjects who require use of any intranasal medication.<br>16. Subjects who have received medications with antiviral effect such as remdesivir, favipiravir, oseltamivir, ivermectin or inhaled corticosteroids within 7 days before screening or subjects who require use of any of these medications<br>17. Subjects using nitric oxide donor agents such as prilocaine, sodium nitroprusside and nitroglycerine
**Duration of study participation:** The anticipated maximum total study duration for each subject is approximately 18 days (up to day 19 visit). This will consist of the treatment period of up to 7 days (up to day 8 visit), and the follow-up period of maximum 11 days after the last dose of study drug.
**Duration of treatment:** 7 days.
**Investigational product, dosage and mode of administration:**

Nitric oxide nasal spray, administered as a nasal spray, at a dose of two sprays each nostril, six times a day (along with standard supportive care). Study medication should be administered upon awakening (Dose 1), then administer additional doses (Dose 2 to 6) approximately every 2-3 hours while awake. Doses should be separated by at least 1.5 hours. Preferably, administer the last dose of the day (Dose 6) at bedtime.<br>Investigational Drug Products will be Manufacture and Supplied by SaNOtize Research and Development Corp. 25th Floor, 700 West Georgia Street, Vancouver, BC, Canada [Manufactured at Nextar Chempahrm Solutions Ltd. 13B Einstein St. Weizmann Science Park, Ness Ziona 74140 Israel.
**Reference therapy :** Placebo of Nitric oxide nasal spray, administered as a nasal spray, at a dose of two sprays each nostril, six times a day along with Standard supportive care

**STATISTICAL METHODS:**	Statistical Methods are summarized below and details of methods and plan will be provided in the statistical analysis plan (SAP).
**Study Populations:**	**Analysis Sets**<br>Analysis of the primary endpoint will be conducted using the modified ITT (mITT) analysis set. In addition, a supportive analysis will be performed for the primary efficacy endpoint using the Per Protocol Set (PPS).<br>**mITT analysis set**<br>The mITT analysis set will include all randomised subjects who received at least one dose of study medication, who have a non-missing baseline measurement and at least one post-baseline efficacy measurement for primary efficacy variable.<br>**Per protocol analysis set**<br>The per protocol analysis set (PPS) will include all subjects who are randomized, received at least one dose of study medication, completed the study and do not have any major protocol deviations. Major protocol deviations will be discussed and decided at the blinded data review meeting (BDRM) meeting before database lock.<br>**Safety analysis set**<br>The Safety analysis set (SAS) will include all subjects who are randomized and received at least one dose of study medication. All safety endpoints will use the safety analysis set unless otherwise specified.
**Determination of Sample Size:**	Based on the data of phase 2 study of Nitric Oxide Nasal Spray in the United Kingdom (Clinical Study Report IRAS ID 287727 NONS COVID Study), assuming a treatment effect of 5.0 log viral load in the primary endpoint, with a standard deviation of 10.0, 172evaluable subjects (86 subjects per arm) will provide a power of 90% with two-sided significance level of 5 %. For the secondary endpoint (RT-PCR conversion), assuming RT-PCR conversion of 40% in the Placebo group by Day 8, 260 subjects (130 subjects in each arm) will provide a power of 90% at two-sided significance level of 5 %, to detect a treatment difference of 20% in proportion of subjects achieving RT-PCR conversion.<br>Assuming a dropout rate of 15 %, total 306 subjects (approx. 153 subjects per arm) will be randomized.

**Efficacy Analyses:**	The primary endpoint will be analyzed in the mITT population as the primary analysis. PP set will be used as sensitivity analysis. The details of analysis methods for primary and secondary endpoints will be provided in the statistical analysis plan (SAP).
**Pharmacokinetic Analyses:**	Not applicable
**Pharmacodynamic Analyses:**	Not applicable
**Biomarker Analyses:**	Not applicable
**Pharmacogenomic Analyses:**	Not applicable
**Safety Analyses:**	All safety analyses will be performed using the safety analysis set (SAS) and will be presented by the study treatment. Safety data will be summarized descriptively. Data describing quantitative measures will be summarized as mean, SD, median and range (minimum and maximum). Qualitative variables will be presented as counts and percentages. All safety data will also be displayed in listings.
**Interim analyses:** Interim analysis will be performed when ~50 % of the subjects complete the study according to group sequential design and sample size will be re-assessed. Based on the pre specified analysis and as per Subject Expert Committee (SEC) recommendation dated 29/12/2021, to perform another analysis of current recruited subjects to include high risk population. The details will be described in the SAP.	

3. TABLE OF CONTENTS, LIST OF TABLES, AND LIST OF FIGURES

TABLE OF CONTENTS

1. TITLE PAGE 1
2. SYNOPSIS 4
3. TABLE OF CONTENTS, LIST OF TABLES, AND LIST OF FIGURES 10
4. LIST OF ABBREVIATIONS AND DEFINITIONS OF TERMS 15
5. INTRODUCTION 17
5.1. Background Information 17
5.2. Description of Study Drug 19
5.3. Nonclinical Experience 19
5.3.1. Nonclinical Pharmacology 19
5.3.2. Nonclinical Pharmacokinetics and Metabolism 20
5.3.3. Toxicology 20
5.4. Clinical Experience 21
5.5. Benefit-Risk Assessment 22
6. STUDY OBJECTIVES AND PURPOSE 25
6.1. Primary Objective 25
6.2. Secondary Objective 25
7. INVESTIGATIONAL PLAN 26
7.1. Overall Study Design 26
7.1.1. Rationale for Study Design, Dose(s) and Comparator(s) 28
7.1.2. Estimated Duration of Subject Participation 28
7.2. Number of Subjects 28
7.3. Treatment Assignment 29
7.4. Dose Adjustment Criteria 29
7.5. Criteria for Study Termination 29
7.6. End of the Study 29
8. SELECTION AND WITHDRAWAL OF SUBJECTS 33
8.1. Subject eria 33
8.2. Subject Exclusion Criteria 33
8.3. Subject Withdrawal Criteria 34

8.3.1. Lost to Follow-up 35
8.3.2. Permanent Discontinuation of Study Drug 35
8.3.3. Replacement of Subjects 35
9. TREATMENT OF SUBJECTS 37
9.1. Description of Study Drug 37
9.2. Concomitant Medications 38
9.2.1. Prior Medications 38
9.2.2. Permitted Concomitant Medications 38
9.2.3. Prohibited Concomitant Medications 38
9.2.4. Lifestyle and/or Dietary Restrictions 39
9.3. Treatment Compliance 39
9.4. Randomization and Blinding 40
9.4.1. Un-blinding 40
9.5. Subject Completion 41
10. STUDY DRUG MATERIALS AND MANAGEMENT 42
10.1. Study Drug 42
10.1.1. Investigational Product 42
10.1.2. Placebo 42
10.2. Study Drug Packaging and Labeling 42
10.3. Study Drug Storage 43
10.4. Study Drug Preparation 43
10.5. Administration 44
10.6. Study Drug Accountability 44
10.7. Study Drug Handling and Disposal 44
11. ASSESSMENT OF EFFICACY 45
11.1. Quantitative RT-PCR: 45
11.2. Qualitative RT-PCR: 45
11.3. WHO Progression Scale: 45
11.4. COVID-19 Related Symptoms Score: 46
12. ASSESSMENT OF SAFETY 48
12.1. Safety Parameters 48
12.1.1. Demographic/Medical History 48

12.1.2. Vital Signs 48
12.1.3. Weight and Height 48
12.1.4. Physical Examination 49
12.1.5. Electrocardiogram 49
12.1.6. Laboratory Assessments 49
12.1.6.1. Hematology 49
12.1.6.2. Blood Chemistry 49
12.1.6.3. Urinalysis 50
12.1.6.4. Pregnancy Screen/testing 50
12.2. Adverse and Serious Adverse Events 50
12.2.1. Definition of Adverse Events 50
12.2.1.1. Adverse Event 50
12.2.1.2. Assessment of Severity of Adverse Events 51
12.2.1.3. Serious Adverse Event 51
12.3. Relationship to Study Drug 52
12.4. Recording Adverse Events 53
12.4.1. Collection of Adverse Events 53
12.4.2. Recording of Adverse Events 54
12.5. Reporting Serious Adverse Events 54
12.5.1. Pregnancy 55
13. TIMING OF STUDY ASSESSMENTS 55
13.1. Screening and Randomization: Visit 1 (Day 1) 55
13.2. Treatment Visit: Visit 2 (Day 2) and Visit 3 (Day 4) 57
13.3. End of Treatment (EOT) Visit: Visit: 4 (Day 8 + 1) 57
13.4. Early Withdrawal Visit 58
13.5. Post Treatment Follow-Up (Telephonic or Clinic Visit): Visit 5 (Day 19±2) 59
14. STATISTICS 61
14.1. Sample Size 61
14.2. Analysis Sets 61
14.2.1.1. Per Protocol Set 61
14.2.1.2. mITT analysis set 61
14.2.1.3. Safety Analysis Set 62

14.3. Endpoints 62
14.3.1. Primary Endpoint 62
14.3.2. Secondary Endpoint(s) 62
14.4. Subject Disposition 62
14.5. Demographic and Other Baseline Characteristics 62
14.6. Efficacy Analyses 63
14.7. Pharmacokinetic, Pharmacodynamic, Biomarker, and Pharmacogenomic/Pharmacogenetic Analyses 63
14.8. Safety Analyses 63
14.8.1. Extent of Exposure 63
14.8.2. Adverse Events 63
14.8.3. Laboratory Values 63
14.8.4. Vital Signs 63
14.8.5. Electrocardiograms 64
14.8.6. Other Safety Analyses 64
14.9. Other Analyses 64
14.10. Interim Analysis 64
14.11. Data Safety Monitoring Committee 64
15. DIRECT ACCESS TO SOURCE DATA/DOCUMENTS 65
15.1. Study Monitoring 65
15.2. Audits and Inspections 65
15.2.1. Inspection 65
15.2.2. Audit 66
15.3. Institutional Review Board/Independent Ethics Committee 66
16. QUALITY CONTROL AND QUALITY ASSURANCE 67
17. ETHICS 68
17.1. Ethics Review 68
17.2. Ethical Conduct of the Study 68
17.3. Written Informed Consent 68
18. DATA HANDLING AND RECORDKEEPING 70
18.1. Data Collection 70
18.2. Inspection of Records 70
18.3. Retention of Records 70

18.4. Financing and Insurance 70
19. PUBLICATION POLICY 71
20. LIST OF REFERENCES 72
21. APPENDICES 74
APPENDIX 1. LIST OF CONTACT DETAILS 75
APPENDIX 2. CLINICAL LABORATORY TESTS 76
APPENDIX 3. WHO PROGRESSION SCALE 78

LIST OF TABLES

Table 1: Abbreviations and Specialist Terms 15
Table 2: Schedule of Assessments 30
Table 3: Study Drugs 37
Table 5: Clinical Laboratory Tests 76

LIST OF FIGURES

Figure 1: Study Design 28

4. LIST OF ABBREVIATIONS AND DEFINITIONS OF TERMS

The following abbreviations and specialist terms are used in this study protocol.

Table 1: Abbreviations and Specialist Terms

Abbreviation or Specialist Term	Explanation
AE	adverse event
ALP	alanine aminotransferase
AST	aspartate aminotransferase
ACE2	Angiotensin-converting enzyme 2
BP	Blood pressure
BIPAP	Bilevel positive airway pressure
CT	computerized tomography
CBC	complete blood count
CSR	clinical study report
CXR	chest X-ray
CrCl	creatinine clearance
CPAP	continuous positive airway pressure
COVID-19	coronavirus disease of 2019
DMC	Data Monitoring Committee
DSMB	Data Safety Monitoring Board
eCRF	electronic case report form
ECG	Electrocardiogram
ECMO	extracorporeal membrane oxygenation
FiO_2	fraction of inspired oxygen
GCP	Good Clinical Practice
GGT	Gamma-glutamyl transferase
HIV	Human immunodeficiency viruses
IP	investigational product
ICF	informed consent form
ICH	International Conference on Harmonization
ICU	Intensive care unit
IEC	independent Ethics Committee
IRB	Institutional Review Board

Abbreviation or Specialist Term	Explanation
(IVRS/IWRS)	(interactive voice response system/interactive web-based response system)
K	potassium
LV	Left ventricular
LAR	legally acceptable representative
mITT	Modified intended to treat
MedDRA	Medical Dictionary for Regulatory Activities
MV	mechanical ventilation
Na	sodium
NIV	Non-invasive ventilation
NONS	Nitric Oxide Nasal Spray
pH	potential hydrogen
pO_2	partial pressure of oxygen
PaO_2	arterial oxygen partial pressure
PPS	Per protocol set
ppm	Parts per million
QT	Electrocardiographic QT interval from onset of Q wave to end of T wave
QTc	QT interval corrected for HR
RNA	ribonucleic acid
RT-PCR	Reverse transcription polymerase chain reaction
SAE	serious adverse event
SAS	Safety analysis set
SAP	Statistical Analysis Plan
SARS-CoV2	Severe acute respiratory syndrome coronavirus 2
SpO_2	peripheral capillary oxygen saturation
ULN	Upper limit of normal
WHO	World health organization

5. INTRODUCTION

5.1. Background Information

Coronavirus disease 2019 (COVID-19) is a global pandemic caused by severe acute respiratory syndrome coronavirus 2 (SARS-CoV-2). SARS-CoV-2 is a highly transmissible and pathogenic coronavirus that emerged in late 2019. In India, more than 30 million people have been infected and currently there are > 600,000 active cases (Govt. of India). The disease also affects the lung causing viral pneumonia and its complications, acute lung injury and acute respiratory distress syndrome, leading to large number of deaths. More than 390,000 people have died with COVID-19 in India. The second wave of the pandemic has been a major public health concern with more than 350,000 cases being detected every day and exhausting the hospital and healthcare infrastructure.

Multiple studies have demonstrated that higher viral load of SARS-CoV-2 is associated with more severe disease, risk of intubation, longer intubation period, longer stay in intensive care unit and risk of death (Maltezou HC et al, J Infect Dis 2021; Fajnzylber J et al, Nature Communications 2020; Pujadas E et al, Lancet Respir Med 2020). High nasopharyngeal viral load of SARS-CoV-2 is also associated with increased risk of infection transmission (Kawasuji H et al, PLoS One 2020; Marks M et al, Lancet Infect Dis 2021). Also, the peak viral load occurs close to the time of onset of symptoms and declines over a period of few days (Bullard J et al, Clin Infectious Dis 2020; Benefield AE et al, MedRxiv Review 2020). Thus, viral load plays a crucial role in COVID-19 disease severity, outcomes and risk of infection transmission. Hence, there is an urgent need of an effective treatment option that can help in reducing viral load.

Development of a new molecule is a long and complex process taking about 10 years to bring a molecule from conceptualization to market (Development Timelines, Drug Development and Delivery). Hence, there is need to re-purpose already available treatments. Nitric oxide is a gaseous molecule naturally produced in humans and is also present in ambient atmosphere. It is known to have pulmonary vasodilator properties and is already approved for the treatment of neonates with hypoxic respiratory failure associated with pulmonary hypertension (Genosyl US Prescribing Information). The dose of nitric oxide released from the NO Nasal Spray is 200 times smaller than the dose approved by the US FDA in neonates. Significant anti-viral effect against SARS-CoV-2 and its variants has been demonstrated at this dose.

SaNOtize Research and Development Corporation, Canada has developed Nitric Oxide Nasal Spray (NONS) for COVID-19. The nasal spray is currently approved in Israel and Bahrain and is under process of registration globally. SaNOtize has collaborated with Glenmark Pharmaceuticals Limited for development of the NONS in India for treatment and prevention of COVID-19.

Nitric oxide (NO) has also been found to have broad spectrum antiviral effects including against SARS-CoV-2. Anti-viral effect of NO has been reported in a variety of viral infections, including H1N1, Influenza A & B, HIV, vaccinia virus, enterovirus and coronavirus (Fang W et al, Free Radic Biol Med 2021; Hermann E et al., Int. J. Immunopharm 1997; Mĕlkov´a Z et al, J. Immunol. 1995; Haagmans BL et al, Antivir 2006). In vitro tests conducted using SaNOtize's NORS formulation inactivated more than 99.9 % of SARS-CoV-2 [to below limit of detection < 0.7 CCID50 (cell culture infectious dose 50 %) per 0.1 mL], within two minutes [Study No. SARS2-049, 2021]. NO also prevents the fusion between the S protein and its cognate receptor, ACE-2 [Akerström, S., et al., 2009]. Inhibitory effect of nitric oxide donors SARS-CoV-1 infection in VeroE6 cells was demonstrated by a Swedish group. The nitric oxide donor significantly

inhibited the replication cycle of SARS-CoV-1 at both RNA and cellular levels in a dose-dependent manner (Akerstrom S et al, Virology 2005).

A pilot study of nitric oxide in patients with SARS CoV infection, showed that inhaled NO could shorten the time of ventilator support for patients infected with SARS-CoV. Inhaled NO improved arterial oxygenation and enabled the reduction of inspired oxygen therapy. In addition, chest radiography showed decreased lung infiltrates, and the physiological effects remained even after termination of inhaled NO therapy, suggesting, not only a pulmonary vasodilator effect of inhaled NO, but also an anti-viral effect (Chen L et al, Clin Infect Dis 2004).

In a randomized, open label clinical trial in 29 Indian patients with COVID-19 pneumonia and having hypoxic respiratory failure, Moni M et al, evaluated the anti-viral effects of inhaled nitric oxide (Moni M et al, MedRxiv 2021). Nitric oxide was administered at doses 20 to 80 ppm over a maximum duration of 30 minutes each day for 3 days. There was a significant difference in the viral load reduction in viral load iNO cases compared to controls ($p < 0.002$), demonstrating superior anti-viral effect of inhaled nitric oxide. The proportion of patients achieving 2-point improvement in the WHO Ordinal Scale within 14 days of randomization was significantly higher in the inhaled NO group (79 %), as compared to the controls (36 %) ($p = 0.05$). Small sample size being a limitation of the study, it is difficult to conclude and generalize the efficacy results of the study. No adverse events in the NO group were reported during the study.

Efficacy and safety of inhaled nitric oxide nasal spray (NONS) in 79 patients with mild COVID-19 infection was evaluated in a randomized, double-blind, placebo controlled, parallel group, phase 2 clinical trial conducted in the UK (Clinical Study Report of study IRAS ID 287727 NONS COVID Study; EudraCT 2020-004994-27). In this study, NONS was administered over a period of 9 days with follow-up up to 18 days. The primary endpoint was: difference in SARS-CoV-2 viral load from baseline through day 6 between NONS and control arms. In this study, early treatment with NONS significantly reduced the level of SARS-CoV-2, including in patients with high viral loads. The average viral log reduction in the first 24 hours was 1.362, which corresponds to a decline of about 95 %. Within 72 hours, the viral load dropped by more than 99 %. Viral loads were 16 times lower in the group that received NONS compared to those who received placebo. No serious or severe adverse health effects were reported in the NONS group in the study. In another clinical trial conducted in Canada, no serious or severe adverse health effects were reported in over 7000 self-administered treatments (Clinical Study Report of study COVID-CTP-01). In this trial, 103 people were given the nasal spray and nobody tested positive for COVID-19.

Thus, nitric oxide nasal spray is a promising and locally acting therapeutic agent for the treatment or prevention of COVID-19. Given its relatively low cost, ready availability and excellent safety profile, nitric oxide may be a game changer in COVID-19 management (Alhareth D et al, Int J Respir Pulmon Med 2020). This phase 3 clinical trial is planned based on the UK clinical trial of SaNOtize (IRAS ID 287727 NONS COVID Study Protocol) that demonstrated reduction of viral load as early as 24 hours.

Glenmark plans to conduct this phase 3 clinical trial to evaluate safety, tolerability and efficacy of nitric oxide nasal spray in patients with COVID-19 who do not require hospitalization for the treatment of COVID-19

5.2. Description of Study Drug

Nitric oxide is a colorless gas with sharp sweet odor and can be administered by inhalation. It is a pulmonary vasodilator has also been found to have antiviral effects not limited to specific virus and reported in a variety of viral infections, including SARS-CoV-2, H1N1, Influenza A & B, HIV, vaccinia virus, enterovirus and coronavirus (Fang W et al, Free Radic Biol Med 2021; Hermann E et al., Int. J. Immunopharm 1997; Mělkov′a Z et al, J. Immunol. 1995; Haagmans BL et al, Antivir 2006). NO is an important mediator in intracellular inhibition of viral replication, which results in lower viral yields and more efficient host clearance of the infection (Reiss CS et al, J Virol 1998).

The product is a nasal spray, which needs to be sprayed 2 times into each nostril.

5.3. Nonclinical Experience

5.3.1. Nonclinical Pharmacology

Nitric oxide (NO) has been found to have antiviral effects not limited to specific virus and reported in a variety of viral infections, including human immunodeficiency virus (HIV), vaccinia virus, enterovirus and coronavirus (Fang, et al., 2021). NO is an important mediator in intracellular inhibition of viral replication, which results in lower viral yields and more efficient host clearance of the infection, hence recovery (Reiss and Komatsu, 1998).

The NO donor significantly inhibited the replication cycle of SARS-CoV-1 at both RNA and cellular levels in a dose-dependent manner. In the in vitro study, SARS-CoV-1 infected Vero E6 cells treated with S-nitroso-N-acetylpenicillamine (SNAP) (a NO-donor) showed that SNAP significantly inhibited the replication cycle of SARS-CoV-1 at both RNA and cellular levels in a dose-dependent manner and decreased offspring viruses by 82%. SARS-CoV-2 (COVID-19) and SARS-CoV-1 share a similar infection process: they both rely on the membrane fusion mediated by the viral S protein with the host cell receptor ACE2 to promote the injection of viral genetic material (Åkerstrom, et al., 2005). Therefore, the inhibition of SARS-CoV-2 by NO may be similar to that of SARS-CoV-1(Fang, et al., 2021).

Nitric oxide relaxes vascular smooth muscle by binding to the haeme moiety of cytosolic guanylate cyclase, activating guanylate cyclase and increasing intracellular levels of cGMP, which then leads to vasodilation. In isolated rat lungs, and in awake and anaesthetised animals as lambs, sheep, rabbit, rat and dog, NO produced a dose-dependent pulmonary vasodilatation over a large range of concentrations (doses from 5 to 120 ppm have been tested). Pulmonary vasoconstriction evoked by activation of the coagulation system (endotoxin, lipopolysaccharide) or by infection (group B *streptococcu*s) was also reversed by NO inhalation at doses up to 150 ppm in piglets. In anaesthetised mechanically ventilated guinea pigs, 300 ppm NO decreased methacholine-induced bronchoconstriction. Histamine-induced bronchoconstriction in dogs was also reversed at doses between 100-200 ppm. Studies have shown that NO promotes or inhibits almost every stage of inflammation. The bactericidal effect of NO involves DNA damage, protein modification, inhibition of the mitochondrial electron transport chain or other metabolic pathway enzymes (INOmax-Scientific Discussion, 2005).

In safety pharmacology studies, the major findings included an anticipated increase in methaemoglobin and ECG abnormalities in dogs after inhalation administration of NO. The ECG abnormalities were not observed in another study in dogs up to 320 ppm of NO. These effect were

most probably due to the fact that dogs may be more susceptible to cardiac irritability than human. The NO effect on the immune system in the mouse consisted of an initial increase in some parameters followed by a subsequent decrease; this may be secondary to stress or to the uncontrolled NO_2 levels ((INOmax-Scientific Discussion, 2005).

5.3.2. Nonclinical Pharmacokinetics and Metabolism

In rats, the percentage of absorption of NO decreased with increasing concentrations, being 90 %, 60 % and 20 % at 138, 270, and 380 ppm, respectively. The major metabolic pathway of inhaled NO is that the inhaled gas combines with hemoglobin, forming nitrosyl-haemoglobin (NOHb) from which, nitrites (NO_2) and nitrates (NO_3) are generated. In the presence of oxygen, there is a rapid oxidation of NOHb into methemoglobin and the subsequent reduction by methemoglobin reductase of methemoglobin into ferrous hemoglobin and nitrate. Nitrites and nitrates are in majority excreted in the urine; a small amount is discharged in the oral cavity via the salivary glands. Nitrite is converted to N_2 gas in the stomach. Nitrate in the intestine is partly reduced to ammonia (NH_3), re-absorbed in the body and converted to urea. Most of the metabolites of inhaled NO are excreted from the body within 48 hours (INOmax-Scientific Discussion, 2005).

5.3.3. Toxicology

In acute (mouse, rat and dog) and repeat (mouse, rat and rabbit) inhalation toxicity studies, the main findings were the formation of methaemoglobin. Deaths were attributed to methaemoglobin-induced tissue anoxia. The results that NO might cause lung pathology were however not consistent. The potential of additive toxicity, which may be associated with high concentrations of oxygen and NO_2 in the range of 0.25 to 2.0 ppm, have been investigated in the rat model, there appears to be no treatment related adverse effects (NOmax-Scientific Discussion, 2005; INOmax-Product Monograph, 2017)).

In a 4-week study, rats were exposed to concentrations of 0, 40, 80, 160, 200 and 250 ppm of NO via a nose-only inhalation system 6 hours/day for 28 consecutive days. Mortality was noted at ≥ 200 ppm. Methaemoglobin levels were elevated in rats exposed at ≥ 160 ppm. Histopathological examination did not detect changes that could be attributed to NO. Electron microscopic examination revealed slight ultrastructural changes of ciliated respiratory, type 2 alveolar, and Clara cells at 200 ppm which was consistent due to NO_2 exposure (INOmax-Scientific Discussion, 2005; INOmax-Product Monograph, 2017)). No treatment-related effects were noted at 80 ppm of NO (80-fold the NO exposure at maximum daily dose [MDD] of 1 ppm NO).

Positive genotoxic potential for NO have been demonstrated in several genotoxicity assays including Ames test and chromosomal aberration test (human lymphocytes). The positive results in several genotoxicity assays were noted at higher concentration of NO (Ames test: ≥ 1580 ppm; Mouse lymphoma assay: > 125 ppm; Chromosome aberration study in CHO cells: 1500-1800 ppm). The systemic exposure of NO to human at these higher concentrations are unlikely to be achieved with proposed MDD of Nitric Oxide Nasal Spray (NO level is not expected to exceed 1 ppm at MDD). Further no evidence of a clastogenic effect in peripheral blood lymphocytes of human volunteers were noted in the *in vivo* chromosome aberration study at inhalation exposure at 40 ppm NO (40-fold the NO exposure at MDD of 1 ppm) (INOmax-Scientific Discussion, 2005; INOmax-Product Monograph, 2017). No evidence of a carcinogenic effect was apparent, at inhalation exposures up to 20 ppm, in rats for 20 hr/day for up to two years (INOmax-Product Monograph, 2017; INOMAX-USPI, 2019). There are no reproductive animal

studies or human studies to evaluate NO for effects on fertility or harm to the developing fetus (INOmax-Product Monograph, 2017; (INOMAX-USPI, 2019). However taking into account NO is a compound produced endogenously by many cells in human body and long history of clinical use of inhaled NO (up to 20 ppm), its short half-life (~ 45 seconds) and significantly low proposed MDD of 1 ppm of NO, there is unlikely any safety concern on reproductive system. No local irritation of respiratory tract was seen following repeat dose nose-only inhalation toxicity study in mice, rats and rabbits.

The proposed MDD of 1 ppm of NO (two sprays each nostril for 5 times daily) from Nitric Oxide Nasal Spray is 20-fold lower than the previously approved inhaled clinical dose of 20 ppm, hence no safety concerns are anticipated with current use of Nitric Oxide Nasal Spray. The upper limit of exposure (mean exposure) to NO for personnel defined by worker's legislation is 25 ppm for 8 hours (30 mg/m^3) which is 25-fold higher than that of MDD of 1 ppm of NO delivered through Nitric Oxide Nasal Spray (INOmax-Product Monograph, 2017).

Based on long history of clinical use of inhaled NO and established upper limit of exposure worldwide, the safety of proposed MDD of 1 ppm of NO delivered through Nitric Oxide Nasal Spray is well established.

5.4. Clinical Experience

Nitric oxide is an endogenous gaseous molecule naturally produced in humans. It is known to have pulmonary vasodilator properties and is already approved for the treatment of neonates with hypoxic respiratory failure associated with pulmonary hypertension (INOmax US Prescribing Information; Genosyl US Prescribing Information).

Inhibitory effect of nitric oxide donors SARS-CoV-1 infection in VeroE6 cells was demonstrated by a Swedish group. The nitric oxide donor significantly inhibited the replication cycle of SARS-CoV-1 at both RNA and cellular levels in a dose-dependent manner (Akerstrom S et al, Virology 2009). Two mechanisms have been proposed for NO-mediated antiviral effects: (1) nitric oxide affects one or two replication-related cysteine proteases encoded by SARS-CoV-1 ORF1a, which directly inhibits viral RNA replication, and (2) nitric oxide decreases the palmitoylation level of S protein and inhibited the membrane fusion of offspring virus S protein binding to host cell receptor ACE2 (Akerstrom S et al, Virology 2009). SARS-CoV-2 and SARS-CoV-1 share a similar infection process: they both rely on the membrane fusion mediated by the viral S protein with the host cell receptor ACE2 to promote the injection of viral genetic material (Letko M et al, Nat Microbiol 2020). Therefore, the inhibition of SARS-CoV-2 by NO may be similar to that of SARS-CoV-1 (Fang W et al, Free Radic Biol Med 2021).

A pilot study of nitric oxide in patients with SARS CoV infection, showed that inhaled NO could shorten the time of ventilator support for patients infected with SARS-CoV. Inhaled NO improved arterial oxygenation and enabled the reduction of inspired oxygen therapy. In addition, chest radiography showed decreased lung infiltrates, and the physiological effects remained even after termination of inhaled NO therapy, suggesting, not only a pulmonary vasodilator effect of inhaled NO, but also an anti-viral effect (Chen L et al, Clin Infect Dis 2004).

In a randomized, open lable clinical trial in 29 Indian patients with COVID-19 pneumonia and having hypoxic respiratory failure, Moni M et al, evaluated the anti-viral effects of inhaled nitric oxide (Moni M et al, MedRxiv 2021). Nitric oxide was administered at doses 20 to 80 ppm over a maximum duration of 30 minutes each day for 3 days. There was a significant difference in the

viral load reduction in viral load iNO cases compared to controls ($p < 0.002$), demonstrating superior anti-viral effect of inhaled nitric oxide. The proportion of patients achieving 2-point improvement in the WHO Ordinal Scale within 14 days of randomization was significantly higher in the inhaled NO group (79 %), as compared to the controls (36 %) ($p=0.05$). No adverse events were reported during the study.

Efficacy and safety of inhaled nitric oxide nasal spray (NONS) in 79 patients with mild COVID-19 infection was evaluated in a randomized, double-blind, placebo controlled, parallel group, phase 2 clinical trial conducted in the UK (Clinical Study Report of study IRAS ID 287727 NONS COVID Study; EudraCT 2020-004994-27). In this study, NONS was administered over a period of 9 days with follow-up up to 18 days. The primary endpoint was difference in SARS-CoV-2 viral load from baseline through Day 6 between NONS and control arms. In this study, early treatment with NONS significantly reduced the level of SARS-CoV-2, including in patients with high viral loads. The average viral log reduction in the first 24 hours was 1.362, which corresponds to a decline of about 95 %. Within 72 hours, the viral load dropped by more than 99 %. Viral loads were 16 times lower in the group that received NONS compared to those who received placebo. No adverse health effects were found in the study. In another clinical trial conducted in Canada, no adverse health effects were reported in over 7000 self-administered treatments (Clinical Study Report of study COVID-CTP-01). In this trial, 103 people were given the nasal spray and nobody tested positive for COVID-19.

5.5. Benefit-Risk Assessment

Nitric oxide is an endogenous gaseous molecule naturally produced in humans. It is known to have pulmonary vasodilator properties and is already approved for the treatment of neonates with hypoxic respiratory failure associated with pulmonary hypertension. The recommended dose is 20 ppm, maintained for up to 14 days (INOmax US Prescribing Information; Genosyl US Prescribing Information).

Efficacy and safety of inhaled nitric oxide was evaluated in several studies involving 325 patients (neonates) receiving nitric oxide doses of 5 to 80 ppm, titrated and administered by continuously over 24 hours a day for up to 14 days and 251 patients received placebo. Total mortality in the pooled trials was 11 % on placebo and 9 % on nitric oxide. From all controlled studies, at least 6 months of follow-up is available for 278 patients who received INOmax and 212 patients who received placebo. Among these patients, there was no evidence of an adverse effect of treatment on the need for re-hospitalization, special medical services, pulmonary disease, or neurological sequelae. From all controlled studies, at least 6 months of follow-up is available for 278 patients who received nitric oxide and 212 patients who received placebo. Among these patients, there was no evidence of an adverse effect of treatment on the need for re-hospitalization, special medical services, pulmonary disease, or neurological sequelae. In the NINOS study, treatment groups were similar with respect to the incidence and severity of intracranial hemorrhage, Grade IV hemorrhage, periventricular leukomalacia, cerebral infarction, seizures requiring anticonvulsant therapy, pulmonary hemorrhage, or gastrointestinal hemorrhage. In CINRGI, the only adverse reaction (> 2 % higher incidence on nitric oxide than on placebo) was hypotension (14 % vs. 11 %). In the NINOS study, treatment groups were similar with respect to the incidence and severity of intracranial hemorrhage, Grade IV hemorrhage, periventricular leukomalacia, cerebral infarction, seizures requiring anticonvulsant therapy, pulmonary hemorrhage, or gastrointestinal hemorrhage. Total mortality in the pooled trials was 11 % on placebo and 9 % on INOmax, a result

adequate to exclude INOmax mortality being more than 40 % worse than placebo. Total mortality in the pooled trials was 11 % on placebo and 9 % on INOmax, a result adequate to exclude INOmax mortality being more than 40 % worse than placebo. Total mortality in the pooled trials was 11 % on placebo and 9% on INOmax, a result adequate to exclude INOmax mortality being more than 40 % worse than placebo.

Nitric oxide, being highly reactive, has a very short half-life of 0.5 to 1 seconds in blood, and is rapidly converted to nitrate and methemoglobin. The half-life of NO depends on such surrounding conditions as the type and amount of oxygen-derived radicals, the pO_2, the pH, the concentration of transition metals, thiols (Kelm M, Biochimica Biophysica Acta 1999). Due to its short half-life nitric oxide is generally safe.

NO is an important mediator in intracellular inhibition of viral replication, which results in lower viral yields and more efficient host clearance of the infection, hence recovery (Reiss CS et al, J Virol 1998). Antiviral effects of Nitric oxide (NO) are not limited to specific virus and have been reported in a variety of viral infections, including HIV, vaccinia virus, enterovirus and coronavirus (Fang W et al, Free Radic Biol Med 2021; Hermann E et al., Int. J. Immunopharm 1997; Mĕlkov′a Z et al, J. Immunol. 1995; Haagmans BL et al, Antivir 2006).

In another study, nitric oxide donors significantly inhibited the replication cycle of SARS-CoV-1 at both RNA and cellular levels in a dose-dependent manner (Akerstrom S et al, Virology 2009). SARS-CoV-2 and SARS-CoV-1 share a similar infection process: they both rely on the membrane fusion mediated by the viral S protein with the host cell receptor ACE2 to promote the injection of viral genetic material (Letko M et al, Nat Microbiol 2020). Therefore, the inhibition of SARS-CoV-2 by NO may be similar to that of SARS-CoV-1 (Fang W et al, Free Radic Biol Med 2021).

A pilot study of nitric oxide in patients with SARS CoV infection, showed that inhaled NO could shorten the time of ventilator support for patients infected with SARS-CoV. Inhaled NO improved arterial oxygenation and enabled the reduction of inspired oxygen therapy. In addition, chest radiography showed decreased lung infiltrates, and the physiological effects remained even after termination of inhaled NO therapy, suggesting, not only a pulmonary vasodilator effect of inhaled NO, but also an anti-viral effect (Chen L et al, Clin Infect Dis 2004).

In a randomized, open label clinical trial in 29 Indian patients with COVID-19 pneumonia and having hypoxic respiratory failure, Moni M et al, evaluated the anti-viral effects of inhaled nitric oxide. Nitric oxide was administered at doses 20 to 80 ppm over a maximum duration of 30 minutes each day for 3 days. There was a significant difference in the viral load reduction in viral load iNO cases compared to controls ($p < 0.002$), demonstrating superior anti-viral effect of inhaled nitric oxide. The proportion of patients achieving 2-point improvement in the WHO Ordinal Scale within 14 days of randomization was significantly higher in the inhaled NO group (79 %), as compared to the controls (36 %) ($p=0.05$). No adverse events were reported during the study.

Efficacy and safety of inhaled nitric oxide nasal spray (NONS) in 79 patients with mild COVID-19 infection was evaluated in a randomized, double-blind, placebo controlled, parallel group, phase 2 clinical trial conducted in the UK (EudraCT 2020-004994-27). In this study, NONS was administered over a period of 9 days with follow-up up to 18 days. The primary endpoint was difference in SARS-CoV-2 viral load from baseline through Day 6 between NONS and control arms. In this study, early treatment with NONS significantly reduced the level of SARS-CoV-2, including in patients with high viral loads. The average viral log reduction in the first 24 hours was

1.362, which corresponds to a decline of about 95 %. Within 72 hours, the viral load dropped by more than 99 %. Viral loads were 16 times lower in the group that received NONS compared to those who received placebo. No adverse health effects were found in the study. In another clinical trial conducted in Canada, no adverse health effects were reported in over 7000 self-administered treatments (Clinical Study report of IRAS ID 287727 NONS COVID Study; EudraCT 2020-004994-27). In this trial, 103 people were given the nasal spray and nobody tested positive for COVID-19 (Clinical Study Report of study COVID-CTP-01).

As nitric oxide nasal spray is locally acting, efficacy is expected to be higher in terms of reducing viral load at most relevant nasal site and it is expected to be a safe treatment. Thus, the potential benefits of nitric oxide in terms of reduction in viral load of SARS CoV-2 virus, outweigh the potential risks.

6. STUDY OBJECTIVES AND PURPOSE

6.1. Primary Objective

The primary objective of this study is to evaluate the efficacy of Nitric Oxide Nasal Spray combined with standard supportive care compared with standard supportive care alone in adult subjects with COVID-19 not requiring hospitalization.

6.2. Secondary Objective

The secondary objective is to evaluate the safety and tolerability of Nitric Oxide Nasal Spray combined with standard supportive care compared with standard supportive care alone in adult subjects with COVID-19 not requiring hospitalization.

7. INVESTIGATIONAL PLAN

7.1. Overall Study Design

This is a randomized, double-blind, multi-center, parallel arm, clinical study evaluating the efficacy and safety of Nitric Oxide Nasal Spray with standard supportive care vs standard supportive care alone in patients with COVID-19 not requiring hospitalization.

306 eligible patients will be randomized in a 1:1 ratio into 2 groups: one group will receive Nitric Oxide Nasal Spray (NONS) along with standard supportive care and the control group will receive standard supportive care along with placebo of NONS. Standard supportive care will be provided in accordance with latest guidelines issued by Ministry of Health and family welfare; Government of India. Therapies recommended by current treatment guidelines (MoHFW, GoI 22 Apr 2021) are discussed in permitted medications section (Section 9.2.2).

Treatment duration is a maximum of 7 days (Day 1 visit to Day 8 visit) and the total study duration will be maximum for 18 days from randomization (up to Day 19 visit). Subjects will continue to receive study treatment till Day 8, irrespective of virological or clinical cure status. Patients may be detained in a facility to facilitate protocol assessments for first 7 days or may be treated at home based on investigator discretion. Subjects who are treated at home will visit the study center on days 1, 4 and 8 for study assessments. Day 3 will be laboratory visit only for collection of swab from both sides in the nose for RT-PCR. If subjects are not able to visit the study center on any of the days, telephonic or video-conference and home based assessments can be conducted.

On Day 1 (screening and randomization), written informed consent will be taken obtained from subjects with symptoms of COVID-19 and having symptom onset within 48 hours before consent. After informed consent, following activities will be conducted: detailed clinical history and physical examination, vaccination status, vital signs, SpO_2 measurement, 12-lead ECG, chest x-ray, eligibility assessment and blood and urine collection for laboratory assessments (including C-Reactive Protein). Rapid Antigen Test for COVID-19 will be conducted as part of screening assessment and subjects with positive result for COVID-19 antigen will be randomized. Subjects with negative result for COVID-19 antigen will be considered as screen failure. Swab from both sides in the nose will be taken as per the standard procedure for quantitative and qualitative RT-PCR assessments. If the result of qualitative RT-PCR of swab sample collected during screening is negative, subject will be withdrawn from the study. After screening procedures subjects will be randomized to one of the two treatment arms and IP will be dispensed. After training on nasal spray administration, first dose will be self-administered under supervision. Standard supportive care will be provided to all the subjects during the study.

On Day 2, 3 and 4 swab from both sides in the nose will be taken for quantitative and qualitative RT-PCR. On Day 2 and 4 following study procedures will be conducted: adverse event review, concomitant medication review, IP compliance review, SpO_2, vital signs. On Day 4 a chest x-ray or CT scan will be done (choice between x-ray and CT scan at Day 4 will be based on investigator discretion). Only for subjects who are not able to visit the site can conduct chest x-ray or CT scan at nearby facility. Day 3 will be a Laboratory visit during which swab will be collected from nose for RT-PCR. Subject will take last dose of study medication on Day 8 during visit. On Day 8 subject will visit the study site and following activities will be performed: adverse event review, concomitant medication review, IP compliance review, SpO_2, vital signs, physical examination,

12-lead ECG, blood and urine collection for laboratory assessments. Swab from both sides in the nose will be taken for quantitative and qualitative RT-PCR assessment, and IP will be retrieved. Qualitative and quantitative RT-PCR assessments will be done in all subjects on days 2, 3 and 4, irrespective of RT-PCR result (positive or negative) on days 2 and 3. Subjects who have negative result of qualitative RT-PCR (RT-PCR negative) at Day 4, RT-PCR (both qualitative and quantitative) will not be done on Day 8 and rest of the assessments as per visit schedule for Day 8 will be conducted.

Visit 5 will be conducted on Day 19 ± 2 days or any time between Day 8 and Day 19, if the subject's COVID-19 symptom status and RT-PCR result becomes negative. Visit 5 will be a telephonic visit. During visit 5, history of adverse events and concomitant medication will be taken and in case of adverse event a clinic visit can be performed based on investigator's discretion for detailed assessment or additional tests. For subjects who are RT-PCR positive on Day 8, standard supportive care will be continued a swab from both sides in the nose will be collected at visit 5 for qualitative RT-PCR assessment. On Day 1, 2, 4, and 8; and at visit 5 information about immediate contacts and their COVID-19 status will be collected. During the study, following events will be recorded: first time use of high flow supplemental oxygen, hospitalization (COVID-19 related) for treatment purposes, non-invasive ventilation, mechanical ventilation, and extracorporeal membrane oxygenation and symptom scales. Methemoglobin will be measured non-invasively in a subset of patients on Day 1 [before randomization (baseline), and 5 minutes after administration of first dose of NONS], Day 2, Day 4 and Day 8. Subject diary will be used to record information related to adverse events, use of concomitant medications and study drug compliance. Subject diary will be dispensed at screening/randomization visit (Visit 1) and retrieved at Day 8 (Visit 4). Subjects will record their health status and COVID-19 related symptoms daily during the study participation using the subject diary. Investigator will record the score on the WHO Progression Scale at visits 1, 2, 3, 4 and 5. After Day 8 visit, additional qualitative RT-PCR tests can be conducted as unscheduled investigations in subjects who are RT-PCR positive on Day 8. Subjects whose baseline COVID-19 symptoms have resolved by Day 8 and whose RT-PCR is negative by Day 8, will not undergo any further assessment (including visit 5) and their study participation will be considered as complete. For subjects whose baseline COVID-19 symptoms are present at Day 8 and/or whose RT-PCR is positive at Day 8 will be continued in the study until up to a maximum of Day 19. Between Day 8 and Day 19 on or after the day the subject becomes RT-PCR negative and has baseline COVID-19 symptoms resolved, visit 5 can be conducted and subject's study participation can be considered complete. All subjects study participation, irrespective of RT-PCR and symptom status will end on maximum of Day 19±2 days (visit 5).

See Figure 1 for a schematic diagram of the study design and Table 2 for the Schedule of Assessments.

The endpoints to be measured in this study are described in Section 14.3.

Figure 1: Study Design

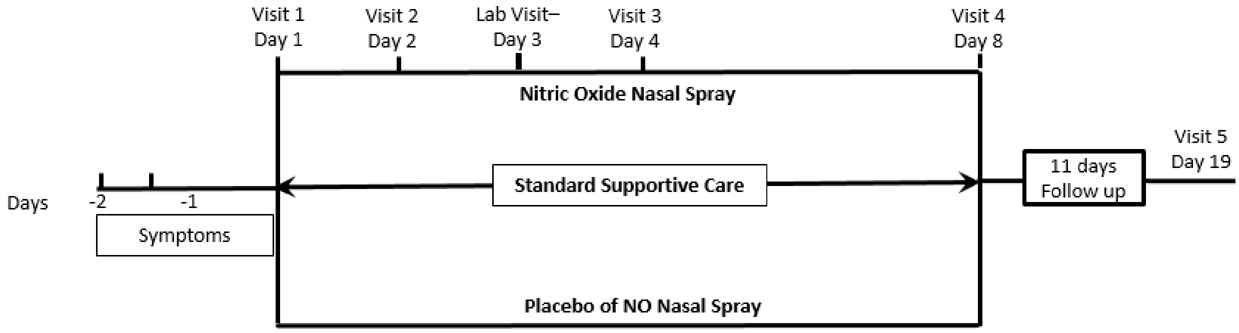

7.1.1. Rationale for Study Design, Dose(s) and Comparator(s)

This is a randomized, double-blind, placebo controlled, parallel group clinical trial. Randomized and double blind allocation of treatment is the most robust, appropriate and unbiased method to assess the efficacy and safety of an investigational product. As the study is planned in subjects with infectious disease, parallel group design is most appropriate. Use of placebo as a comparator allows to measure natural course of disease in absence of investigational treatment and helps to account for placebo effect.

Nitric oxide (NO) is endogenously produced naturally in human body and virtually every cell. The approved of inhaled NO is 20 ppm in neonates. Dose concentrations of 20 to 80 ppm administered continuously over 24 hours for up to 14 days to 6 months have been demonstrated to be safe and well tolerated (INOmax and Genosyl US Prescribing Information) The dose concentration of NO in the nitric oxide nasal spray (NONS) to be used in this study is 0.1 ppm. The dose of NO in the NONS is 200 times lower than the approved dose of inhaled NO in neonates (20 ppm). Hence, there is wide safety margin and the dose of NO in NONS is based mainly on the minimum effective dose. Anti-viral studies have demonstrated that this dose is adequate to reduce viral load of SARS-CoV-2 by > 99 %. Phase 2 clinical trial conducted in the UK has demonstrated anti-viral efficacy with this dose in patients with COVID-19, by reducing viral load by > 95 % in 24 hours. This dose has also been demonstrated to be safe and well tolerated in this (Clinical Study Report IRAS ID 287727 NONS COVID Study EudraCT 2020-004994-27). Hence, dose selected in this phase 3 study is appropriate.

7.1.2. Estimated Duration of Subject Participation

The anticipated maximum total study duration for each subject is approximately 18 days. This will consist of the treatment period of up to 7 days (up to Day 8 visit), and the follow-up period of maximum 11 days after the last dose of study drug (up to Day 19 visit). Subjects will receive study treatment even after negative RT-PCR for up to Day 8 visit.

7.2. Number of Subjects

Total **306 subjects** (approx. 153 subjects per arm) will be randomized.

7.3. Treatment Assignment

Subjects will be assigned to treatments based on a computer-generated randomization scheme that will be reviewed and approved by an independent statistician. The randomization scheme and identification for each subject will be included in the final clinical study report for this study.
Eligible subjects will be assigned with a unique randomization code at randomization visit.
If a subject withdraws from participation in the study, his/her enrolment/randomization code will not be reused. Randomization codes will be assigned strictly sequentially as subjects become eligible for randomization.

7.4. Dose Adjustment Criteria

Not applicable.

7.5. Criteria for Study Termination

The Sponsor reserves the right to discontinue the study for medical reasons or any other reason at any time. If a study is prematurely terminated or suspended, the Sponsor will promptly inform the Investigators/Institutions and regulatory authorities of the termination or suspension and the reason(s) for the termination or suspension. The Institutional Review Board (IRB)/independent Ethics Committee (IEC) will also be informed promptly and provided the reason(s) for the termination or suspension by the Sponsor or by the Investigator/Institution, as specified by the applicable regulatory requirement(s).

The Investigator reserves the right to discontinue the study should his/her judgment so dictate. If the Investigator terminates or suspends a study without prior agreement of the Sponsor, the Investigator should inform the Institution where applicable, and the Investigator/Institution should promptly inform the Sponsor and the IRB/IEC and provide the Sponsor and the IRB/IEC with a detailed written explanation of the termination or suspension. Study records must be retained as noted above.

7.6. End of the Study

The end of the study (study completion) is defined as the date of the last protocol-specified visit/assessment (including telephone contact) for the last subject in the study. For an individual subject, study participation will end at visit 4, if the subject’s baseline COVID-19 symptoms have resolved and subject is RT-PCR negative at visit 4. No further visits (including visit 5) will be conducted for such subjects. Subjects whose baseline COVID-19 symptoms are present and/or whose RT-PCR is positive at visit 4 will be followed up until resolution up to a maximum day 19 (±2 days) when visit 5 (end of study visit) will be conducted. However, if such subjects achieve status of COVID-19 symptoms resolved and RT-PCR negative any time between visit 4 and day 19, visit 5 can be conducted on such day (between visit 4 and day 19) and the subject’s study participation will end on that day.

Table 2: Schedule of Assessments

**Study Period**	**Treatment Period**					**Early Withdrawal Visit**[a]	**Post-Treatment Follow-Up (Telephonic or clinic visit[f])**
	**Screening & Randomization**				**End of Treatment (EOT)**		
**Visit**	**1**	**2**	**Lab Visit – Day 3**	**3**	**4**		**5**
**Time Point (days)**	**1**	**2**	**3**	**4**	**8+1**		**19±2** (or anytime between visit 4 and day 19)
Written informed consent	X						
Demographics	X						
Medical and surgical history	X						
Review COVID-19 Symptoms and clinical status	X	X		X	X	X	X
Prior and concomitant medications	X	X		X	X	X	X
Physical examination	X				X	X	
Height and Weight	X						
Vital signs, including SpO_2[b]	X	X		X	X	X	
12-lead ECG[c]	X				X	X	
Hematology, serum chemistry, urinalysis	X				X	X	
C-Reactive Protein	X						

Chest X-ray[h]	X			X			
Methemoglobin[d]	X	X		X	X	X	
Quantitative RT PCR (swab from both sides in the nose)	X	X	X	X	X	X	
Qualitative RT PCR (swab from both sides in the nose)	X	X	X	X	X	X	X[g]
Review inclusion/exclusion criteria	X						
Randomization	X						
Pregnancy test[e]	X				X	X	
Assessment of AEs/SAEs	X	X		X	X	X	X
WHO Progression Scale Review	X	X		X	X	X	X
Spray Bottle Use Perception Questionnaire	X	X			X	X	
Subject Diary dispensing	X						
Subject Diary Review		X		X	X	X	
Investigational product/study drug dispensing	X						
Drug and Diary accountability		X		X	X	X	

AE = adverse event; ECG = electrocardiogram; SAE = serious adverse event.

[a] Early withdrawal visit to be performed if applicable. If early withdrawal visit is performed on the day of scheduled visit, then both scheduled visit and early withdrawal visit assessments will be performed on the same day. Follow-up visit should be performed 11±2 days after early withdrawal visit, if there is subject's consent.

[b] If visits 2 (day 2) or 3 (day 4) are performed as home-based visits, blood pressure measurements and physical examination are not required

[c] For 12-Lead ECG, if any clinically significant abnormality is detected, an additional triplicate ECG will be recorded.

[d]Methemoglobin assessment will be performed in only a subset of patients. In this subset, methemoglobin will be measured on day 1 (before randomization and 5 minutes after first dose of study medication), and days 2, 4 and 8

[e] Female subjects only. Urine and Serum pregnancy test will be performed at Screening and urine pregnancy test will be performed at visit 4 and early withdrawal visit.

[f] Visit 5 will only be conducted for patients whose baseline COVID-19 symptoms are present at visit 4 and/or whose RT-PCR is positive at visit 4. Visit 5 can be conducted any time between visit 4 and day 19, based on subject's status change to symptom free and RT-PCR negative. If there is no change in such status, visit 5 will be conducted on day 19 ± 2 days. If clinic visit is performed on Day 19, any additional assessments made can be recorded on unscheduled visit eCRF page, at the discretion of the investigator

[g] Swab from both sides in the nose will be collected for qualitative RT-PCR at visit 5 only if RT-PCR is positive on Day 8 visit 4. If RT-PCR is negative at visit 3 (Day 4) no swab will be collected for RT-PCR on Day 8 visit 4 and at visit 5.

[h] Only chest x-ray will be conducted at screening visit. At visit 3 (day 4), based on investigator discretion, chest x-ray or CT scan can be performed.

8. SELECTION AND WITHDRAWAL OF SUBJECTS

8.1. Subject Inclusion Criteria

Each subject must meet all of the following criteria to be entered into the randomized treatment in the study:

1. Voluntarily participating in the clinical study; fully understanding and being fully informed of the study and having signed the Informed Consent Form (ICF); willingness and capability to complete all the study procedures
2. Age 18-70 years (inclusive) at the time of signing ICF
3. Patients with laboratory confirmation of infection with SARS-CoV-2 by positive Rapid Antigen Test for SARS-CoV-2 at screening.
4. Recent onset (within ~72 hours of time of consent) symptoms of mild COVID-19 with oxygen saturation ($SpO_2 > 94$ %) and respiratory rate < 24 breaths/min. Any of COVID-19 like symptoms including fever, cough, sore throat, malaise, headache, nasal congestion, muscle pain, gastrointestinal symptoms, lack of taste or smell without shortness of breath or dyspnea; (the maximum permitted difference in the time of onset of symptoms and the time of consent is ~72 hours)
5. For female subjects: evidence of post-menopause, or, for pre-menopause subjects, negative pretreatment urine pregnancy test
6. Eligible subjects of child-bearing age (female or male with female partner of child-bearing age) must agree to take effective contraceptive measures (including hormonal contraception, barrier methods or abstinence) with his/her partner during the study period and for at least 7 days following the last study treatment;
7. Not participating in any other interventional drug clinical studies before completion of the present study.

8.2. Subject Exclusion Criteria

A subject who meets any of the following criteria must not be entered into the randomized treatment in the study:

1. Where, in the opinion of the investigator, participation in this study will not be in the best interest of the subject, or any other circumstances that prevent the subject from participating in the study safely
2. Subjects with infection requiring oxygen support, invasive or non-invasive ventilator support, ECMO or shock requiring vasopressor support.
3. Current known pneumonia based on x ray or CT scan or history of pneumonia within 3 months before screening.
4. Requiring hospitalization for the treatment of COVID-19
5. Prolonged QT, defined as QTcF ≥ 450 milliseconds for men and as QTcF ≥ 470 milliseconds for women
6. History of known severely reduced left ventricular (LV) function (Ejection fraction < 30 %)

7. Requires ICU care for management of ongoing clinical status.
8. Known allergy or hypersensitivity to Nitric Oxide Nasal Spray
9. History of known severe renal impairment [creatinine clearance (CrCl) < 30 mL/min] or having received continuous renal replacement therapy, hemodialysis or peritoneal dialysis;
10. Asthma, allergic rhinitis or chronic obstructive lung disease
11. Psychiatric disease that is not well controlled (controlled defined as stable on a regimen for more than one year).
12. Pregnant or lactating women;
13. Having used Nitric Oxide Nasal Spray or participated in any other interventional drug clinical study within 30 days prior to first dose of study drug.
14. Subjects who have received hydroxychloroquine within 7 days before screening or subjects who require hydrochloroquine treatment.
15. Subjects who have received intranasal medication/treatment within 7 days before screening or subjects who require use of any intranasal medication
16. Subjects who have received medications with antiviral effect such as remdesivir, favipiravir, oseltamivir, ivermectin or inhaled corticosteroids within 7 days before screening or subjects who require use of any of these medications
17. Subjects using nitric oxide donor agents such as prilocaine, sodium nitroprusside and nitroglycerine

8.3. Subject Withdrawal Criteria

A subject may voluntarily discontinue study participation at any time after giving informed consent and before the completion of the last visit of the study. Subjects may also be withdrawn from study drug treatment at the discretion of the Investigator or Sponsor for safety, noncompliance, or administrative reasons. The Investigator may also discontinue the subject’s study participation at any time at his/her discretion and for any reason.

The reasons for subject withdrawal will be recorded and may include, but are not limited to:

1. Withdrawal of consent by the subject to continue in the study. If consent is withdrawn, the subject will not receive any further investigational product or further study observation.
2. Development of a serious or intolerable adverse event (AE) that necessitates discontinuation at the discretion of the Investigator (the AE section of the CRF/electronic case report form (eCRF) must be completed; AE includes serious adverse event (SAE) and death).
3. At the discretion of the Investigator, when he/she believes continued participation is not in the best interest of the subject.
4. Subjects with negative RT-PCR for SARS-CoV-2 at screening (Day 1) (swab collected before randomization). During screening (Day 1), swab from both sides in the nose will be collected for Rapid Antigen Test and RT-PCR. Subjects will be randomized based on

positive Rapid Antigen Test without waiting for RT-PCR report. If screening (Day 1) RT-PCR report is negative for SARS-CoV-2 or positive RT-PCR report is not available, the subject will be withdrawn from the study.

5. Subjects with disease progression to moderate or severe COVID-19, including but not limited to, requirement of high flow oxygenation, $SpO_2 \leq 94$, or arterial oxygen partial pressure (PaO_2)/ fraction of inspired O_2 (FiO_2) ≤ 300 mmHg, or respiratory rate of ≥ 24 breaths/min.
6. Subjects requiring admission to hospital for treatment or intensive care unit, or subjects requiring invasive or non-invasive ventilator support, ECMO or shock requiring vasopressor support.
7. Development of pneumonia based on x-ray or CT scan
8. Severe liver disease: underlying liver cirrhosis or alanine aminotransferase (ALT)/aspartate aminotransferase (AST) elevated over 5 times the ULN
9. At the discretion of the Investigator, when the subject does not adhere to the study procedures.
10. A protocol deviation that, in the opinion of the Sponsor and Investigator, warrants discontinuation from the study.
11. The Investigator can withdraw the subject from the study if the subject suffers from significant inter-current illness during the course of the study.
12. Positive pregnancy test at any time in the study.

Subjects who are withdrawn from the study should undergo early withdrawal visit and follow-up visit as explained in section 13.4 Early Withdrawal Visit.

8.3.1. Lost to Follow-up

A subject will be considered lost-to-follow-up only if no contact has been established by the time the study is completed such that there is insufficient information to determine the subject’s status. Subjects refusing to return to the site or to continue participation in the study should be documented as “withdrawal of consent” rather than “lost to follow-up.” Investigators should document attempts to re-establish contact with missing subjects throughout the study period. If contact with a missing subject is re-established, the subject should not be considered lost-to follow-up and any evaluations should resume according to the protocol.

8.3.2. Permanent Discontinuation of Study Drug

A subject who is permanently discontinued from further receipt of study drug, regardless of the reason (withdrawal of consent, due to an AE, other), will be identified as having permanently discontinued treatment.

8.3.3. Replacement of Subjects

Subjects, who are discontinued for any reason on or before Day 4 of treatment period, will be replaced by additional subjects, in order to achieve evaluable patients as per protocol, if required. Additional subjects will receive new subject IDs, randomization numbers and new treatment

allocation. Additional subjects will be randomly assigned to any of the two study treatments based on the randomization scheme, in the same way as other subjects. Subjects, who are discontinued after Day 4 of treatment period, will not be replaced.

9. TREATMENT OF SUBJECTS

9.1. Description of Study Drug

The following study drugs will be supplied by Glenmark for the study:

Table 3: Study Drugs

All subjects will receive standard supportive care in addition to the study drugs	
	**Investigational Product**
**Product Name**	Nitric oxide nasal spray
**Dosage Form**	Nasal Spray
**Dosage**	Two sprays each nostril.
**Dosage Frequency**	6 times daily. Study medication should be administered upon awakening (Dose 1), then administer additional doses (Dose 2 to 6) approximately every 2-3 hours while awake. Doses should be separated by at least 1.5 hours. Preferably, administer the last dose of the day (Dose 6) at bedtime.
**Route of Administration**	Nasal Spray
**Manufacturer**	SaNOtize At Nextar Chempahrm Solutions Ltd. 13B Einstein St. Weizmann Science Park, Ness Ziona 74140 Israel.
**Storage**	Store below 30°C, Do not Freeze. Discard Nasal Spray on the expiration date printed on the sprayer or 30 days after first opening, whichever comes first.
	**Comparator**
**Product Name**	Placebo of Nitric Oxide Nasal Spray
**Dosage Form**	Nasal Spray
**Dosage**	Two sprays each nostril.
**Dosage Frequency**	6 times daily. Study medication should be administered upon awakening (Dose 1), then administer additional doses (Dose 2 to 6) approximately every 2-3 hours while awake. Doses should be separated by at least 1.5 hours. Preferably, administer the last dose of the day (Dose 6) at bedtime.
**Route of Administration**	Nasal Spray

**Manufacturer**	SaNOtize At Nextar Chempahrm Solutions Ltd. 13B Einstein St. Weizmann Science Park, Ness Ziona 74140 Israel.
**Storage**	Store below 30°C, Do not Freeze. Discard Nasal Spray on the expiration date printed on the sprayer or 30 days after first opening, whichever comes first.

See Section 10 for additional information on study drug supplies.

9.2. Concomitant Medications

9.2.1. Prior Medications

Any medication (including over-the-counter medications) or therapy administered to the subject during the study (starting at or after the time of informed consent) will be recorded on the Prior and Concomitant Medication eCRF Page or Concomitant Non-drug treatment eCRF Page. The Investigator will record on the Adverse Event eCRF Page any AE for which the concomitant medication/therapy was administered. If the concomitant medication/therapy is being administered for a medical condition present at the time of entry into the study (onset before the time of consent), the Investigator will record the medical condition on the Medical History eCRF Page.

9.2.2. Permitted Concomitant Medications

Standard supportive Care will be permitted as per the contemporary Indian national guidelines on the treatment of mild COVID-19. According to the current guidelines from the Govt. of India, Ministry of Health and Family Welfare (Revised Guidelines, MoHFW, GoI 28 April 2021 and MoHFW, GoI 22 April 2021), recommended treatment of mild COVID-19 involves symptomatic treatment such as antipyretic for the treatment of fever and pain, anti-tussives for cough and adequate nutrition and appropriate hydration. These treatments will be permitted before and during the study.

Therapies allowed (not recommended) for mild COVID-19, by current guidelines based on low certainty of evidence, are Tablet Ivermectin or Tablet Hydroxychloroquine and if symptoms of fever and/or cough persist for more than 5 days, inhaled budesonide. However, as mentioned in the guidelines, the evidence of efficacy of these therapies is low subjects who require ivermectin, or hydroxychloroquine or inhaled budesonide treatment will not be included in the study and these medications will be prohibited during the study.

With the exception of medications listed under Excluded Concomitant Medications, subjects will be allowed continued use of prior medications that have been taken at stable doses, and other medications, at the discretion of the Investigator and in consultation with the Sponsor.

9.2.3. Prohibited Concomitant Medications

The following medications are prohibited throughout the entire study and for the indicated time prior to screening:

Prohibited concomitant medications	Time duration of prohibition:
• Hy droxychloroqine or chloroquine	Within 7 days before screening and during the study
• Drugs with anti-viral effects such as remdisivir, favipiravir, oseltamivir, ivermectin, inhaled or systemic corticosteroids and antiviral antibodies	Within 7 days before screening and during the study
• Nitric oxide donor agents such as prilocaine, sodium nitroprusside and nitroglycerine	Within 7 days before screening and during the study
Intranasal medications/treatment (all)	Within 7 days before screening and during the study

Use of prohibited medications during the study will be documented as protocol deviation. The decision about inclusion or exclusion of the data of these subjects from different analysis populations will be taken before database lock in the blinded data review meeting.

9.2.4. Lifestyle and/or Dietary Restrictions

Subjects will be instructed to follow the norms laid down by local and national government for COVID-19.

9.3. Treatment Compliance

Records of treatment compliance for each subject will be kept during the study. Clinical research associates (CRAs) will review treatment compliance during remote / on site monitoring visits and at the completion of the study.

The administration of study drugs (including investigational products) will be recorded in the appropriate sections of the eCRF. Site need to keep accountability of all used and unused investigational product.

Non-compliance with study drug:

If subjects do not take any dose of the study drug before swab from both sides in the nose on the day of Day 2, 4, or 8 visit it will be recorded as separate protocol deviation.

Subjects who use ≤ 80 % of the total expected doses of study drug from Day 2 to last day of treatment period (eg. 2 sprays each nostril 6 times per day * 7 days (day 1 visit to day 8 visit) = 42 expected doses for completed subjects) will be considered as non-compliant with study drug. Using similar steps, compliance will be calculated for subjects who are withdrawn early and will also be calculated up to RT-PCR negative status, separately. Non-compliance with study drug will be captured as protocol deviation.

First dose of study drug will be administered at the site on the day of randomization. As randomization and day 8 visit may happen at any time of the day, expected doses of study drug on

day 1 and day 8 may be different for different subjects. The compliance calculation is based on total number of doses from first dose on day 1 to last dose on visit 4.

See Section 10 for additional information on study drug supplies.

9.4. Randomization and Blinding

Subjects will be assigned to treatments based on a computer-generated randomization scheme that will be generated by an independent statistician. Dummy randomization scheme will be reviewed and approved by Glenmark statistician. Based on dummy approved randomization scheme, live randomization scheme will be generated by independent statistician. The randomization scheme and identification for each subject will be included in the final clinical study report for this study.

Eligible subjects will be assigned with a unique randomization code at randomization visit. If a subject withdraws from participation in the study, his/her enrolment/randomization code will not be reused. Randomization codes will be assigned strictly sequentially as subjects become eligible for randomization, including new subjects additionally randomized as replacement of the early withdrawn subjects (withdrawn within 4 days of treatment period).

To achieve blinding of the treatment allocation, a matching and identical placebo of the nitric oxide nasal spray (both containers) will be used. Treatment kits will be identified using a numeric code and treatment allocation will be based on kit numbers.

9.4.1. Un-blinding

Treatment allocation will not be un-blinded except for situations mentioned below:

Medical Emergencies:

In medical emergencies subject specific un-blinding can be performed when knowledge of the treatment allocation is required for appropriate management of the subject. The investigator will document and report the action to Glenmark without revealing the treatment allocation of the subject to the Glenmark staff.

SAEs requiring regulatory reporting of treatment allocation:

Un-blinding of the treatment allocation will be performed, of the subject with SAE that results in death, or SAE that is unexpected and suspected to be causally related to the investigational product that requires reporting of treatment allocation to the regulatory authorities. Nominated independent Glenmark person will be responsible for un-blinding.

Analysis of data:

Un-blinding will be performed for the planned analyses of data only after all decisions on the eligibility of the data of each subject have been made and documented. Such un-blinding will be performed when all the subjects complete the study for final analysis or when subjects allocated for interim analysis complete the study for interim analysis. Un-blinding for study will be performed at the time of planned analysis keeping the investigator site and subject blinded for ongoing subjects.

Request from Data Monitoring Committee or Data Safety Monitoring Board (DMC/DSMB): Un-blinding of treatment allocation of specific subjects may be performed, if requested by the DMC.

9.5. Subject Completion

Treatment duration is 7 days (up to day 8 visit) and the total study duration will be maximum for 18 days (up to day 19 visit) from randomization, which includes the follow-up period of maximum 11 days after the last dose of study drug. Subjects, who complete the 7 days treatment period, will be considered to have completed treatment period. For an individual subject, study participation will end at visit 4, if the subject’s baseline COVID-19 symptoms have resolved and subject is RT-PCR negative at visit 4. No further visits (including visit 5) will be conducted for such subjects. Subjects whose baseline COVID-19 symptoms are present and/or whose RT-PCR is positive at visit 4 will be followed up till maximum day 19 (±2 days) when visit 5 (end of study visit) will be conducted. However, if such subjects achieve status of COVID-19 symptoms resolved and RT-PCR negative any time between visit 4 and day 19, visit 5 can be conducted on such day (between visit 4 and day 19) and the subject’s study participation will end on that day.

10. STUDY DRUG MATERIALS AND MANAGEMENT

10.1. Study Drug

Nitric oxide is a colorless gas with sharp sweet odour.

10.1.1. Investigational Product

Investigational product: Nitric Oxide supplied as Nasal spray

Chemical Name and Structural Formula of Nitric Oxide

Chemical name: Nitric Oxide

Structural Formula:

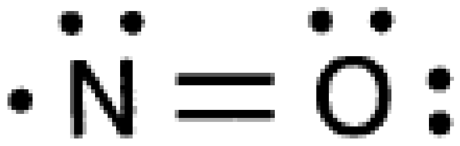

Molecular Formula: NO

Molecular Weight: 30.006 g/mol

Description: Nitric oxide appears as a colorless gas. Noncombustible but accelerates the burning of combustible material. Vapors heavier than air.

Investigational Products [active as well as Placebo] will be manufactured by SaNOtize Research and Development Corp. 25th Floor, 700 West Georgia Street, Vancouver, BC, Canada [Mfg Site at Nextar Chempahrm Solutions Ltd. 13B Einstein St. Weizmann Science Park, Ness Ziona 74140 Israel]

10.1.2. Placebo

Placebo to match nitric oxide nasal spray will be supplied

10.2. Study Drug Packaging and Labeling

The Investigational Product will be packed separately. Each pack will contain appropriate quantity of the study drug Nitric oxide nasal spray or matching placebo. Each subject kit will comprise of sufficient study drug and/or placebo for the entire study duration as planned.

Subjects randomized to any of the treatment group will be allocated individual subject pack according to the randomization number. Individual subject pack containers will be dispensed to subjects once they are randomized as per study visit.

The individual subject pack will be labelled with at least the following information:

1. Protocol Number
2. Name of the Test and Placebo Drug
3. Pharmaceutical dosage form, Route of Administration, Quantity of dosage units

4. Instructions for Administration of the Medication
5. Trial subject identification number/treatment number and where relevant, the visit number
6. "For Clinical Trial Use Only"
7. Storage Conditions
8. "Keep out of reach of children"
9. Batch Number and Expiry Date
10. Details of Sponsor and Investigator

The Study Monitor should be notified immediately of details of any supplies, which are inadvertently damaged. Details of any supplies, which are inadvertently damaged or unaccountable, for any reason, will be documented on Drug Accountability log that will be collected by the Study monitor at the end of the Study.

At the end of the Study, it must be possible to reconcile delivery records with those of used and unused stocks. Any discrepancy noted must be appropriately documented. The subject would be asked to fill in the subject diary every day, record dose and time of using IP, and the completed diary should be returned to the investigator site. The subject diary will be kept in the Study File.

Procedure for Handling of Used and Unused Investigational Product

All study drugs, including Test and placebo study drugs will be provided by Glenmark Pharmaceuticals Limited. The dispensing of the Investigational Drug will be done at the randomization visit for every subject and accountability check will be performed and recorded at every treatment visit. Used containers returned with remaining drug by the subjects will be collected at visit 4. At any time, the figures of supplied, used and remaining investigational product should match. The remaining Investigational Product both unused and used containers will be returned to Glenmark Pharmaceuticals Limited/IP Depot assigned by Glenmark Pharmaceuticals Limited at the end of the trial, in accordance with the study monitor's instructions. In this study, kit level reconciliation to be done as Nasal spray cannot be quantified after use by subjects.

10.3. Study Drug Storage

Investigational product (IP) should be stored below 30°C. Do not freeze. Nasal Spray should be discarded on the expiration date printed on the sprayer or 30 days after first opening, whichever comes first. Before use of Nitric Oxide Nasal Spray for the first time, the sprayer should be primed by squirting it away from the body into the air a few times until a consistent mist is generated. The Principal Investigator (or designee) is responsible for IP accountability at the site and its documentation. The Principal Investigator must also ensure that the dispensing and recording of IP is done only by authorized personnel. The IP records must be readily available for inspection by the study monitor and/or auditor/regulatory agency personnel.

10.4. Study Drug Preparation

Priming: Before use of Nitric Oxide Nasal Spray for the first time, the sprayer should be primed by squirting it away from the body into the air for 8 times until a consistent mist is generated.

10.5. Administration

On Day 1 of treatment, the subjects will be trained on the nasal spray administration. After priming, first dose of study drug will be self-administered under supervision. Standard supportive care will be provided to all the subjects during the study.

Following instructions will be given to the subjects;

- self-administer 2 sprays from the container, in each nostril
- self-administer the study drug on Day 2, 4, and 8.
- Self-administer the study drug 6 times daily (roughly every 2-3 hours when not asleep). Study medication should be administered upon awakening (Dose 1), then administer additional doses (Dose 2 to 6) approximately every 2-3 hours while awake. Doses should be separated by at least 1.5 hours. Preferably, administer the last dose of the day (Dose 6) at bedtime.
- Study drug should be used only from Day 1 to Day 8 (last dose should be taken at the site at visit 4). Subjects will continue to receive study treatment till Day 8, irrespective of virological or clinical cure status.
- Bring back the study medication containers to site whenever site (clinic) visit is conducted
- Subjects will be given 2 bottles of study drug. Subject has to open only one bottle at a time. 1 bottle will be opened at visit 1 (randomization) and the second bottle should be opened at Visit 3 (Day 4), under supervision of the site personnel.

Detailed dosing procedure will be mentioned in Pharmacy Manual of the study. Subjects will be trained on the storage requirements of the study drug.

10.6. Study Drug Accountability

The Investigator (or designee) is responsible for study drug accountability and its documentation at the site. The Investigator must also ensure that the dispensing and recording of study drug is done only by authorized personnel. The study drug records must be readily available for inspection by the Study Monitor and/or auditor/regulatory agency personnel. Upon completion of the study, copies of ‘study drug accountability records’ will be returned to the Sponsor or its designee. Refer to the Investigational Product Manual or other written instructions provided by the Sponsor or its designee for contact information and specific shipping and return.

10.7. Study Drug Handling and Disposal

No study drug (used or unused) can be returned to the Sponsor or disposed of at the investigational site until the Sponsor’s Study Monitor has verified/reconciled the accuracy of the study medication records at the site or through remote monitoring and indicated whether, how and where the medication should be destroyed. The Study Monitor must indicate the name and address of the individual to whom the returned materials should be shipped, if applicable.

11. ASSESSMENT OF EFFICACY

Efficacy will be assessed based on viral load parameters measured by the quantitative RT-PCR and clinical condition of the subject.

11.1. Quantitative RT-PCR:

Quantitative RT-PCR assessment will be based on the swab from both sides in the noses collected on Day 1, 2, 3, 4, and 8. Detailed procedure for collection of sample by swab from both sides in the nose and quantitative RT-PCR assessment will be provided separately.

11.2. Qualitative RT-PCR:

In addition to quantitative RT-PCR, qualitative RT-PCR will be conducted based on the swabs from nose collected on Day 1, 2, 3, 4 and 8 and if required on Day 19. Subjects whose RT-PCR is negative at Laboratory Visit – Day 3 or visit 4 (Day 8) will not undergo any further RT-PCR assessment. Subjects whose RT-PCR is positive at visit 4 (Day 8) will undergo RT-PCR assessment on Day 19. After Day 8 visit, additional qualitative RT-PCR tests can be conducted as unscheduled investigations in subjects who are RT-PCR positive on Day 8. The detailed procedure for collection of sample by swab from both sides in the nose and qualitative RT-PCR assessment will be provided separately. A single negative RT-PCR will be considered as negative conversion of SARS-CoV-2 RT-PCR.

11.3. WHO Progression Scale:

Change from baseline in subject's clinical status will be evaluated using the World Health Organization's Clinical Progression Scale (WHO Working Group, Lancet Infect Dis 2020). It is an 11-point scale ranging from a score of 0 to 10. Scores on the scale will be provided by the investigator on Day 1, 2, 4, 8 and visit 5. Note: Symptomatic subjects not requiring any assistance in the form of any medications and not requiring help from others for conducting daily activities will be considered as a score of 2, symptomatic independent. Only symptomatic subjects requiring external help for daily activities or requiring medications for symptom control will be considered to have a score of 3, symptomatic assistance needed.

The table below represents WHO Progression scale.

Patient State	Descriptor	Score
Uninfected	Uninfected; no viral RNA detected	0
Ambulatory mild disease	Asymptomatic; viral RNA detected	1
	Symptomatic; independent	2
	Symptomatic; assistance needed	3
Hospitalized: moderate disease	Hospitalized; no oxygen therapy*	4
	Hospitalized; oxygen by mask or nasal prongs	5
Hospitalized: severe diseases	Hospitalized; oxygen by NIV or high flow	6
	Intubation and mechanical ventilation, $pO_2/FiO_2 \geq 150$ or $SpO_2/FiO_2 \geq 200$	7
	Mechanical ventilation pO2/FIO_2 < 150 (SpO_2/FiO_2 < 200) or vasopressors	8
	Mechanical ventilation $pO_2/FiO_2 < 150$ and vasopressors, dialysis, or ECMO	9
Dead	Dead	10

ECMO=extracorporeal membrane oxygenation. FiO2=fraction of inspired oxygen. NIV=non-invasive ventilation. pO_2=partial pressure of oxygen. SpO_2=oxygen saturation. *If hospitalized for isolation only, record status as for ambulatory patient.
Source: Appendix 3

11.4. COVID-19 Related Symptoms Score:

COVID-19 Related Symptoms Score Diary Score is a patient reported outcome (PRO) symptom based questionnaire that consists of COVID-19 symptoms: stuffy/runny nose, sore throat, shortness of breath or difficulty breathing, cough, low energy or tiredness, muscle or body aches, headache, chills or shivering, feeling hot or feverish, nausea or feeling like you wanted to throw up, (US-FDA Guidance for Industry, September 2020: Assessing COVID 19 Related Symptoms in Outpatient Subjects in Clinical Trials of Drugs and Biological Products for COVID-19). These symptoms are scored on a scale from 0 to 3 (0=absent, 1=mild, 2=moderate, 3=severe). Questions how many times did you vomit (throw up) and how many times did you have diarrhea (loose or watery stools) will be captured as actual number of times. Questions sense of smell and sense of taste will be captured as same, less or lost.

The COVID-19 related symptom will be collected using the Subject Diary. Subjects will record their symptom score in the morning and evening, each day from Day 1 to Day 8 morning. Score on Day 1 will be obtained before the administration of the study drug and will be considered as the baseline score.

Spray Bottle Use Perception Questionnaire:

Spray bottle ‘experiences’ questions: These questions will be answered by the subject on Day 1, Day 2 and Day 8. The four-point Likert rating response options should be coded as -3, -1, 1, and 3 (most negative to most positive).

The subjects will be asked to answer the following questions about their experience with using the spray bottle (strongly disagree to strongly agree);

1. The spray bottle was easy to operate? Strongly Disagree; Disagree; Agree; Strongly Agree
2. The tip of the spray bottle in the nose was comfortable? Strongly Disagree; Disagree; Agree; Strongly Agree
3. The smell of the spray was mild to none? Strongly Disagree; Disagree; Agree; Strongly Agree
4. The aftertaste of the spray was mild to none? Strongly Disagree; Disagree; Agree; Strongly Agree
5. The spray mist was gentle? Strongly Disagree; Disagree; Agree; Strongly Agree
6. The nasal irritation was minimal to none? Strongly Disagree; Disagree; Agree; Strongly Agree
7. The amount of spray that leaked out of the nose was minimal to none? Strongly Disagree; Disagree; Agree; Strongly Agree

12. ASSESSMENT OF SAFETY

12.1. Safety Parameters

Safety assessments will consist of monitoring and recording all adverse events (AEs) and serious adverse events (SAEs), clinical laboratory measurements, vital signs, electrocardiograms, and physical examinations.

12.1.1. Demographic/Medical History

Subject demography information will be collected at the Screening. Demography information includes date of birth (or age), sex, race etc.

Medical History and Physical Examinations:

Medical and surgical history and current medical conditions will be recorded at the Screening. All relevant medical and surgical history within 6 months must be noted in the Medical History eCRF.

Glucose 6-phosphate dehydrogenase (G6DP) deficiency: Investigators should take history of known G6PD deficiency. For patients with known G6PD deficiency, based on investigator's opinion the subject can be continued or excluded or withdrawn from the study. G6PD deficiency can induce methemoglobinemia by inhibiting NADPH-flavine reductase, which prevents the reduction of methemoglobin. Furthermore, viral infections, including SARS-CoV-2 infection can precipitate methemoglobinemia and affect SpO2 measurements (Palmer K et al, Emerg Infect Dis. 2020). However, methemoglobinemia has been reported with the use of much higher doses of NO (80 ppm) in neonates, and is not expected with nasal spray as the dose of NO is much smaller (0.1 ppm). Also, in a phase 1 study of nitric oxide releasing solution much higher doses of NO were used without any change in methemoglobin levels. In a study conducted by Moni M et al in Indian subjects with COVID-19, use of >20 ppm dose of nitric oxide gas did not result in methemoglobinemia (Moni M et al, MedRxiv 2021).

12.1.2. Vital Signs

Vital sign measurements (i.e., systolic and diastolic blood pressure, respiratory rate, body temperature and pulse) will be obtained as designated on the Schedule of Assessments (Table 2).

On day 1, pulse measurement will be taken after the subject has been sitting and resting for at least 5 minutes and before blood samples are taken. BP readings should be taken while the subject is in a comfortable position with the arm supported at the level of the heart. Ideally, seated BP should be measured of the same arm, with the same machine. A standard mercury sphygmomanometer with a standardized cuff adapted to the size of the subject's arm is recommended.

Temperature should be preferably measured using a mercury thermometer or digital thermometer. However, measurement of temperature using a non-contact thermometer will be allowed. Oxygen saturation (SpO_2) will be measured on a fingertip.

12.1.3. Weight and Height

Height can be collected at the screening and weight according to the Schedule of Assessments (Table 2), where weight is measured in kg and height in centimeters.

12.1.4. Physical Examination

Physical examinations (comprehensive or symptom directed) will be performed as designated on the Schedule of Assessments (Table 2). Documentation of the physical examination will be included in the source documentation at the site. Significant findings at the Screening and before randomization will be recorded on the Medical History eCRF Page.

A complete physical examination should include general appearance, head, neck, face, eyes, ears, nose, throat, cardiovascular system, respiratory system, gastro-intestinal system, genito-urinary system, lymph nodes, nervous system, skin, and musculoskeletal system.

Baseline data will be collected at the Baseline (Screening and Randomization visit, visit 1), and new findings discovered on subsequent physical examinations should be recorded as changes from baseline. Changes from baseline physical examination findings that meet the definition of an AE will be recorded on the Adverse Events eCRF Page.

12.1.5. Electrocardiogram

A 12-lead ECG will be taken at screening after the subject has been lying down resting. The ECG will be evaluated by the investigator and entered as ‘Normal’ or ‘Abnormal’ in the eCRF. For 12-Lead ECG, if any clinically significant abnormality is detected, an additional triplicate ECG will be recorded and final opinion on abnormality and clinical significance will be recorded based on the triplicate ECG. There should be a gap of at least 1 minute between any two of the triplicate ECGs. If the ECG is evaluated as “Abnormal” the investigator should document the specific abnormality and provide assessment related to clinical significance.

12.1.6. Laboratory Assessments

Clinical laboratory tests to be performed, including hematology, chemistry, and urinalysis, are summarized in Appendix 2.

Blood and urine collection for laboratory assessments should be done prior to randomization. Subjects should be in a seated or supine position during blood collection. The Schedule of Procedures and Assessments (Table 2) shows that blood for clinical laboratory tests and urine for urinalysis will be collected in the study.

A central or local laboratory may be used to measure laboratory parameters.

12.1.6.1. Hematology

Complete Blood Count (CBC) comprising of Hemoglobin, Hematocrit (PCV), Red Blood Cells (RBC), White Blood Cells (WBC), mean corpuscular volume (MCV), mean corpuscular hemoglobin concentration (MCHC), Platelets and Differential (percent and absolute) Blood Cell Counts (Neutrophils, Lymphocytes, Monocytes, Eosinophils and Basophils).

12.1.6.2. Blood Chemistry

Biochemistry comprising of Random Blood Glucose, plasma total protein, albumin, serum creatinine, bilirubin (total, direct, indirect), ALT, AST, ALP, GGT, blood urea nitrogen (BUN), triglycerides, cholesterol (total, LDL, HDL), lactate dehydrogenase (LDH), Na, Cl, magnesium,

phosphorus, potassium, calcium, uric acid, and C-reactive protein. C-reactive protein and lactate dehydrogenase (LDH) will be measured only at screening.

12.1.6.3. Urinalysis

Urinalysis comprising of Appearance, Specific Gravity, Color, pH, Protein, Glucose, Presence of Blood, Bilirubin, Urobilinogen, and Microscopic examination including WBC/high power field and RBC/high power field.

12.1.6.4. Pregnancy Screen/testing

A serum and urine pregnancy test will be done for women of childbearing potential at screening visit, while only urine pregnancy test will be performed at visit 4. A Serum β-HCG at screening will be done. In the event of suspected pregnancy during the study, the test should be repeated and if pregnant, the subject should be discontinued and followed up as per pregnancy report form provided.

Methemoglobin measurement:

Methemoglobin will be measured at investigator site using non-invasive method in a subset of patients, based on feasibility. In this subset, methemoglobin will be measured at visits 1, 2, 3 and 4, if the subject visits the investigator site. For subjects doing home based visits, methemoglobin will not be measured. The subset will not be pre-defined and will be based on the availability of equipment for measuring methemoglobin. As the dose of NO in NONS is very small, no effect is expected on methemoglobin levels.

12.2. Adverse and Serious Adverse Events

The Investigator or site staff will be responsible for detecting, documenting, and reporting events that meet the definition of an AE or SAE.

12.2.1. Definition of Adverse Events

12.2.1.1. Adverse Event

An AE is defined as any untoward medical occurrence in a subject administered study drug that does not necessarily have a causal relationship with the treatment. An AE can therefore, be any unfavorable and unintended sign (including an abnormal laboratory finding), symptom or disease (new or exacerbated) temporally associated with the use of the study drug, whether or not related to the study drug. An AE includes any event, regardless of the presumed causality between the event and the study drug.

Events that, while not necessarily meeting the definition of AEs, should be treated as such because they may be reportable to Regulatory Authorities according to AE reporting regulations, whether or not considered causally associated with investigational product, include the following:

- Study drug overdose, whether accidental or intentional
- Study drug abuse
- An event occurring from study drug withdrawal

- Any failure of expected pharmacological action
- Inadvertent or accidental study drug exposure (eg, product leaking or being spilled onto a subject or care-giver)
- Unexpected therapeutic or clinical benefit from the study drug
- Medication errors (ie, incorrect route of administration, incorrect dosage, use of incorrect product).

Events that do not meet the definition of an AE include:

- Medical or surgical procedure (eg, endoscopy, appendectomy); the condition that leads to the procedure is an AE
- Situations where an untoward medical occurrence did not occur (social and/or convenience admission to a hospital including for quarantine purposes or for the purposes of protocol assessments)
- Anticipated day-to-day fluctuations of pre-existing disease(s) or condition(s) present or detected at the start of the study that do not worsen
- The disease/disorder being studied or expected progression, signs, or symptoms of the disease/disorder being studied, unless more severe than expected for the subject's condition.

Note that significant worsening of symptoms (ie, requiring hospitalization) will be reported as an AE.

12.2.1.2. Assessment of Severity of Adverse Events

The severity of AEs will be classified according to the Common Terminology Criteria for Adverse Events, CTCAE v5.0 (Link to CTCAE V5.0: https://ctep.cancer.gov/protocolDevelopment/electronic_applications/docs/CTCAE_v5_Quick_Reference_8.5x11.pdf)

The criteria for assessing severity are different from those used for seriousness (see Section 12.2.1.3 for the definition of an SAE).

The Reference Safety Information for this study is the Investigator's Brochure section on Guidance for Investigators, Undesirable Effects or the Summary of Product Characteristics sections on Warnings, Precautions, Contraindications and Undesirable Effects, or other, as appropriate.

12.2.1.3. Serious Adverse Event

A SAE is any untoward medical occurrence that, at any dose:

- Results in death
- Is life-threatening
  - NOTE: The term 'life-threatening' in the definition of 'serious' refers to an event in which the subject was at risk of death at the time of the event. It does not refer to an event, which hypothetically might have caused death, if it were more severe.

- Requires hospitalization or prolongation of existing hospitalization
  - NOTE: In this study, subjects may be detained in the isolation facility for the purposes of quarantine or protocol assessments. This will not qualify as an adverse event or serious adverse event. However, if there is extension of such detention for treatment or medical observation (in hospital); or such detention is primarily for the purposes of treatment or medical observation, this will qualify as serious adverse event. In general, hospitalization signifies that the subject has been detained (usually involving at least an overnight stay) at the hospital or emergency ward for observation and/or treatment that would not have been appropriate in the physician's office or outpatient setting. Complications that occur during hospitalization are AEs. If a complication prolongs hospitalization or fulfills any other serious criteria, the event is serious. When in doubt as to whether "hospitalization" occurred or was necessary, the AE should be considered serious.
- Hospitalization for elective treatment of a pre-existing condition that did not worsen from baseline is not considered an AE.
- Results in disability/incapacity
  - NOTE: The term disability means a substantial disruption of a person's ability to conduct normal life functions. This definition is not intended to include experiences of relatively minor medical significance such as uncomplicated headache, nausea, vomiting, diarrhea, influenza, and accidental trauma (eg, sprained ankle) that may interfere or prevent everyday life functions but do not constitute a substantial disruption.
- Is a congenital anomaly/birth defect
- Medical or scientific judgment should be exercised in deciding whether reporting is appropriate in other situations, such as important medical events that may not be immediately life-threatening or result in death or hospitalization but may jeopardize the subject or may require medical or surgical intervention to prevent one of the other outcomes listed in the above definition. These should also be considered serious. Examples of such events are invasive or malignant cancers, intensive treatment in an emergency room or at home for allergic bronchospasm, blood dyscrasias or convulsions that do not result in hospitalization, or development of drug dependency or drug abuse.

The severity of AEs is classified according to the CTCAE v5.0 (https://ctep.cancer.gov/protocolDevelopment/electronic_applications/docs/CTCAE_v5_Quick_Reference_8.5x11.pdf).

Note: Detention or admission of subjects to a clinic facility, not for health reasons, but for the reasons of protocol assessments or quarantine, will not be considered as Serious Adverse Event or Adverse Event.

12.3. Relationship to Study Drug

The relationship of AEs to study medication is classified as follows:

- Not Related: A causal relationship between the study treatment and the AE is not a reasonable possibility
- Related: A causal relationship between the study treatment and the AE is a reasonable possibility

Items to be considered when assessing the relationship of an AE to the study treatment are:

- Temporal relationship of the onset of the event to the initiation of the study treatment
- The course of the event, especially the effect of discontinuation of study treatment or reintroduction of study treatment, as applicable
- Whether the event is known to be associated with the study treatment or with other similar treatments
- The presence of risk factors in the study subject known to increase the occurrence of the event
- The presence of non-study treatment-related factors that are known to be associated with the occurrence of the event.

For each AE, the Investigator should answer the following question with Yes or No:

- Was there a reasonable possibility (evidence) that the drug caused the AE?
  - A reasonable possibility means that there are facts (evidence) or arguments to suggest a causal relationship.
  - NOTE: For subjects that have not started receiving study medication, or run-in phase medications, the answer must be no.

12.4. Recording Adverse Events

12.4.1. Collection of Adverse Events

The Investigator or site staff is responsible for detecting, documenting and reporting events that meet the definition of an AE or SAE.

AEs will be collected from the time of signing the informed consent form (ICF) until the follow up contact.

SAEs will be collected over the same period of time as stated above for AEs. However, any SAEs assessed as related to study participation (eg, study drug, protocol mandated procedures, invasive tests, or change in existing therapy) or related to a concomitant medication that is a Glenmark product, will be recorded from the time a subject consents to participate in the study up to and including any follow up contact. All SAEs will be reported to the Sponsor within 24 hours, as indicated in Section 12.5.

The Investigator will enquire about the occurrence of AEs/SAEs at every visit throughout the study (including Follow-up and Early Withdrawal visits where applicable), by asking the following non-leading verbal question of the subject (or care-giver, where appropriate):

- “Have you had any medical problems since your last visit?”

All AEs not resolved by the end of the study or that have not resolved upon the subject's discontinuation in the study must be followed until the event resolves, the event stabilizes or the event returns to baseline if a baseline value is available.

12.4.2. Recording of Adverse Events

All AEs, regardless of the seriousness, severity or relationship to the study medication must be recorded on the AE eCRF.

Adverse events that meet the definition of a SAE must be reported on the SAE Form provided for this study.

Adverse events must be documented in clear, unambiguous medical language. Do not use abbreviations or acronyms.

For each AE record only the diagnosis, do not report the characteristic signs and symptoms of the diagnosis as additional AEs.

If a diagnosis is not available record each sign and symptom as an AE, when a diagnosis becomes available, update the AE eCRF, to record the relevant diagnosis only.

In general, abnormal findings at screening should be recorded in the subject's Medical History or in the Concurrent Conditions section in the eCRF. However if, in the Investigators opinion, the finding is clinically significant and represents a condition that was not present at signing of informed consent, then the finding must be reported as an AE.

12.5. Reporting Serious Adverse Events

Prompt notification of SAEs by the Investigator to Glenmark, respective ethics committee and regulatory agency is essential so that legal obligations and ethical responsibilities towards the safety of subjects are met.

Glenmark has a legal responsibility to notify both the local regulatory authority and other regulatory agencies about the safety of a product under clinical investigation. Glenmark will comply with country-specific regulatory requirements relating to safety reporting to the regulatory authority, IRB/IEC and Investigators.

All SAEs must be reported to the Sponsor, respective ethics committee and regulatory agency immediately or within 24 hours of the Investigator or their staff becoming aware of them. Reporting to Glenmark should be performed by recording as much information as is available at the time on the SAE Form and sending it to the contact information provided below:-

Fax: + 44 1923 251137

Email: GlobalClinicalSAE@glenmarkpharma.com

When further information becomes available, the SAE Form should be updated with the new information and reported immediately via the same contact information. Follow-up reports must be submitted to the Sponsor until the event resolves, the event stabilizes or the event returns to baseline if a baseline value is available.

Additional information will be requested by the Sponsor as necessary.

12.5.1. Pregnancy

The Investigator will attempt to collect pregnancy information on any female subject or female partner of a male study subject who becomes pregnant while participating in this study. The Investigator will record pregnancy information on the appropriate form and submit it to the Sponsor within 2 weeks of learning of the partner's pregnancy. The partner will also be followed to determine the outcome of the pregnancy. Information on the status of the mother and child will be forwarded to the Sponsor. Generally, follow-up will be no longer than 6 to 8 weeks following the estimated delivery date. Any premature termination of the pregnancy will be reported.

Any pregnancy that occurs during study participation must be reported to the Sponsor, using a clinical trial pregnancy form, immediately or within 24 hours of the Investigator learning of its occurrence. The report should contain as much information as possible and should be sent to:

Fax: + 44 1923 251137

Email: GlobalClinicalSAE@glenmarkpharma.com

When further information becomes available, the Pregnancy Report Form should be updated with all new information and reported immediately via the same contact information above. The Pregnancy must be followed up to determine outcome (including premature termination) and status of mother and child, and this information must be sent to the Sponsor as above. Pregnancy complications and elective terminations for medical reasons must be reported as an AE or SAE. Spontaneous abortions must be reported as an SAE.

Additional information will be requested by the Sponsor as necessary.

Any SAE occurring in association with a pregnancy brought to the Investigator's attention after the subject has completed the study and considered by the Investigator as possibly related to the study drug, must be promptly reported to the Sponsor.

13. TIMING OF STUDY ASSESSMENTS

Study procedures and assessments are summarized across all study visits within the Schedule of Assessments (Table 2).

13.1. Screening and Randomization: Visit 1 (Day 1)

Screening and randomization will be done on Visit 1 (Day 1) of the study. This is required to be clinic visit. Before performing any procedures or assessments, the nature of the study and the potential risks associated with the study will be explained to all subjects and written informed consent will be obtained. Once informed consent has been obtained, the following procedures and evaluations will be performed:

Procedures to be completed before randomization:

1. Assess inclusion/exclusion criteria
2. Demographic data
3. Medical and Surgical history

4. Prior and concomitant medication evaluation
5. COVID status of immediate contacts
6. Rapid Antigen Test for COVID-19 will be conducted as part of screening assessment and subjects with positive result for COVID-19 antigen will be included in the study. Subjects with negative result for COVID-19 antigen will be considered as screen failure.
7. Vital signs including SpO_2
8. Physical examination
9. Height and weight measurements
10. 12-lead ECG
11. Blood and urine sample collection for clinical laboratory assessments (hematology, biochemistry, urinalysis) and C-reactive protein
12. Blood sample collection for Serum pregnancy test.
13. Urine pregnancy test should be conducted before randomization and only patients with negative result should be randomized.
14. Chest X-ray
15. Swab from both sides in the nose for Quantitative and Qualitative RT PCR
16. Methemoglobin: will be measured before randomization in subset of patients only if it is clinic visit, and depending on the availability of equipment
17. Assessment of AEs/SAEs
18. WHO Progression Scale Review
19. Subject Diary Recording and dispensing: Baseline symptom scores of the subject must be obtained before randomization.
20. Training of the subject on the use of study Nasal Spray.

After screening procedures:

1. Randomization
2. **Priming:** Before starting to use Nitric Oxide Nasal Spray for the first time, the sprayer should be primed by squirting it away from the body into the air a few times until a consistent mist is generated.
3. Investigational product/study drug dispensing
4. Supervised self-administration of study drug
5. Methemoglobin: will be measured in subset of patients 5 ± 2 minutes after first dose of study medication only if it is clinic visit, and depending on the availability of equipment
6. Spray Bottle Use Perception Questionnaire, to be answered by the subject
7. Explanation of lifestyle restrictions with to following of the norms laid down by local and national government for COVID-19

On Day 1, at investigator's discretion, subjects will be detained in the isolation facility for 7 days (up to day 8 visit) or sent home. If subjects are sent home, visits 2 (Day 2) and 3 (Day 4) will be planned to be clinic visits or telephonic or video-conference visits. If subjects are sent home, a thermometer and oxygen saturation monitor will be provided to the subject for recording.

13.2. Treatment Visit: Visit 2 (Day 2) and Visit 3 (Day 4)

This should preferably be a clinic visit. However, at the discretion of the investigator, this visit can be performed as a clinic visit or home-based visit.

Following procedures should be completed, preferably in the sequence mentioned:

1. Prior and concomitant medication evaluation
2. Assessment of AEs/ SAEs
3. COVID status of immediate contacts
4. Spray Bottle Use Perception Questionnaire, to be answered by the subject (only at visit 2)
5. Vital signs including SpO_2. All vital sign assessments will be conducted if it is clinic visit. All vital sign assessments except blood pressure will be conducted if it is a home-based visit.
6. WHO progression Scale Review
7. Subject Diary Review
8. Methemoglobin: will be measured in subset of patients only if it is clinic visit, and depending on the availability of equipment
9. Drug and Diary accountability
10. Swab from both sides in the nose for Quantitative and Qualitative RT PCR. Preferably there should be a time difference of at least 1,5 hours and maximum 4 hours from the last dose of study medication and time of swab collection.
11. Chest X-ray/CT scan (x-ray/CT scan based on investigator's discretion at visit 3 only). Subjects who are unable to visit the investigator site can perform the chest x-ray/CT scan at nearby facility.

If visits 2 (Day 2) and 3 (Day 4) are performed as home-based visits, blood pressure measurement is not required. If RT-PCR is negative at visit 3 (Day 4), no further RT-PCR assessments will be conducted in the study.

Laboratory Visit, Day 3: Laboratory Visit will be conducted on Day 3 at the clinic laboratory or at home. This will not be a clinic visit. During this visit, swab will be collected from nose for qualitative and quantitative RT-PCR assessment and no other assessments are required.

13.3. End of Treatment (EOT) Visit: Visit: 4 (Day 8 + 1)

This will be a clinic visit.

Following procedures should be completed, preferably in the sequence mentioned:

1. Prior and concomitant medication evaluation
1. Assessment of AEs/ SAEs
2. COVID status of immediate contacts
3. Spray Bottle Use Perception Questionnaire, to be answered by the subject
4. Vital signs including SpO_2.
5. Physical examination
6. Blood and Urine sample collection for hematology, serum chemistry, urinalysis
7. Methemoglobin: will be measured in subset of patients only if it is clinic visit, and depending on the availability of equipment.
8. WHO progression Scale Review
9. Subject Diary Review
10. 12-Lead ECG
11. Urine Pregnancy test
12. Drug and Diary accountability
13. Swab from both sides in the nose(s) for Quantitative and Qualitative RT PCR. Preferably there should be a time difference of at least 1.5 hours and maximum 4 hours from the last dose of study medication and time of swab collection.

If any COVID-19 symptoms are present at visit 4, ask the subject to self-monitor the symptoms and record the date of resolution of each symptom. If during the follow-up period subject requires oxygen support, hospitalization, non-invasive ventilation, mechanical ventilation or extracorporeal membrane oxygenation, the subject or subject's relative should contact and inform the investigator. Instruct the subject accordingly.

Management of clinically important information regarding endpoint assessments:

The sites will make active efforts to collect data regarding the endpoint assessments for subjects who cannot visit the clinic between day 1 and day 8 and between day 8 and day 19. Sites should contact subject on at least 2 occasions between day 8 and day 19 and document the same for subjects who have COVID-19 symptoms or are RT-PCR positive on Day 8. Assessment of resolution of COVID-19 symptoms, conversion of RT-PCR to negative and clinical worsening must be carried out by the sites to accurately characterize the time of change of clinical status and take necessary steps.

13.4. Early Withdrawal Visit

1. Prior and concomitant medication evaluation
2. Assessment of AEs/ SAEs
3. COVID status of immediate contacts
4. Spray Bottle Use Perception Questionnaire, to be answered by the subject

5. Vital signs including SpO_2
6. Physical examination
7. Blood and Urine sample collection for hematology, serum chemistry, urinalysis
8. Methemoglobin: will be measured in subset of patients only if it is clinic visit, and depending on the availability of equipment.
9. 12-Lead ECG
10. Urine Pregnancy test
11. WHO progression Scale Review
12. Recording symptoms in Subject Diary and Review
13. Drug and Diary accountability
14. Swab from both sides in the nose for Qualitative RT PCR. Preferably there should be a time difference of at least 1.5 hours and maximum 4 hours from the last dose of study medication and time of swab collection.

If early withdrawal visit is performed on the day of scheduled visit, then both scheduled visit and early withdrawal visit assessments will be performed on the same day. Follow-up visit should be performed 11 ± 2 days after early withdrawal visit, if there is subject's consent for follow-up visit. Subject can consent for conduct of follow-up visit verbally, which should be documented in source notes. If subject does not consent for follow-up visit or is lost to follow up, follow-up visit after early withdrawal visit should not be conducted.

13.5. Post Treatment Follow-Up (Telephonic or Clinic Visit): Visit 5 (Day 19±2)

For subjects whose baseline COVID-19 symptoms have resolved and RT-PCR is negative by visit 4, visit 5 will not be conducted and visit 4, will be considered as end of study for such subjects. Visit 5 will only be conducted for patients whose baseline COVID-19 symptoms are present at visit 4 and/or whose RT-PCR is positive at visit 4. Visit 5 can be conducted any time between visit 4 and day 19, based on subject's status change to symptom free and RT-PCR negative. If there is no change in such status, visit 5 will be conducted on day 19 ± 2 days. This visit will be a Telephonic visit. However, based on investigator or patient discretion the visit can be conducted as clinic visit. If additional assessments are conducted at this visit, the results of these assessments can be recorded on unscheduled visit eCRF Page, at the discretion of the investigators. If these assessments are routine and not related to adverse events, it is not mandatory to record the findings in eCRF.

11. Assessment of AEs/SAE
12. Review COVID-19 symptoms – if any COVID-19 symptoms were present on visit 4, check if the symptoms have resolved and record date of resolution of each symptom.
13. Record date of hospitalization, first time use of high-flow supplemental oxygen/non-invasive ventilation/mechanical ventilation/ extracorporeal membrane oxygenation, if applicable.

14. WHO progression Scale Review
15. Swab from both sides in the nose collection for qualitative RT-PCR, only if RT-PCR is positive on Day 8, visit 4

14. STATISTICS

The statistical analysis will be coordinated by the responsible Glenmark biostatistician. A Statistical Analysis Plan (SAP) will be written to provide details of the analysis, along with specifications for tables, listings, and figures to be produced. The SAP will be finalized before the database lock at the latest. Any changes from the analyses planned in the SAP will be justified in the Clinical Study Report (CSR).

All analyses will be performed using SAS® Version 9.4 or above.

In general, all data will be summarized with descriptive statistics (number of subjects, mean, standard deviation, minimum, median and maximum) for continuous endpoints, and frequency and percentage for categorical endpoints.

14.1. Sample Size

Based on the data of phase 2 study of Nitric Oxide Nasal Spray in the United Kingdom (Clinical Study Report IRAS ID 287727 NONS COVID Study), assuming a treatment effect of 5.0 log viral load in the primary endpoint, with a standard deviation of 10.0. One hundred seventy two (172) evaluable subjects (86 subjects per arm) will provide a power of 90% with two-sided significance level of 5%. For the secondary endpoint (RT-PCR conversion), assuming RT-PCR conversion of 40% in the Placebo group by Day 8, 260 subjects (130 subjects in each arm) will provide a power of 90% at two-sided significance level of 5 %, to detect a treatment difference of 20% in proportion of subjects achieving RT-PCR conversion.

Assuming a dropout rate of 15 %, total **306 subjects** (approx. 153 subjects per arm) will be randomized.

14.2. Analysis Sets

Analysis of the primary endpoint will be conducted using the modified ITT (mITT) analysis set. In addition, a supportive analysis will be performed for the primary efficacy endpoint using the Per Protocol Set (PPS).

14.2.1.1. Per Protocol Set

The per protocol analysis set (PPS) will include all subjects who are randomized, received at least one dose of study medication, completed the study and do not have any major protocol deviations. Major protocol deviations will be discussed and decided at the blinded data review meeting (BDRM) meeting before database lock.

14.2.1.2. mITT analysis set

The mITT analysis set will include all randomised subjects who received at least one dose of study medication, who have a non-missing baseline measurement and at least one post-baseline efficacy measurement for primary efficacy variable.

14.2.1.3. Safety Analysis Set

The Safety analysis set (SAS) will include all subjects who are randomized and received at least one dose of study medication. All safety endpoints will use the safety analysis set unless otherwise specified.

14.3. Endpoints

14.3.1. Primary Endpoint

- Change from baseline in log viral load through Day 8

14.3.2. Secondary Endpoint(s)

- Proportion of subjects with negative conversion of SARS-CoV 2 RT PCR on Day 2, 3, 4, and 8.
- Determine effect of NO nasal spray on clinical recovery [Time Frame: 18 days]. Determine the time to clinical recovery in participants with COVID-19 by measuring the proportion of patients from enrollment to resolution of baseline flu-like symptoms.
- Proportion of subjects requiring hospitalization for the treatment of COVID 19 [Tim frame: 18 days]
- Proportion of patients achieving a 2 point worsening in WHO Progression scale on Day 2, 4, 8, and 19
- Time from randomization to first time use of high flow supplemental oxygen/non-invasive ventilation/mechanical ventilation/ extracorporeal membrane oxygenation.
- Change from baseline in COVID-19 related symptom score of participants with COVID- 19 at Day 2, 3, 4, 8, and 19
- Change from baseline in log Viral load at Day 2, 3, 4, and 8 [Time Frame: 7 days]
- Safety and tolerability of 6 day administration of NO nasal spray treatment over 18 days [Time Frame: 18 days]. Measure the tolerability of the NO nasal spray treatment as determined by number of adverse events, pain, discomfort or discontinuations of treatment.

14.4. Subject Disposition

The number and proportion of subjects who complete the study or discontinue the study prematurely along with the reason for discontinuation will be presented.

The number and proportion of subjects who are screened but did not continue into the treatment period will be presented, along with the reason for discontinuation.

The number and proportion of subjects in each analysis set will be presented by treatment group.

14.5. Demographic and Other Baseline Characteristics

Subject demography information will be collected at Screening. Demography information includes date of birth (or age), sex, race/ethnicity etc.

14.6. Efficacy Analyses

The primary endpoint will be analyzed in the mITT population as the primary analysis. PP set will be used as sensitivity analysis. The details of analysis methods for primary and secondary endpoints will be provided in the statistical analysis plan (SAP).

As the usefulness of antiviral treatment is high in patients with risk of disease progression, analysis of efficacy endpoints will also be conducted in high risk population, which will be defined as subjects with risk of disease progression, i.e. co-morbidity (diabetes, hypertension, cardiovascular disease, etc.)/age ≥45 years/non-vaccinated subjects.

Endpoints will be analyzed up to Day 8. If more than 2/3rd of randomized subjects become RT-PCR negative by Day 4, the primary analysis of quantitative RT-PCR will be up to Day 4, visit 3. The details of analysis will be provided in the statistical analysis plan (SAP).

14.7. Pharmacokinetic, Pharmacodynamic, Biomarker, and Pharmacogenomic/Pharmacogenetic Analyses

Not Applicable.

14.8. Safety Analyses

All safety analyses will be performed on the Safety Analysis Set.

14.8.1. Extent of Exposure

The duration of exposure to study treatment will be summarized by treatment group

14.8.2. Adverse Events

Adverse events will be coded using the Medical Dictionary for Regulatory Activities (MedDRA). Adverse events will be summarized by presenting, for each treatment group, the number and percentage of subjects having any AE, having an AE in each primary system organ class, and having each individual AE (preferred term). Summaries will also be presented for AEs by event severity and for study treatment-related AEs. Subjects will be counted only once for each preferred term, system organ class, and by the highest severity of an event.

14.8.3. Laboratory Values

Laboratory evaluations will be summarized with descriptive statistics at the days, and change from baseline summarized for each post-randomization. Laboratory measurements may also be summarized based on the number and percentage of subjects above or below a pre-specified threshold. All laboratory data will be displayed in listings.

14.8.4. Vital Signs

Descriptive statistics will be used to summarize vital sign results and changes from baseline by treatment group and time. All vital signs data will be displayed in listings.

14.8.5. Electrocardiograms

Descriptive statistics for ECG parameters and changes from baseline will be presented by treatment group [or treatment or dose, depending on the study design].

14.8.6. Other Safety Analyses

Not applicable

14.9. Other Analyses

Not applicable

14.10. Interim Analysis

Interim analysis will be performed when ~50 % of the subjects complete the study according to group sequential design and sample size will be re-assessed. Based on the pre specified analysis and as per Subject Expert Committee (SEC) recommendation dated 29/12/2021, to perform another analysis of current recruited subjects to include high risk population. The details will be described at the SAP.

14.11. Data Monitoring Committee or Data Safety Monitoring Board

An independent Data Monitoring Committee (DMC) or Data Safety Monitoring Board (DSMB) which will review safety/efficacy data will be formed for the review of blinded and un-blinded (when available) data to advise the Sponsor.

15. DIRECT ACCESS TO SOURCE DATA/DOCUMENTS

15.1. Study Monitoring

Before an investigational site can enter a subject into the study, a representative of the Sponsor will visit the investigational study site or remotely evaluate the site to:

- Determine the adequacy of the facilities
- Discuss with the Investigator and other personnel their responsibilities with regard to protocol adherence, and the responsibilities of the Sponsor or its representatives. This will be documented in a Clinical Study Agreement between the Sponsor and the Investigator.

During the study, a monitor from the Sponsor or representative will have regular contacts with the investigational site, for the following:

- Provide information and support to the Investigator
- Confirm that facilities remain acceptable
- Confirm that the investigational team is adhering to the protocol, that data are being accurately recorded in the eCRF, and that investigational product accountability checks are being performed
- Perform source data verification. This includes a comparison of the data in the eCRFs with the subject's medical records at the hospital or practice, and other records relevant to the study. This will require direct access to all original records for each subject (eg, clinic charts).
- Record and report any protocol deviations not previously sent to the Sponsor.
- Confirm AEs and SAEs have been properly documented on eCRFs and confirm any SAEs have been forwarded to the Sponsor and those SAEs that met criteria for reporting have been forwarded to the IRB.

The monitor will be available between visits if the Investigator or other staff needs information or advice.

15.2. Audits and Inspections

Authorized representatives of the Sponsor, a regulatory authority, an IEC or an IRB may visit the site to perform audits or inspections, including source data verification. The Investigator should contact the Sponsor immediately if contacted by a regulatory agency about an inspection.

15.2.1. Inspection

An inspection is defined as the act of a regulatory authority of conducting an official review of documents, facilities, records and any other resources that are deemed by the authorities to be related to the clinical study and that may be located at the site of the study, or at the Sponsor's and/or CRO facilities or any other establishments deemed appropriate by the regulatory authorities.

15.2.2. Audit

An audit is a systematic and independent review of study-related activities and documents to determine whether study-related activities were conducted and the data were accurately recorded and analyzed according to the protocol, SOPs, GCP, and the appropriate requirements.

In conducting this study the Investigator accepts that the Sponsor, IRB/IEC or regulatory body may, at any time by appointment, conduct an audit of the study site.

15.3. Institutional Review Board/Independent Ethics Committee

The Investigator must obtain IRB/IEC approval for the clinical study. Initial IRB/IEC approval, and all materials approved by the IRB/IEC for this study including the subject consent form and recruitment materials must be maintained by the Investigator and made available for inspection.

16. QUALITY CONTROL AND QUALITY ASSURANCE

To ensure compliance with Good Clinical Practices and all applicable regulatory requirements, The Sponsor may conduct a quality assurance audit. Please see Section 15.2 for more details regarding the audit process.

17. ETHICS

17.1. Ethics Review

The final study protocol, including the final version of the ICF, must be approved or given a favorable opinion in writing by an IRB or IEC as appropriate. The Investigator must submit written approval to the Sponsor before he or she can enroll any subject into the study.

The Investigator is responsible for informing the IRB or IEC of any amendment to the protocol in accordance with local requirements. In addition, the IRB or IEC must approve all advertising used to recruit subjects for the study, as well as any materials (eg diaries, questionnaires) to be given to subjects. The protocol must be re-approved by the IRB or IEC upon receipt of amendments and annually, as local regulations require.

The Investigator is also responsible for providing the IRB or IEC with reports of any reportable serious adverse drug reactions from any other study conducted with the investigational product. The Sponsor will provide this information to the Investigator.

Progress reports and notifications of serious adverse drug reactions will be provided to the IRB or IEC according to local regulations and guidelines.

17.2. Ethical Conduct of the Study

The study will be performed in accordance with ethical principles that have their origin in the Declaration of Helsinki and are consistent with ICH/GCP guidelines, applicable regulatory requirements and the Sponsor’s policy on Bioethics.

17.3. Written Informed Consent

The Investigator will ensure that the subject is given full and adequate oral and written information about the nature, purpose, possible risk and benefit of the study. Patients must also be notified that they are free to discontinue from the study at any time. The subject should be given the opportunity to ask questions and allowed time to consider the information provided.

The Investigator is responsible for obtaining informed consent from each subject/legally acceptable representative (LAR) participating in the study. All pertinent aspects of the study must be explained to the subject/LAR before he or she signs the informed consent. The subject’s signed and dated informed consent must be obtained before conducting any study procedures.

Informed consent must be obtained from the subject/LAR before any activity or treatment is undertaken which is not part of routine care. This includes, but is not limited to, the performance of diagnostic or therapeutic screening procedures and the administration of the first dose of the study medication. Each subject must be informed that participation in the study is voluntary, that he/she may withdraw from the study at any time, and that withdrawal of consent will not affect his/her subsequent medical treatment or relationship with the treating physician.

This informed consent should be given by means of a standard written statement, written in nontechnical language. The subject/LAR should understand the statement before signing and dating it and will be given a copy of the signed document.

The Investigator(s) must maintain the original, signed ICF. A copy of the signed ICF must be given to the subject.

18. DATA HANDLING AND RECORDKEEPING

18.1. Data Collection

As part of the responsibilities assumed by participating in the study, the Investigator agrees to maintain adequate and accurate case histories for the subjects treated under this protocol. Case histories include eCRFs and supporting data including, but not limited to, signed and dated informed consent forms, progress notes, hospital charts, nurse's notes, diary cards, laboratory reports, ECG strips, etc.

Subject demographics and key/essential disease baseline characteristics thought to affect outcome, ie, stratification variables and other prognostic factors will be collected, as available, for all subjects who provide written informed consent. For subjects who provide informed consent and were not assigned to treatment/randomized into the study, the reason the subject was not assigned to treatment/randomized, ie, did not meet one or more inclusion criteria, met one or more exclusion criteria, or other (eg, lost to follow-up, consent withdrawn), will also be collected.

For screen failure subjects, only the information related to consent, screen failure reasons and other minimal required information will be collected in the eCRF. Related details will be provided in the eCRF completion guidelines.

18.2. Inspection of Records

The Sponsor will be allowed to conduct site visits to the investigation facilities for the purpose of monitoring any aspect of the study. The Investigator agrees to allow the monitor to inspect the drug storage area, study drug stocks, drug accountability records, subject charts and study source documents, and other records relative to study conduct.

18.3. Retention of Records

The Investigator must maintain all documentation relating to the study for a period of 2 years after the last marketing application approval, or if not approved 2 years following the discontinuance of the test article for investigation. If it becomes necessary for the Sponsor or the Regulatory Authority to review any documentation relating to the study, the Investigator must permit access to such records.

18.4. Financing and Insurance

The Sponsor will provide clinical study insurance for any subjects participating in the study in accordance with all applicable laws and regulations.

19. PUBLICATION POLICY

The Sponsor recognizes and supports the publication and dissemination of scientific information as a means of furthering knowledge. The general strategy regarding publication of the study (what, when, where, etc.) will be mutually agreed upon by the Investigator and Sponsor. However, in order to protect its commercial interests, the Sponsor reserves the right to manage the publication of all study results. The Investigator agrees that oral and written communication to third parties of any procedures or results from the study is subject to prior written consent of the Sponsor. Presentation material and/or manuscript(s) for publication will be reviewed by Sponsor prior to submission for publication. This review will be completed within 30 days of receiving presentation material and 60 days of receiving the manuscript from the Investigator. Alterations in the material will only be made in agreement between the Investigator and the Sponsor.

In the event of inconsistency between the above and the study contract, the terms of the study contract would prevail to the extent of such inconsistency.

20. LIST OF REFERENCES

1. Akerstrom S, Gunalan V, Keng CT, Tan YJ, Mirazimi A. Dual effect of nitric oxide on SARS-CoV replication: viral RNA production and palmitoylation of the S protein are affected, Virology 395 (2009) 1–9.
2. Akerstrom S, Mousavi-Jazi M, Klingstrom J, Leijon M, Lundkvist A, Mirazimi A. Nitric Oxide Inhibits the Replication Cycle of Severe Acute Respiratory Syndrome Coronavirus. J Virology 2005; 79(3):1966-9.
3. Alhareth D, Badour S. The Potential Role of Inhaled Nitric Oxide in Managing COVID-19 Associated Lung Complications Review of Literature. Int J Respir Pulm Med 2020, 7:145.
4. Chen L, Liu P, Gao H, Sun B, Chao D, Wang F, Zhu Y, Hedenstierna G, Wang CG. Inhalation of Nitric Oxide in the Treatment of Severe Acute Respiratory Syndrome: A Rescue Trial in Beijing. Clin Infect Dis. 2004 Nov 15;39(10):1531-5.
5. Clinical Study Report of study Clinical Study Report IRAS ID 287727 NONS COVID Study; IRAS ID 287727 NONS COVID StudyEudraCT 2020-004994-27
6. Clinical Study Report of study COVID-CTP-01
7. Development Timelines, Drug Development and Delivery. Link: https://drug-dev.com/development-timelines-drug-development-times-what-it-takes-part-2/ . Last accessed: 26-Apr-2021.
8. EU Clinical Trial Register. EudraCT 2020-004994-27.
9. Fang W, Jiang J, Su L, Shu T, Liu H, Lai S, Ghiladi RA, Wang J. The role of NO in COVID-19 and potential therapeutic strategies. Free Radic Biol Med 163 (2021) 153-162.
10. Genosyl US Prescribing Information. Link: https://www.accessdata.fda.gov/drugsatfda_docs/label/2019/202860s000lbl.pdf . Last accessed: 27-Apr-21
11. Govt of India. Link: https://www.mygov.in/covid-19 . Last accessed: 07-May-2021.
12. Haagmans BL, Osterhaus AD. Coronaviruses and their therapy, Antivir. Res. 2006; 71:397–403.
13. Hermann E et al., Role of nitric oxide in the regulation of lymphocyte apoptosis and HIV-1 replication, Int. J. Immunopharm. 19 (1997) 387–397.
14. INOmax, Scientific Discussion, 2005: Available from: https://www.ema.europa.eu/en/documents/scientific-discussion/inomax-epar-scientific-discussion_en.pdf Last accessed: 09-May-2021
15. INOmax, Product Monograph, 2017: Available from: https://www.mallinckrodt.ca/wp-content/uploads/2017/11/INOMAX-Product-Monograph.pdf Last accessed : 09-May-2021

16. INOmax-USPI, 2019: Available from : https://www.accessdata.fda.gov/drugsatfda_docs/label/2019/020845s020lbl.pdf Last accessed 09-May-2021.

17. India TV News. Available from: https://www.indiatvnews.com/health/covid19-uk-clinical-trial-claims-breakthrough-treatment-of-sanotize-698971 Last accessed: 01-May-21

18. Letko M, Marzi A, Munster V. Functional assessment of cell entry and receptor usage for SARS-CoV-2 and other lineage B betacoronaviruses. Nat Microbiol 5 (2020) 562–569.

19. Mělkov´a Z, Esteban M. Inhibition of vaccinia virus DNA replication by inducible expression of nitric oxide synthase, J. Immunol. 155 (1995) 5711–5718.

20. MoHFW GoI. Ministry of Health and Family Welfare, Government of India. Clinical guidance for management of adult COVID-19 patients. 22 April 2021. Available from: https://www.icmr.gov.in/pdf/covid/techdoc/COVID19_Management_Algorithm_22042021_v1.pdf. Last accessed: 07 May 2021.

21. Moni M, Madathil T, Sathyapalan DT, Menon V, Gutjahr G, Edathadathil F, Sureshkumar D, Prasanna P, Jose S, Jerome R, Krishnan A, Pillai IC, Kumar G, Nair B, Jayant A. A Feasibility Trial to Evaluate the Composite Efficacy of Inhaled Nitric Oxide in the Treatment of Covid 19 Pneumonia : Impact on Viral Load and Clinical Outcomes. MedRxiv 2021. DOI: https://doi.org/10.1101/2021.04.15.21255300

22. Palmer K, Dick J, French W, Floro L, Ford M. Methemoglobinemia in Patient with G6PD Deficiency and SARS-CoV-2 Infection. Emerg Infect Dis. 2020;26(9):2279-2281. https://doi.org/10.3201/eid2609.202353

23. Reiss CS, Komatsu T. Does Nitric Oxide Play a Critical Role in Viral Infections? J Virol, June 1998, p. 4547–4551.

24. Revised Guidelines MoHFW. Ministry of Health and Family Welfare, Government of India, 28 April 2021. Available from: RevisedguidelinesforHomeIsolationofmildasymptomaticCOVID19cases. Last accessed:07 May 2021.

25. SaNOtize Data. SaNOtize Press Release. Available from: https://www.businesswire.com/news/home/20210315005197/en/UK-Clinical-Trial-Confirms-SaNOtize%E2%80%99s-Breakthrough-Treatment-for-COVID-19 . last accessed: 28-Apr-2021

26. Times of India. Available from: https://timesofindia.indiatimes.com/world/uk/anti-covid-nasal-spray-in-approval-queue-could-be-game-changer-for-india-scientist/articleshow/82102517.cms Last accessed: 28-Apr-21.

21. APPENDICES

APPENDIX 1. LIST OF CONTACT DETAILS

Additional information and contact details related to the study will be provided to each clinical site separately in relevant documents and procedural manuals.

SAE and Pregnancy Reporting:
Fax: + 44 1923 251137
Email: GlobalClinicalSAE@glenmarkpharma.com

Dr. Rahul Kodgule
General Manager | Clinical Development
Glenmark Pharmaceuticals
Andheri East, Mumbai- 400099
E-mail: Rahul.Kodgule@glenmarkpharma.com

Dr Monika Tandon
Senior Vice-President, Clinical Development
Glenmark Pharmaceutical Ltd.
Andheri (E), Mumbai-99
Phone: +91 22 4018 9847
Mobile: +91 9930557454
E-mail: Monika.Tandon@glenmarkpharma.com

Amol Pendse
Glenmark Research Centre, A-607,
MIDC Mahape, Navi Mumbai 400709
Phone: +91 22 6772 0000 (Extn) 3502
Mobile: +91 9167256463
E-mail: Amol.Pendse@glenmarkpharma.com

APPENDIX 2. CLINICAL LABORATORY TESTS

Table 4: Clinical Laboratory Tests

Panel	Test to be performed
Hematology	hemoglobin<br>hematocrit (PCV)<br>red blood cell (RBC) count<br>mean corpuscular volume (MCV)<br>mean corpuscular hemoglobin concentration (MCHC)<br>white blood cell (WBC) count with differential (absolute number and percentages) [neutrophils, lymphocytes, monocytes, eosinophils, and basophiles]<br>platelet count
Serum Chemistry	Random blood glucose<br>alanine transaminase (ALT)<br>albumin<br>alkaline phosphatase (ALP)<br>aspartate transaminase (AST)<br>bilirubin (total, direct and indirect)<br>blood urea nitrogen (BUN)<br>calcium (Ca)<br>chloride (Cl)<br>cholesterol (total, LDL, HDL)<br>C-reactive protein (only at screening)<br>creatinine<br>gamma-glutamyl transferase (GGT)<br>lactate dehydrogenase (LDH, only at screening)<br>magnesium<br>phosphorus<br>potassium (K)<br>sodium (Na)<br>plasma total protein<br>triglycerides<br>uric acid
Urinalysis	color<br>appearance<br>pH<br>specific gravity<br>presence of blood, glucose, protein, bilirubin and urobilinogen<br>microscopy including WBC/high power field (HPF), RBC/HPF

Table 4: Clinical Laboratory Tests (Continued)

Panel	Test to be performed
Pregnancy Testing	serum beta-human chorionic gonadotropin (serum β-hCG) (only at screening and for confirmation of pregnancy if urine pregnancy test is positive)<br>Urine Pregnancy Test (at site)
Other	Quantitative SARS CoV2 RT-PCR (swab from both sides in the nose) for efficacy assessment<br>Qualitative SARS CoV2 RT-PCR (swab from both sides in the nose) for efficacy assessment

APPENDIX 3. WHO PROGRESSION SCALE

A complete WHO Progression scale can be accessed from the below link:

https://www.who.int/docs/defaultsource/documents/emergencies/minimalcoreoutcomemeasure.pdf [Accessed on 06 May 2021]
