# Supplementary material for: SARS-CoV-2 accelerated clearance using a novel nitric oxide nasal spray (NONS) treatment: A randomized trial
Source: Lancet Reg Health Southeast Asia. 2022 Jun 29;3:100036. doi: 10.1016/j.lansea.2022.100036 (PMC9239922; doi:10.1016/j.lansea.2022.100036)
Supplement: Supplementary file 2 [file mmc2.docx]

**Supplementary Material**

**SARS-CoV-2 Accelerated Clearance using a Novel Nitric Oxide Nasal Spray (NONS) Treatment**

**Appendix**

**Table of Contents**

[Table S1. Protocol Amendment Summary 1](#_Toc105678252)

[Table S2. Study Procedures and Assessments Schedule 2](#_Toc105678253)

[Table S3. Computer-Generated Randomization Procedure 6](#_Toc105678254)

[Table S4. In Vitro Antiviral Activity of NONS and Placebo 6](#_Toc105678255)

[Table S5. Characteristics of the Patients at Baseline (mITT high risk population, mITT unvaccinated population and mITT population) 7](#_Toc105678256)

[Table S6. Summary of SARS-CoV-2 Viral RNA Values (log10 copies per mL) at Day 1 (Baseline), Day 2, Day 3, Day 4, and Day 8 (mITT High Risk Population) 8](#_Toc105678257)

[Table S7. Mean SARS-CoV-2 Viral Load (log10 copies per mL) Change from Baseline Through Day 8 in Adult COVID-19 Infected Patients (mITT Unvaccinated and mITT Populations: MMRM) 9](#_Toc105678258)

[Table S8. Mean SARS-CoV-2 Viral Load (log10 copies per mL) Change from Baseline Through Day 8 in Adult COVID-19 Infected Patients (High Risk Per Protocol Population [PP]: MMRM) 9](#_Toc105678259)

[Table S9. Mean SARS-CoV-2 Viral Load (log10 copies per mL) Change from Baseline Through Day 8 in Adult COVID-19 Infected Patients (mITT High Risk and mITT Populations: MMRM with vaccinated status added as an additional covariate) 10](#_Toc105678260)

[Table S10. Analysis of Time to Negative Conversion of SARS-CoV-2 RT-PCR (qualitative; mITT Unvaccinated and mITT Populations) 11](#_Toc105678261)

[Table S11. Proportion of COVID-19 Infected Patients with ≥ 2 Point Clinical Status Improvement with WHO Progression Scale Score (mITT Unvaccinated and mITT Population) 11](#_Toc105678262)

[Table S12. NONS Safety - Lack of Vasodilation Potential from Locally or Systemically Absorbed Nitric Oxide and Expected Impact on Nasal Immunity (Safety Analysis Population) 12](#_Toc105678263)

# **Table S1. Protocol Amendment Summary**

| **Original Protocol Version 3.0** (05 AUG 2021); used for the initial trial execution. Tabular information describes key changes made to the protocol. | |
| --- | --- |
| Protocol Version 4.0 (07 SEP 2021) | Laboratory: Additional RT-PCR assessment included at Day 3 (approximately 48 hours on treatment); allowing a more complete and early profiling of the change in viral RNA change from baseline. |
| Protocol Version 5.0 (04 OCT 2021) | Eligibility: Exclusion criteria for vaccinated subjects removed to allow inclusion of any vaccinated subject; reflecting current clinical practice. |
| Protocol Version 6.0 (31 DEC 2021) | Primary endpoint analysis: High risk patient population defined as population for analysis per recommendations of the regulatory agency (DCGI); reflecting the usefulness of antiviral treatment therapy in those at high risk of illness progression (unvaccinated, ≥ 45 years of age, or comorbidities of diabetes, hypertension, obesity, cardiovascular risk, or cerebrovascular risk disease). |

# **Table S2. Study Procedures and Assessments Schedule**

| Study Period | Treatment Period | | | | | Early Withdrawal Visita | Post-Treatment Follow-Up (Telephonic or clinic visit^f^) |
| --- | --- | --- | --- | --- | --- | --- | --- |
|  | Screening & Randomization |  | | | End of Treatment  **(EOT)** |  |  |
| Visit | 1 | 2 | Lab Visit –  Day 3 | 3 | 4 |  | 5 |
| Time Point (days) | 1 | 2 | 3 | 4 | 8+1 |  | 19±2 (or anytime between visit 4 and day 19) |
| Written informed consent | X |  |  |  |  |  |  |
| Demographics | X |  |  |  |  |  |  |
| Medical and surgical history | X |  |  |  |  |  |  |
| Review COVID-19 Symptoms and clinical status | X | X |  | X | X | X | X |
| Prior and concomitant medications | X | X |  | X | X | X | X |
| Physical examination | X |  |  |  | X | X |  |
| Height and Weight | X |  |  |  |  |  |  |
| Vital signs, including SpO_2_^b^ | X | X |  | X | X | X |  |
| 12-lead ECG^c^ | X |  |  |  | X | X |  |
| Hematology, serum chemistry, urinalysis | X |  |  |  | X | X |  |
| C-Reactive Protein | X |  |  |  |  |  |  |
| Chest X-ray^h^ | X |  |  | X |  |  |  |
| Methemoglobin^d^ | X | X |  | X | X | X |  |
| Quantitative RT PCR (swab from nose) | X | X | X | X | X | X |  |
| Qualitative RT PCR (swab from nose) | X | X | X | X | X | X | X^g^ |
| Review inclusion/exclusion criteria | X |  |  |  |  |  |  |
| Randomization | X |  |  |  |  |  |  |
| Pregnancy test^e^ | X |  |  |  | X | X |  |
| Assessment of AEs/SAEs | X | X |  | X | X | X | X |
| WHO Progression Scale Review | X | X |  | X | X | X | X |
| Spray Bottle Use Perception Questionnaire | X | X |  |  | X | X |  |
| Subject Diary dispensing | X |  |  |  |  |  |  |
| Subject Diary Review |  | X |  | X | X | X |  |
| Investigational product dispensing | X |  |  |  |  |  |  |
| Drug and Diary accountability |  | X |  | X | X | X |  |

AE = adverse event; ECG = electrocardiogram; SAE = serious adverse event.

Excluded participants: Included those with a current known pneumonia based on x-ray or computerized tomography (CT) scan or history of pneumonia within three months before screening; history of a hospitalization for the treatment of COVID-19; asthma, allergic rhinitis, or chronic obstructive lung disease; psychiatric disease that was not well controlled (on a stable therapy regimen for < one year) for an inability to accurately and appropriately self-medicated; and pregnant or lactating women for unnecessary fetal and new-born perceived risk. Also excluded were those with prolonged QT, defined as QTcF ≥ 450 milliseconds for men and QTcF ≥ 470 milliseconds for women; severely reduced left ventricular function (ejection fraction < 30%); severe renal impairment (creatinine clearance < 30 mL/min) or had received continuous renal replacement therapy, hemodialysis, or peritoneal dialysis.

Participants were not permitted to have used nitric oxide nasal spray or participated in any interventional drug study within the previous 30 days; received an antiviral (remdesivir, favipiravir, oseltamivir), received ivermectin, hydrochloroquine, inhaled corticosteroids or any intranasal therapy within seven days before screening or required the use of these medications, and those using nitric oxide donor agents (prilocaine, nitroglycerine, etc.).

Study medication (investigational drug products; active and placebo): Produced and supplied by SaNOtize Research and Development Corp. 25th Floor, 700 West Georgia Street, Vancouver, BC, Canada; manufactured at Nextar Chempharm Solutions Ltd. 13B Einstein St. Weizmann Science Park, Ness Ziona 74140, Israel.

^a^ Early withdrawal visit performed if applicable. If early withdrawal visit is performed on the day of scheduled visit, then both scheduled visit and early withdrawal visit assessments performed on the same day. Follow-up visit performed 11±2 days after early withdrawal visit if subject’s consent.

^b^ If visits 2 (Day 2) or 3 (Day 4) are performed as home-based visits, blood pressure measurements and physical examination not required

^c^  12-Lead ECG, if any clinically significant abnormality detected, an additional triplicate ECG recorded.

^d^ Methemoglobin assessment performed in only a subset of patients. In this subset, methemoglobin measured on Day 1 (before randomization and 5 minutes after first dose of study medication), and Days 2, 4 and 8.

^e^ Female subjects only. Urine and serum pregnancy test performed at Screening; urine pregnancy test performed at visit 4 and early withdrawal visit.

^f^ Visit 5 only conducted for patients whose baseline COVID-19 symptoms are present at visit 4 and/or whose RT-PCR is positive at visit 4. For subjects who were RT-PCR* positive at visit 4, a nasal swab from both nostrils was collected every two to three days until their qualitative RT-PCR assessment became negative or study end reached. Visit 5 was conducted any time between visit 4 and Day 19, based on subject’s status change to symptom free and RT-PCR negative. If no change in such status, visit 5 conducted on Day 19 ± 2 days. If clinic visit performed on Day 19, any additional assessments made recorded on unscheduled visit eCRF page, at discretion of the investigator.

^g^ Swab from nose collected for qualitative RT-PCR at visit 5 only if RT-PCR is positive on Day 8 (visit 4). If RT-PCR is negative at visit 3 (Day 4) no swab collected for RT-PCR on Day 8 (visit 4) or at visit 5.

^h^ Only chest x-ray conducted at screening visit. At visit 3 (Day 4), based on investigator discretion, chest x-ray or CT scan performed.

***** A synthetic Dacron tipped swab was used for the RT-PCR collections, i.e., non-rayon, non-foam, recommended by the Indian Council of Medical Research (ICMR). Extracted viral genetic material was eluted into a viral transport medium, split and transported by temperature-controlled cold chain processes (2-8°C), and stored at -20°C until assayed (with 72 hours). The quantitative virus RNA was assessed using a TRUPCR Kit (3B BlackBio Biotech India Ltd). Oligonucleotide primers and Taqman probes are used for the detection of two regions of the viral N protein gene region of SARS-CoV-2: N, RdRP/Human Rnase P gene (cDNA). Assay run was 40 cycles in duration, with a clinical cycle threshold (Ct) cut off at 35 cycles.

# **Table S3. Computer-Generated Randomization Procedure**

| **Randomisation Procedure** | **Statistician/Qualifications** |
| --- | --- |
| Subjects were assigned to treatments based on a computer-generated randomization scheme generated by an independent statistical programmer. Dummy randomization scheme were reviewed and approved by the Glenmark blinded statistician. Based on dummy approved randomization scheme, live randomization scheme were generated by independent statistical programmer. | Wen Wu, PhD (Dual PhD), Fellow of the Royal Society of Chemistry (FRSC), Chartered Scientist, Member of the editorial advisory board of an international journal of Chemometrics and Intelligent Laboratory System. Currently the Chief Statistician at Glenmark Pharmaceuticals Limited, Watford, United Kingdom. |

# **Table S4. In Vitro Antiviral Activity of NONS and Placebo**

| **Nitric Oxide Nasal Spray (NONS)** | **Placebo (matching)** |
| --- | --- |
| NONS inactivates all respiratory pathogens (H1N1, RSV, HRV-14, and all VOCs (including Omicron) of SARS-COV-2) (Figure S1) by the same mechanism. NONS has an immediate multi-modal action which interferes with the structural integrity of the virus, including the disruption of viral spike (S) protein interaction with nasal host ACE-2 receptors and virus protease inhibition altering viral replication (in addition to low pH and mechanical/virus trapping barrier production).  **Figure S1. In Vitro NONS Antiviral Activity Across Multiple Respiratory Pathogens**^26^   Virus titer for control test (growth media, dark bars) and NONS (2 minutes exposure, light bars). Limit of detection (0.7 log_10_(CCID_50_)/0.1 mL, dotted line). ^*^Mean values below level of detection. | Placebo may have been slightly active. An independent antiviral assessment demonstrated this solution reduced H1N1 concentration by 1.5 log10 pfu/mL (5 log10 pfu/mL at baseline – 3.5 log10 pfu/mL at 2 minutes of contact using a comparable clinical dose volume (0.45 mL). No other ingredient or combination of ingredients without NO reduced the viral concentration. *Note these investigations were not with SARS-CoV-2.*  NONS reduced N1H1 concentrations by > 4.3 log10 pfu/mL (5 log10 pfu/mL at baseline – 0.7 log10 pfu/mL [lowest level of detection] at 2 minutes of contact using a comparable clinical dose volume (0.45 mL) in the same laboratory investigation. *Note these investigations were not with SARS-CoV-2.* |

# **Table S5. Characteristics of the Patients at Baseline (mITT high risk population, mITT unvaccinated population and mITT population)**

|  |  | **High Risk mITT*** | | **Unvaccinated mITT** | | **mITT** | |
| --- | --- | --- | --- | --- | --- | --- | --- |
| **Parameter** |  | NONS | PLC | NONS | PLC | NONS | NONS |
| Age (Year) | N | 64 | 69 | 44 | 46 | 105 | 102 |
|  | Mean | 42·0 | 43·7 | 36·3 | 39·8 | 37·3 | 39·7 |
|  | SD | 14·44 | 11·80 | 12·49 | 11·77 | 13·45 | 12·05 |
|  | Median | 40·5 | 46·2 | 34·8 | 38·4 | 34·8 | 39·2 |
|  | Range | 19-69 | 19-69 | 19-64 | 22-68 | 18-69 | 19-68 |
| Weight (Kg) | N | 64 | 69 | 44 | 46 | 105 | 102 |
|  | Mean | 63·33 | 64·33 | 61·85 | 62·17 | 63·47 | 64·14 |
|  | SD | 9·264 | 9·847 | 8·472 | 9·487 | 9·777 | 9·772 |
|  | Median | 63·85 | 63·80 | 62·70 | 62·10 | 64·30 | 64·40 |
|  | Range | 42-85 | 40-94 | 42-80 | 40-86 | 42-92 | 40-94 |
| Age subgroup (Year) |  |  |  |  |  |  |  |
| < 45 | n (%) | 36 (56·3) | 31 (44·9) | 34 (77·3) | 30 (65·2) | 77 (73·3) | 64 (62·7) |
| ≥ 45 | n (%) | 28 (43·8) | 38 (55·1) | 10 (22·7) | 16 (34·8) | 28 (26·7) | 38 (37·3) |
| Sex |  |  |  |  |  |  |  |
| Female | n (%) | 21 (32·8) | 22 (31·9) | 13 (29·5) | 15 (32·6) | 38 (36·2) | 40 (39·2) |
| Male | n (%) | 43 (67·2) | 47 (68·1) | 43 (70·5) | 31 (67·4) | 67 (63·8) | 62 (60·8) |
|  |  |  |  |  |  |  |  |
| Ct value at baseline | N | 64 | 69 | 44 | 46 | 105 | 102 |
| Median^a^ |  | 23 | 21 | 23 | 20.5 | 23 | 22 |
| < 30 | n (%) | 58 (90·6) | 67 (97·1) | 40 (90·9) | 45 (97·8) | 98 (93·3) | 97 (95·1) |
| ≥ 30 | n (%) | 6 (9·4) | 2 (2·9) | 4 (9·1) | 1 (2·2) | 7 (6·7) | 5 (4·9) |
| Co-morbidities |  |  |  |  |  |  |  |
| No | n (%) | 50 (78·1) | 57 (82·6) | 37 (84·1) | 44 (95·7) | 91 (86·7) | 90 (88·2) |
| Yes** | n (%) | 14 (21·9) | 12 (17·4) | 7 (15·9) | 2 (4·3) | 14 (13·3) | 12 (11·8) |
| Vaccination Status |  |  |  |  |  |  |  |
| Unvaccinated | n (%) | 44 (68·8) | 46 (66·7) | 44 (100) | 46 (100) | 44 (41·9) | 46 (45·1) |
| Vaccinated | n (%) | 20 (31·3) | 23 (33·3) | - | - | 61 (58·1) | 56 (54·9) |
| Dose 1 | n (%) | 20 (31·3) | 23 (33·3) | - | - | 61 (58·1) | 56 (54·9) |
| Dose 2 | n (%) | 18 (28·1) | 18 (26·1) | - | - | 54 (51·4) | 44 (43·1) |

Percentages were based on the total number of subjects in each treatment.

^a^Range of Ct values (14 to 35) were the same for both treatment groups for all populations, except for the unvaccinated mITT placebo group range which was 14 to 30.

*High-risk defined those unvaccinated or have co-morbidity or who are ≥ 45 years of age.

**Diabetes, Hypertension, Obesity, Cardiovascular risk, or Cerebrovascular risk.

**Efficacy**

# **Table S6. Summary of SARS-CoV-2 Viral RNA Values (log10 copies per mL) at Day 1 (Baseline), Day 2, Day 3, Day 4, and Day 8 (mITT High Risk Population)**

|  | | NONS  (N=64) _________________________ | | PLACEBO  (N=69) _________________________ | |
| --- | --- | --- | --- | --- | --- |
| Visit | Statistics | Observed | Change from Baseline | Observed | Change from Baseline |
|  | | | | | |
| Day 1 | n | 64 |  | 69 |  |
|  | Mean (SD) | 6⋅96 (1·506) |  | 7·16 (1·532) |  |
|  | Median | 6·79 |  | 7·30 |  |
|  | Min., Max. | 2·56, 10·10 |  | 2·58, 10·81 |  |
|  | | | | | |
| Day 2 | n | 64 | 64 | 69 | 69 |
|  | Mean (SD) | 5·86 (1·511) | -1·10 (1·603) | 6·71 (1·565) | -0·45 (2·036) |
|  | Median | 5·80 | -0·92 | 6·78 | -0·39 |
|  | Min., Max. | 2·56, 10·03 | -5·03, 1·77 | 2·54, 9·36 | -5·32, 4·72 |
|  | | | | | |
| Day 3 | n | 39 | 39 | 40 | 40 |
|  | Mean (SD) | 5·09 (1·926) | -2·02 (2·265) | 5·98 (1.696) | -1·20 (2·083) |
|  | Median | 5·50 | -1·92 | 6.25 | -1·26 |
|  | Min., Max. | 2·54, 9·60 | -6·89, 1·91 | 2·54, 8·91 | -5·35, 4·79 |
|  | | | | | |
| Day 4 | n | 61 | 61 | 63 | 63 |
|  | Mean (SD) | 3·75 (1·792) | -3·22 (2·310) | 4·16 (1·944) | -2·99 (2·578) |
|  | Median | 2·54 | -3·62 | 2·74 | -3·66 |
|  | Min., Max. | 2·54, 9·04 | -7·56, 2·34 | 2·54, 8·64 | -8·27, 4·41 |
|  | | | | | |
| Day 8 | n | 50 | 50 | 51 | 51 |
|  | Mean (SD) | 3·09 (1·315) | -3·80 (1·890) | 3·13 (1·233) | -4·08 (1·826) |
|  | Median | 2·54 | -3·84 | 2·54 | -4·40 |
|  | Min., Max. | 2·54, 7·86 | -7·56, 3·19 | 2·54, 7·44 | -8·27, 0·53 |
|  | | | | | |

# **Table S7. Mean SARS-CoV-2 Viral Load (log10 copies per mL) Change from Baseline Through Day 8 in Adult COVID-19 Infected Patients (mITT Unvaccinated and mITT Populations: MMRM)**

| Population | Statistics | NONS | PLACEBO |
| --- | --- | --- | --- |
| **mITT Unvaccinated** |  | (N=44) | (N=46) |
|  | LSM (SE) | -2·67 (0·178) | -2·08 (0·179) |
|  | 95% CI | -3·02, -2·32 | -2·43, -1·72 |
|  | Difference: LSM (SE) | -0·60 (0·253) |  |
|  | 95% CI of difference | -1·09, -0·10 |  |
|  | p-value^1^ of difference | 0·019 |  |
|  |  |  |  |
| **mITT** |  | (N=105) | (N=102) |
|  | LSM (SE) | -2·51 (0·114) | -2·17 (0·118) |
|  | 95% CI | -2·73, -2·28 | -2·40, -1·94 |
|  | Difference: LSM (SE) | -0·34 (0·161) |  |
|  | 95% CI of difference | -0·65, -0·02 |  |
|  | p-value of difference | 0·036 |  |

CFB=Change from baseline.

Mean change from baseline in viral load log values through Day eight (7 days of therapy) was analysed by MMRM. The difference LSM(SE) between groups was calculated for NONS vs placebo (NONS-placebo). Patients having positive RT-PCR at screening and up to 1-day post randomization were considered. The 95% confidence interval (CI) for the LSM mean difference between groups was calculated for NONS minus placebo. p-values were calculated for the comparison of treatment groups with treatment as main effect and by considering visit, baseline value, risk factor (high risk yes/no), and treatment by visit interaction as covariates.

# **Table S8. Mean SARS-CoV-2 Viral Load (log10 copies per mL) Change from Baseline Through Day 8 in Adult COVID-19 Infected Patients (High Risk Per Protocol Population [PP]: MMRM)**

|  | Statistics | NONS (N=63) | PLACEBO (N=66) |
| --- | --- | --- | --- |
|  | LSM (SE) | -2·62 (0·145) | -2·12 (0·143) |
|  | 95% CI | -2·90, -2·33 | -2·40, -1·84 |
|  | Difference: LSM (SE) | -0·49 (0·204) |  |
|  | 95% CI of difference | -0·90, -0·09 |  |
|  | p-value of difference | 0·016 |  |

CFB=Change from baseline.

Mean change from baseline in viral load log values through Day eight (7 days of therapy) was analysed by MMRM. The difference LSM(SE) between groups was calculated for NONS vs placebo (NONS-placebo). Patients having positive RT-PCR at screening and up to 1-day post randomization were considered. The 95% confidence interval (CI) for the LSM mean difference between groups was calculated for NONS minus placebo. p-values were calculated for the comparison of treatment groups with treatment as main effect and by considering visit, baseline value, risk factor (high risk yes/no), and treatment by visit interaction as covariates.

# **Table S9. Mean SARS-CoV-2 Viral Load (log10 copies per mL) Change from Baseline Through Day 8 in Adult COVID-19 Infected Patients (mITT High Risk and mITT Populations: MMRM with vaccinated status added as an additional covariate)**

| Population | Statistics | NONS | PLACEBO |
| --- | --- | --- | --- |
| **mITT High Risk** |  | (N=63) | (N=66) |
|  | LSM (SE) | -2.61 (0.149) | -2.09 (0.146) |
|  | 95% CI | [-2.91 , -2.32] | [-2.38 , -1.81] |
|  | Difference: LSM (SE) | -0.52 (0.203) |  |
|  | 95% CI of difference | [-0.92 , -0.12] |  |
|  | p-value of difference | 0.011 |  |
|  |  |  |  |
| **mITT** |  | (N=105) | (N=102) |
|  | LSM (SE) | -2.52 (0.119) | -2.19 (0.123) |
|  | 95% CI | [-2.76 , -2.29] | [-2.43 , -1.94] |
|  | Difference: LSM (SE) | -0.34 (0.161) |  |
|  | 95% CI of difference | [-0.65 , -0.02] |  |
|  | p-value of difference | 0.037 |  |
|  |  |  |  |

CFB=Change from baseline.

Mean change from baseline in viral load log values through Day eight (7 days of therapy) was analysed by MMRM. The difference LSM(SE) between groups was calculated for NONS vs placebo (NONS-placebo). Patients having positive RT-PCR at screening and up to 1-day post randomization were considered. The 95% confidence interval (CI) for the LSM mean difference between groups was calculated for NONS minus placebo. p-values were calculated for the comparison of treatment groups with treatment as main effect and by considering visit, baseline value, risk factor (high risk yes/no), vaccinated status (Yes/No), and treatment by visit interaction as covariates.

The coefficients of the vaccinated status (Coef=0.2148, SE=0.2816, t-value=0.7630, p-value=0.446) and interaction with the treatment (Coef=-0.1859, SE=0.3905 t-value=-0.4760, p-value=0.634) were not significant, indicating that vaccinated status had no impact on the mITT high risk population treatment effect. The same conclusion was obtained for the mITT population.

# **Table S10. Analysis of Time to Negative Conversion of SARS-CoV-2 RT-PCR (qualitative; mITT Unvaccinated and mITT Populations)**

| Population | Statistics | NONS | PLACEBO |
| --- | --- | --- | --- |
| **mITT Unvaccinated** |  | (N=44) | (N=46) |
|  | Number of events (%) | 43 (97·7) | 46 (100) |
|  | Time to event in days |  |  |
|  | Median (95% CI) | 4·0 (4·0, 8·0) |  |
|  | p-value | 0·0237 |  |
|  | Hazard Ratio (95% CI) | 1·396 (0·890, 2·189) |  |
|  |  |  |  |
| **mITT** |  | (N=105) | (N=102) |
|  | Number of events (%) | 102 (97·1) | 100 (98·0) |
|  | Time to event in days |  |  |
|  | Median (95% CI) | 4·0 (4·0, 8·0) |  |
|  | p-value | 0·3511 |  |
|  | Hazard Ratio (95% CI) | 1·069 (0·802, 1·424) |  |

Patients having positive RT-PCR at screening and up to 1-day post randomization were considered. Kaplan-Meier used to estimate the median duration of those time-to-events and 95% confidence intervals. Two treatment arms were compared using log-rank test to estimate the p-value. The hazard ratio of NONS/ Placebo was computed based on the Cox regression model. Subjects who terminated the study without documented event were censored at day 19.

# **Table S11. Proportion of COVID-19 Infected Patients with ≥ 2 Point Clinical Status Improvement with WHO Progression Scale Score (mITT Unvaccinated and mITT Population)**

| Population | NONS | PLACEBO | Treat Difference (p-value) |
| --- | --- | --- | --- |
| **mITT Unvaccinated** | (N=44) | (N=46) |  |
| Day 8 | 84·1% | 65·2% | 18·9 (0·054) |
| Day 16 | 95·5% | 89·1% | 6·3 (0·435) |
| Day 18 | 97·7% | 93·5% | 4·2 (0·617) |
| Day 20 | 100% | 100% |  |
|  |  |  |  |
| **mITT** | (N=105) | (N=102) |  |
| Day 8 | 71·4% | 68·6% | 2·8 (0·762) |
| Day 16 | 89·5% | 87·3% | 2·3 (0·668) |
| Day 18 | 92·4% | 90·2% | 2·2 (0·628) |
| Day 20 | 100% | 100% |  |

Patients having positive RT-PCR at screening and up to 1-day post randomization were considered. Percentages were based on the total number of subjects in respective treatment group. Difference % was calculated for NONS - Placebo. p-value was calculated for between groups comparison by using fisher exact test.

**Safety**

# **Table S12. NONS Safety - Lack of Vasodilation Potential from Locally or Systemically Absorbed Nitric Oxide and Expected Impact on Nasal Immunity (Safety Analysis Population)**

| I. Systemic vasodilation is a known adverse event associated with the therapeutic administration of high-dose continuous inhalation of NO gas, associated with the absorption of NO through the pulmonary alveoli-capillary system. | | | | |
| --- | --- | --- | --- | --- |
| Systemic: No adverse events of hypotension, syncopal episode and/or dizziness were observed in either treatment group. Blood pressures was monitored at baseline and over the course of the seven days of treatment. The use of NONS did not change systolic or diastolic blood pressures from baseline compared to placebo over the 7 days of treatment (Table S12.1). | | | | |
| **Table S12.1 Blood Pressure (vital)** | **NONS (N=153)** | | **PLACEBO (N=153)** | |
| Change from baseline to Day 8 | Baseline  Mean (SD) | EOT (Day 8)  Mean  [CFB (SD)] | Baseline  Mean (SD) | EOT (Day 8)  Mean  [CFB (SD)] |
| Systolic Blood Pressure (mmHg) | 123.3 (7.5) | 122.2  [-1.3 (6.3)] | 122.6 (8.6) | 121.6  [-0.7 (6.3)] |
|  |  |  |  |  |
| Diastolic Blood Pressure (mmHg) | 79.6 (6.3) | 79.0  [-0.1 (5.8)] | 80.2 (5.4) | 79.0  [-0.9 (5.7)] |
| EOT=End of treatment. SD=standard deviation. CFB=Change from baseline. Values are mean; no CFB outliers were observed.  Nasal: Neural mechanisms contribute to many nasal symptoms and syndromes. Sensory nerve stimulation by irritants, mast cell products, and inflammatory mediators leads to sneezing and other systemic reflexes. Parasympathetic reflexes and sensory axon responses combine to increase nasal blood flow, fill venous sinusoids (which thickens the mucosa and reduces nasal patency), induce plasma extravasation, and stimulate glandular secretion of mucous and serous cell products.^1^  The use of NONS did not induce additional or worsening of nasal vasodilation symptoms (sneezing, congestion, and/or rhinorrhea) compared to placebo treatments; none of these symptoms were reported as adverse events in either group. These observations of the maintenance of normotension and improvement of nasal symptoms support the position the NO gas generated from NONS lacks local nasal or systemic absorption.  II. Nasal Immunity: The nasal mucosa routinely filters, moistens, and warms the inhaled air to minimize its irritative effects on lower airways while maintaining its innate immunity mechanism (mucociliary clearance barrier system). The nasopharynx-associated lymphoid tissue (NALT), located in the most cranial pharyngeal mucosa constitutes the other important acquired immune (antigen-specific) mechanism.^2^ The low NO concentrations generated exogenously and released from NONS is the same ubiquitous gas found throughout the body, remains local to the nasopharynx, lacks systemic absorption and would not be expected to negatively impact the immune systems. The HPMC component within NONS was designed to positively impact and augment the innate nasal barrier system (trapping the virions). The other NONS ingredients, are generally regarded as safe (GRAS) and would not be expected to negatively impact the generation of Th1- and Th2-polarized lymphocytes, IgA-committed B cells, and the other cellular elements (i.e., dendritic cells, microfold cells, macrophages, nasal epithelial ciliated and goblet cells) of the acquired immunity system. | | | | |

**REFERENCES**

S1. Baraniuk J. Sensory, parasympathetic, and sympathetic neural influences in the nasal mucosa. J Allergy Clin Immunol 1992;90:1045-50.

S2. Gallo O, Locatello L, Mazzoni A, Novelli L, Francesco Annunziato F. The central role of the nasal microenvironment in the transmission, modulation, and clinical progression of SARS- CoV-2 infection. Mucosal Immunology (2021) 14:305–316; https://doi.org/10.1038/s41385-020-00359-2.
