# Supplementary material for: SARS-CoV-2 accelerated clearance using a novel nitric oxide nasal spray (NONS) treatment: A randomized trial
Source: Lancet Reg Health Southeast Asia. 2022 Jun 29;3:100036. doi: 10.1016/j.lansea.2022.100036 (PMC9239922; doi:10.1016/j.lansea.2022.100036)
Supplement: Supplementary file 3 [file mmc3.docx]

Statistical Analysis plan

pHASE iIi

Version: 3.0

date of plan: 25-JAN-2022

Based on:
Original protocol, Version 6.0, 31-dec-2021

CRF, Version 4.0, 08-oct-2021

STUDY DRUG: Nitric Oxide Nasal Spray

Protocol Number: GPL/CT/2021/004/III

STUDY TITLE: A RANDOMIZED, DOUBLE-BLIND, PARALLEL ARM, MULTICENTER STUDY TO EVALUATE THE EFFICACY AND SAFETY OF NITRIC OXIDE NASAL SPRAY COMBINED WITH STANDARD SUPPORTIVE CARE IN ADULT NON-HOSPITALIZED PATIENTS WITH COVID-19

Sponsor:

*Glenmark Pharmaceuticals Ltd*

*Glenmark House,*

*B.D.Sawant marg,*

*Chakala,Andheri East,*

*Mumbai-400 099*

*India*

Document Version History

| **Version Number** | **Version Date** | **Description of change** |
| --- | --- | --- |
| 1.0 | 02-DEC-2021 | 1st version based on protocol version 5.0 |
| 2.0 | 01-JAN-2022 | 2nd version based on protocol version 6.0 |
| 3.0 | 25-JAN-2022 | On page 11, the following sentences:   - Resolution of all the COVID-19 symptoms present at baseline. End date of last symptom (except cough) will be the date of clinical recovery. For cough, date of reaching symptom level 1 or 0 will be considered as date of resolution. - Resolution of common flu-like symptoms fever, cough and headache. End date of last of the three symptoms (except cough) will be the date of clinical recovery. For cough, date of reaching symptom level 1 or 0 will be considered as date of resolution. Subjects with at least one of these 3 symptoms at baseline will be included in this analysis.   were updated into:   - Resolution of all the COVID-19 symptoms present at baseline. End date of last symptom will be the date of clinical recovery. - Resolution of common flu-like symptoms fever, cough and headache. End date of last of the three symptoms will be the date of clinical recovery. Subjects with at least one of these 3 symptoms at baseline will be included in this analysis.   On pages 39-40, the following sentence was added:  As RT-PCR conversion as early as day 2 suggests late COVID-19 infection at the time of screening and not appropriate to study treatment difference, separate analysis will be conducted including and excluding subjects who become RT-PCR negative on Day 2.  On page 44, the following sentence were added:  Similarly, proportion of immediate contacts of the patients having symptoms of COVID-19 and proportion of immediate contacts of the patients with positive COVID-19 test results will be analyzed using the chi-square test or Fisher’s exact test. |

This study is being conducted in compliance with good clinical practice, including the archiving of essential documents.

Technical Summary Report (TSR)

| **Name of Sponsor/Company**  **Glenmark Pharmaceuticals Ltd** | **Individual Study Table Referring to Part of the Dossier:**  **Volume:** | ***(For National Authority Use Only)*:** |
| --- | --- | --- |
| **Name of Finished Product:**  Nitric oxide nasal spray | **Page:** |  |
| **Name of Active Ingredient:**  Nitric oxide nasal spray Favipiravir Favipiravir Favipiravir Favipiravir Favipiravir Favipiravir Favipiravir Favipiravir Favipiravir Favipiravir Favipiravir Favipiravir Favipiravir Favipiravir Favipiravir Favipiravir Favipiravir Favipiravir Favipiravir |  |  |
| **Title Of Study:**  **A Randomized, Open-Label, Multicenter Study To Evaluate The Efficacy And Safety Of Favipiravir Combined With Standard Supportive Care In Adult Indian Patients With Mild To Moderate COVID-19.**A Randomized, Open-Label, Multicenter Study To Evaluate The Efficacy And Safety Of Favipiravir Combined With Standard Supportive Care In Adult Indian Patients With Mild To Moderate COVID-19.A Randomized, Double-blind, Parallel Arm, Multicenter Study To Evaluate The Efficacy And Safety Of Nitric Oxide Nasal Spray Combined With Standard Supportive Care In Adult non-hospitalized Patients With COVID-19. | | |
| **Investigators**:  Study Center(s): 14 centers | | |
| **Studied period:**  Maximum of 18 days from randomization | **Phase of development:** Phase III | |
| **Objectives:**  Primary:  • The primary objective of this study is to evaluate the efficacy of Nitric Oxide Nasal Spray combined with standard supportive care compared with standard supportive care alone in adult subjects with COVID-19 not requiring hospitalization.  Secondary:  • The secondary objective is to evaluate the safety and tolerability of Nitric Oxide Nasal Spray combined with standard supportive care compared with standard supportive care alone in adult subjects with COVID-19 not requiring hospitalization. | | |
| **Methodology:**  This is a randomized, double-blind, multi-center, parallel arm, clinical study evaluating the efficacy and safety of Nitric Oxide Nasal Spray with standard supportive care vs standard supportive care alone in patients with COVID-19 not requiring hospitalization.  306 eligible patients will be randomized in a 1:1 ratio into 2 groups: one group will receive Nitric Oxide Nasal Spray (NONS) along with standard supportive care and the control group will receive standard supportive care along with placebo of NONS. Standard supportive care will be provided in accordance with latest guidelines issued by Ministry of Health and family welfare; Government of India. Treatment duration is 7 days (up to Day 8 visit) and the total study duration will be maximum for 18 days from randomization (up to Day 19 visit). Viral load will be estimated on Day 1 (baseline), Day 2 (post-24 hours of dosing), Day 4 (post 72 hours of dosing) and Day 8. Patients may be detained in a facility to facilitate protocol assessments for first 7 days or may be treated at home based on investigator discretion. Subjects who are treated at home will visit the study center on Day 1, 4, and 8 for study assessments and Day 2 visit (and swab sample collection for visit 2) will be conducted at home. If subjects are not able to visit the study center on any of the days, telephonic or video-conference and home based assessments can be conducted. | | |
| **Number of Subjects (planned and analyzed):** 306 subjects will be randomized in the study.  **Determination of Sample size:**  Based on the data of phase 2 study of Nitric Oxide Nasal Spray in the United Kingdom (Clinical Study Report IRAS ID 287727 NONS COVID Study), assuming a treatment effect of 5.0 log viral load in the primary endpoint, with a standard deviation of 10.0, 172evaluable subjects (86 subjects per arm) will provide a power of 90% with two-sided significance level of 5 %. For the secondary endpoint (RT-PCR conversion), assuming RT-PCR conversion of 40% in the Placebo group by Day 8, 260 subjects (130 subjects in each arm) will provide a power of 90% at two-sided significance level of 5 %, to detect a treatment difference of 20% in proportion of subjects achieving RT-PCR conversion.  Assuming a dropout rate of 15 %, total 306 subjects (approx. 153 subjects per arm) will be randomized. | | |
| **Main criteria for inclusion:**  Each subject must meet all of the following criteria to be entered into the randomized treatment in the study:  1. Voluntarily participating in the clinical study; fully understanding and being fully informed of the study and having signed the Informed Consent Form (ICF); willingness and capability to complete all the study procedures  2. Age 18-70 years (inclusive) at the time of signing ICF  3. Patients with laboratory confirmation of infection with SARS-CoV-2 by positive Rapid Antigen Test for SARS-CoV-2 at screening.  4. Recent onset (within 72 hours of time of consent) symptoms of mild COVID-19 with oxygen saturation (SpO_2_ > 94 %) and respiratory rate < 24 breaths/min. Any of the COVID-19 like symptoms including fever, cough, sore throat, malaise, headache, nasal congestion, muscle pain, gastrointestinal symptoms, lack of taste or smell without shortness of breath or dyspnea; (the maximum permitted difference in the time of onset of symptoms and the time of consent is 48 hours)  5. For female subjects: evidence of post-menopause, or, for pre-menopause subjects, negative pretreatment urine pregnancy test  6. Eligible subjects of child-bearing age (female or male with female partner of childbearing age) must agree to take effective contraceptive measures (including hormonal contraception, barrier methods or abstinence) with his/her partner during the study period and for at least 7 days following the last study treatment.  7. Not participating in any other interventional drug clinical studies before completion of the present study.  **Main criteria for exclusion:**  A subject who meets any of the following criteria must not be entered into the randomized treatment in the study:  Where, in the opinion of the investigator, participation in this study will not be in the best interest of the subject, or any other circumstances that prevent the subject from participating in the study safely  Subjects with infection requiring oxygen support, invasive or non-invasive ventilator support, extracorporeal membrane oxygenation (ECMO) or shock requiring vasopressor support.  Current known pneumonia based on x‑ray or computed tomography (CT) scan or history of pneumonia within 3 months before screening.  Requiring hospitalization for the treatment of COVID‑19  Prolonged QT, defined as QTcF ≥ 450 milliseconds for men and as QTcF ≥ 470 milliseconds for women  History of known severely reduced left ventricular (LV) function (Ejection fraction < 30 %)  Requires ICU care for management of ongoing clinical status.  Known allergy or hypersensitivity to Nitric Oxide Nasal Spray.  History of known severe renal impairment [creatinine clearance (CrCl) < 30 mL/min] or having received continuous renal replacement therapy, hemodialysis or peritoneal dialysis;  Asthma, allergic rhinitis or chronic obstructive lung disease  Psychiatric disease that is not well controlled (controlled defined as stable on a regimen for more than one year).  Pregnant or lactating women;  Having used Nitric Oxide Nasal Spray or participated in any other interventional drug clinical study within 30 days prior to first dose of study drug.  Subjects who have received hydroxychloroquine within 7 days before screening or subjects who require hydrochloroquine treatment.  Subjects who have received intranasal medication/treatment within 7 days before screening or subjects who require use of any intranasal medication.  Subjects who have received medications with antiviral effect such as remdesivir, favipiravir, oseltamivir, ivermectin or inhaled corticosteroids within 7 days before screening or subjects who require use of any of these medications.  Subjects using nitric oxide donor agents such as prilocaine, sodium nitroprusside and nitroglycerine | | |
| **Test product, dosage and mode of administration:**  Nitric oxide nasal spray, administered as a nasal spray, at a dose of two sprays each nostril, six times a day (along with standard supportive care). Study medication should be administered upon awakening (Dose 1), then administer additional doses (Dose 2 to 6) approximately every 2-3 hours while awake. Doses should be separated by at least 1.5 hours. Preferably, administer the last dose of the day (Dose 6) at bedtime.  Investigational Drug Products will be Manufacture and Supplied by SaNOtize Research and Development Corp. 25th Floor, 700 West Georgia Street, Vancouver, BC, Canada [Manufactured at Nextar Chempahrm Solutions Ltd. 13B Einstein St. Weizmann Science Park, Ness Ziona 74140 Israel.] | | |
| **Duration of treatment:**  Maximum 7 days | | |
| **Placebo therapy :**  Placebo of Nitric oxide nasal spray, administered as a nasal spray, at a dose of two sprays each nostril, six times a day along with Standard supportive care. | | |
| **Criteria for evaluation (see protocol):** | | |
| **Study endpoints:**  **Primary Endpoint:**   - Change from baseline in log viral load through Day 8   **Secondary Endpoints:**   - Proportion of subjects with negative conversion of SARS-CoV 2 RT PCR on Day 2, 3, 4, or 8. - Determine effect of NO nasal spray on clinical recovery [Time Frame: 18 days]. Determine the time to clinical recovery in participants with COVID-19 by measuring the proportion of patients from enrollment to resolution of baseline flu-like symptoms. - Proportion of subjects requiring hospitalization for the treatment of COVID‑19 [Tim frame: 18 days] - Proportion of patients achieving a 2 point worsening in WHO Progression scale on Day 2, 3, 4, 8 and 19 - Time from randomization to first time use of high flow supplemental oxygen/non-invasive ventilation/mechanical ventilation/ extracorporeal membrane oxygenation. - Change from baseline in COVID-19 related symptom score of participants with COVID- 19 at Day 2, 3, 4, 8 and 19 - Change from baseline in log Viral load at Day 2, 3, 4, and 8 [Time Frame: 7 days] - Safety and tolerability of 7 day administration of NO nasal spray treatment over 18 days [Time Frame: 18 days]. Measure the tolerability of the NO nasal spray treatment as determined by number of adverse events, pain, discomfort or discontinuations of treatment. | | |
| **Statistical methods:**  **Analysis Sets**  Analysis of the primary endpoint will be conducted using the modified ITT (mITT) analysis set. In addition, a supportive analysis will be performed for the primary efficacy endpoint using the Per Protocol Set (PPS).  **mITT analysis set**  The mITT analysis set will include all randomised subjects who received at least one dose of study medication, who have a non-missing baseline measurement and at least one post-baseline efficacy measurement for primary efficacy variable. Subjects with negative RT-PCR at baseline will not be included in the mITT analysis set.  **Per protocol analysis set**  The per protocol analysis set (PPS) will include all subjects who are randomized, received at least one dose of study medication, completed the study and do not have any major protocol deviations. Major protocol deviations will be discussed and decided at the blinded data review meeting (BDRM) meeting before database lock.  **Safety analysis set**  The Safety analysis set (SAS) will include all subjects who are randomized and received at least one dose of study medication. All safety endpoints will use the safety analysis set unless otherwise specified.  **Analysis of Primary Efficacy Endpoints**  Subjects with negative RT-PCR at baseline will be excluded from the analysis.  Change from baseline in log_10_ viral load through Day 8 will be measured using the Average of viral load (log_10_ copies per milliliter) between day 1 to each visit day up to day 8.  As a sensitivity analysis, change from baseline in log_10_ viral load through Day 8 will also be measured using the AUC of viral load (log_10_ copies per milliliter) between day 1 to each visit day up to day 8 normalized by day. The time-weighted average change from baseline (day 1) to each visit will be calculated for each subject as the area under the concentration–time curve, with the use of the linear trapezoidal rule for change from baseline divided by the time interval of the observation period.  The comparison of Nitric Oxide versus Placebo in the Average of viral load or AUC will be analyzed using MMRM method. The MMRM model will include data from all visits until day 8 and the following covariates: treatment, visit, baseline value (log10 copies per milliliter), risk factor (high risk yes/no), center, and treatment by visit interaction; unstructured covariance matrix will be used, thus allowing adjustment for correlations between the time points within subjects.  Primary analysis will be conducted in the mITT population and sensitivity analysis will be conducted in the PP population. ANCOVA with LOCF analysis will be done in mITT as the sensitivity analysis as well.  Sub-group analysis for high risk population and based on vaccination status sub-groups (yes/no), co-morbidity sub-group (yes/no) and baseline viral load sub-groups (>3, >4 and >5) will also be conducted.  **Analysis of Secondary Efficacy Endpoints**  Proportion of subjects with negative conversion of SARS-CoV 2 RT PCR on Day 2, 3, 4 or 8 will be based on the qualitative RT-PCR positive/negative results and will be analyzed using the chi-square test or Fisher’s exact test. Subjects with negative RT-PCR at baseline will not be included in the analysis. Primary analysis will be conducted in the mITT population and sensitivity analysis will be conducted in the PP population.  As additional supportive analysis, the time to event (i.e. negative SARS-CoV2 RT-PCR result) will be analyzed using the Kaplan-Meier method and log-rank test and Cox model.  Sub-group analysis will be done for high and low baseline Ct value (higher and lower than median Ct value), high risk yes/no subgroups, in subgroups including and excluding subjects with worsening of COVID-19, and different age-group populations (as post-hoc analysis) for the primary endpoints.  Additional analysis will be conducted:   - using definition of RT-PCR conversion based on viral load threshold of 3 for the log_10_ copies per milliliter. - Using definition of RT-PCR conversion as change in Ct value from below 30 to 30 and above, and change in Ct value from below 35 to 35 and above.   The following time event endpoints will be analyzed the same way as the primary endpoint using the Kaplan-Meier method, log-rank test and Cox analysis. In addition, proportion of patients with events at days 2, 4, 8 and 19 will be compared between groups using the chi-square test or Fisher’s exact test:   - Time the time to clinical recovery in participants with COVID-19 by measuring the proportion of patients from enrollment to resolution of baseline flu-like symptoms. Following definitions of clinical recovery will be used: - Resolution of all the COVID-19 symptoms present at baseline. End date of last symptom will be the date of clinical recovery. - Resolution of common flu-like symptoms fever, cough and headache. End date of last of the three symptoms will be the date of clinical recovery. Subjects with at least one of these 3 symptoms at baseline will be included in this analysis.   Additionally, time to complete cure, i.e. virological cure in terms of negative conversion of qualitative RT-PCR AND clinical cure based on the above 2 definitions, will be analyzed using the Kaplan-Meier method, log-rank test and Cox analysis; and proportion of patients with events at days 2, 4, 8 and 19 will be compared between groups using the chi-square test or Fisher’s exact test:   - Time from randomization to first time use of high flow supplemental oxygen/non-invasive ventilation/mechanical ventilation/ extracorporeal membrane oxygenation.   The following endpoints will be analyzed using a Mixed Model Repeated Measure (MMRM) method. The MMRM model will include data from all visits until day 19 and the following covariates: treatment, visit, baseline, risk factor (high risk yes/no), center, and treatment by visit interaction; unstructured covariance matrix will be used, thus allowing adjustment for correlations between the time points within subjects.   - Change from baseline in COVID-19 related symptom score of participants with COVID- 19 at Day 2, 3, 4, 8 and 19. - Total symptom score will be calculated for each subject at each time point as per the US-FDA guidance and compared within and between groups. In addition, change from baseline in individual symptom score will be compared descriptively. - Change from baseline in log Viral load at Day 2, 3, 4, and 8 [Time Frame: 7 days].   In addition, percentage reduction in log viral load at each time point will be reported.  The following binary endpoints will be analyzed using the chi-square test or Fisher’s exact test.   - Proportion of subjects requiring hospitalization for the treatment of COVID 19 [Tim frame: 18 days] - Proportion of patients achieving a 2-point worsening in WHO Progression scale on Day 2, 3, 4, 8 and 19.   Sub-group analysis will be done for high risk yes/no for the secondary efficacy endpoints.  **Pharmacokinetic Analyses**  No PK parameters will be calculated.  **Interim analysis**  Interim analysis will be performed when ~50 % of the subjects complete the study according to group sequential design and sample size may be re-assessed.  Based on the pre specified analysis and as per Subject Expert Committee (SEC) recommendation dated 29/12/2021, to perform another analysis of current recruited subjects to include high risk population. | | |

Table of Contents

[1. List of Abbreviations 17](#_Toc89332292)

[2. Introduction 19](#_Toc89332293)

[3. Study Objectives and Endpoints 20](#_Toc89332294)

[3.1. Study Objectives 20](#_Toc89332295)

[3.1.1. Primary Objective 20](#_Toc89332296)

[3.1.2. Secondary Objective 20](#_Toc89332297)

[3.2. Study Endpoints 20](#_Toc89332298)

[3.2.1. Primary Endpoints 20](#_Toc89332299)

[3.2.2. Secondary Endpoints 20](#_Toc89332300)

[4. study design 22](#_Toc89332301)

[4.1. Summary of Study Design 22](#_Toc89332302)

[4.2. Definition of Study Drugs 22](#_Toc89332303)

[4.3. Sample Size Considerations 23](#_Toc89332304)

[4.3.1. Sample Size Justifications 23](#_Toc89332305)

[4.3.2. Sample Size Re-estimation 23](#_Toc89332306)

[4.4. Randomization 23](#_Toc89332307)

[4.5. Clinical Assessments 23](#_Toc89332308)

[5. Planned Analyses 27](#_Toc89332309)

[5.1. Interim Analysis 27](#_Toc89332310)

[5.2. Final Analyses 27](#_Toc89332311)

[6. General Considerations for Data Analyses and Handling 28](#_Toc89332312)

[6.1. General Summary Table and Individual Subject Data Listing Considerations 28](#_Toc89332313)

[6.2. General Post Text Summary Table and Individual Subject Data Listing Format Considerations 29](#_Toc89332314)

[6.3. Data Management 30](#_Toc89332315)

[6.4. Data Presentation Conventions 30](#_Toc89332316)

[6.5. Analysis Populations 31](#_Toc89332317)

[6.6. Definitions 32](#_Toc89332318)

[6.7. Derived and Transformed Data 32](#_Toc89332319)

[6.7.1. Baseline Age 32](#_Toc89332320)

[6.7.2. Treatment Day 32](#_Toc89332321)

[6.7.3. Change from Baseline 32](#_Toc89332322)

[6.7.4. Handling laboratory data out of the range of quantification 32](#_Toc89332323)

[6.8. Handling of Missing Data 33](#_Toc89332324)

[6.8.1. Missing Efficacy Endpoints 33](#_Toc89332325)

[6.8.2. Missing Start and Stop Dates for Prior and Concomitant Medication 33](#_Toc89332326)

[6.8.3. Missing Start and Stop Dates for Adverse Events 34](#_Toc89332327)

[7. Study Population 36](#_Toc89332328)

[7.1. Subjects Disposition 36](#_Toc89332329)

[7.2. Screen Failures 36](#_Toc89332330)

[7.3. Protocol Deviations 36](#_Toc89332331)

[7.4. Demographic and Baseline Characteristics 36](#_Toc89332332)

[7.5. Listing of Subject Inclusion and Exclusion Criteria 37](#_Toc89332333)

[7.6. Prior and Concomitant Medications 37](#_Toc89332334)

[7.7. Baseline Laboratory Data 37](#_Toc89332335)

[7.8. Baseline Primary and Secondary Efficacy Evaluations 37](#_Toc89332336)

[8. Method of Analysis 38](#_Toc89332337)

[9. Efficacy 39](#_Toc89332338)

[9.1. Analysis of the Primary Efficacy Endpoint and related secondary endpoints 39](#_Toc89332339)

[9.1.1. Primary Efficacy Endpoints Analysis 39](#_Toc89332340)

[9.1.2. Analyses of the related secondary endpoints 40](#_Toc89332341)

[9.1.3. Other additional Supportive Analyses of the Primary Efficacy Endpoints 40](#_Toc89332342)

[9.2. Analysis of other Secondary Endpoints 41](#_Toc89332343)

[9.3. Summary of Endpoint Analyses 44](#_Toc89332344)

[10. Safety Analyses 46](#_Toc89332345)

[10.1. Compliance 46](#_Toc89332346)

[10.2. Adverse Events 46](#_Toc89332347)

[10.3. Routine Laboratory Data 47](#_Toc89332348)

[10.4. Vital Signs 48](#_Toc89332349)

[10.5. Physical Examination 49](#_Toc89332350)

[10.6. Unscheduled Assessment 49](#_Toc89332351)

[10.7. Pharmacokinetic Measurements Analyses 49](#_Toc89332352)

[11. Appendix 50](#_Toc89332353)

[Appendix : Table of Contents for Data Display Specifications in the separate mock shells file. 50](#_Toc89332354)

List of Tables

[Table 1: List of Abbreviations 17](#_Toc497476875)

1. List of Abbreviations

Table 1: List of Abbreviations

| AE | Adverse event |
| --- | --- |
| ANCOVA | Analysis of covariance |
| BP | Blood pressure |
| BUN | Blood Urea Nitrogen |
| C | Control |
| COVID-19 | Coronavirus disease of 2019 |
| CRF | Case report form |
| CrCl | creatinine clearance |
| CSR | Clinical study report |
| ECG | Electrocardiogram |
| ECMO | Extracorporeal membrane oxygenation |
| HR | Hazard ratio |
| ICH | International Conference on Harmonisation |
| IP | Investigational product |
| ITT | Intention-to-treat |
| K-M | Kaplan-Meier |
| LDH | lactic acid dehydrogenase |
| MedDRA | Medical Dictionary for Regulatory Activities |
| mITT | Modified intention-to-treat |
| MMRM | Mixed model repeated measures |
| MV | mechanical ventilation |
| NIV | Non-invasive ventilation |
| PaO2 | arterial oxygen partial pressure |
| PPS | Per protocol set |
| PT | Preferred term |
| QT | Electrocardiographic QT interval from onset of Q wave to end of T wave |
| QTc | QT interval corrected for HR |
| R | a free software environment for statistical computing and graphics |
| REML | Restricted Maximum-Likelihood |
| RT-PCR | Reverse transcription polymerase chain reaction |
| SAE | Serious adverse event |
| SAS | SAS (previously "Statistical Analysis System") is a statistical software suite developed by SAS Institute for data management, advanced analytics, multivariate analysis, business intelligence, criminal investigation, and predictive analytics |
| SAP | Statistical analysis plan |
| SARS-CoV2 | Severe acute respiratory syndrome coronavirus 2 |
| SOC | System organ class |
| SD | Standard deviation |
| SpO2 | peripheral capillary oxygen saturation |
| T | Test drug |
| TEAE | Treatment emergent adverse event |
| WHO | World Health Organization |

1. Introduction

The purpose of this statistical analysis plan (SAP) is to describe the planned analyses and data displays to be included in the Clinical Study Report (CSR) for Protocol GPL/CT/2021/004/III

| Protocol Revision Chronology: | | |
| --- | --- | --- |
| Protocol | 01-Jul-2021 | Original |
| Amendment 1 | 19-Jul-2021 | Version 2.0 |
| Amendment 2 | 05-Aug-2021 | Version 3.0 |
| Amendment 3 | 07-Sep-2021 | Version 4.0 |
| Amendment 4 | 04-Oct-2021 | Version 5.0 |
| Amendment 5 | 31-Dec-2021 | Version 6.0 |

This SAP was developed in accordance with ICH E9 guideline. All decisions regarding final analysis, as defined in this SAP document, will be made prior to Database Freeze of the study data. Further information can be found in the protocol.

1. Study Objectives and Endpoints

This document describes the Statistical Analysis Plan (SAP) for Glenmark Pharmaceuticals Limited, Protocol GPL/CT/2021/004/III with study entitled “A Randomized, Double-blind, Parallel Arm, Multicenter Study To Evaluate The Efficacy And Safety Of Nitric Oxide Nasal Spray Combined With Standard Supportive Care In Adult non-hospitalized Patients With COVID-19.”.

This SAP will be developed and finalized prior to database lock.

Any changes to the SAP will require the new version of SAP to be released. The responsible parties at Glenmark Pharmaceuticals Limited will be required to review and approve all versions of the SAP before database lock.

- 1. Study Objectives
     1. Primary Objective

• The primary objective of this study is to evaluate the efficacy of Nitric Oxide Nasal Spray combined with standard supportive care compared with standard supportive care alone in adult subjects with COVID-19 not requiring hospitalization.

- - 1. Secondary Objective

• The secondary objective is to evaluate the safety and tolerability of Nitric Oxide Nasal Spray combined with standard supportive care compared with standard supportive care alone in adult subjects with COVID-19 not requiring hospitalization.

- 1. Study Endpoints
     1. Primary Endpoints
- Change from baseline in log viral load through Day 8
  - 1. Secondary Endpoints
- Proportion of subjects with negative conversion of SARS-CoV 2 RT PCR on Day 2, 3, 4, and 8.
- Determine effect of NO nasal spray on clinical recovery [Time Frame: 18 days]. Determine the time to clinical recovery in participants with COVID-19 by measuring the proportion of patients from enrollment to resolution of baseline flu-like symptoms.
- Proportion of subjects requiring hospitalization for the treatment of COVID 19 [Tim frame: 18 days]
- Proportion of patients achieving a 2 point worsening in WHO Progression scale on Day 2, 3, 4, 8, and 19
- Time from randomization to first time use of high flow supplemental oxygen/non-invasive ventilation/mechanical ventilation/ extracorporeal membrane oxygenation.
- Change from baseline in COVID-19 related symptom score of participants with COVID- 19 at Day 2, 3, 4, 8, and 19
- Change from baseline in log Viral load at Day 2, 3, 4, and 8 [Time Frame: 7 days]
- Safety and tolerability of 6 day administration of NO nasal spray treatment over 18 days [Time Frame: 18 days]. Measure the tolerability of the NO nasal spray treatment as determined by number of adverse events, pain, discomfort or discontinuations of treatment.

1. study design
   1. Summary of Study Design

This is a randomized, double-blind, multi-center, parallel arm, clinical study evaluating the efficacy and safety of Nitric Oxide Nasal Spray with standard supportive care vs standard supportive care alone in patients with COVID-19 not requiring hospitalization.

306 eligible patients will be randomized in a 1:1 ratio into 2 groups: one group will receive Nitric Oxide Nasal Spray (NONS) along with standard supportive care and the control group will receive standard supportive care along with placebo of NONS. Standard supportive care will be provided in accordance with latest guidelines issued by Ministry of Health and family welfare; Government of India. Treatment duration is 7 days (up to Day 8 visit) and the total study duration will be maximum for 18 days from randomization (up to Day 19 visit). Viral load will be estimated on Day 1 (baseline), Day 2 (post-24 hours of dosing), Day 3 (post 48 hours of dosing), Day 8. Patients may be detained in a facility to facilitate protocol assessments for first 7 days or may be treated at home based on investigator discretion. Subjects who are treated at home will visit the study center on Day 1, 4, and 8 for study assessments and Day 2 visit (and swab sample collection for visit 2) will be conducted at home. If subjects are not able to visit the study center on any of the days, telephonic or video-conference and home based assessments can be conducted.

- 1. Definition of Study Drugs

Investigational Product:

Nitric oxide nasal spray, administered as a nasal spray, at a dose of two sprays each nostril, six times a day (along with standard supportive care). Study medication should be administered upon awakening (Dose 1), then administer additional doses (Dose 2 to 6) approximately every 2-3 hours while awake. Doses should be separated by at least 1.5 hours. Preferably, administer the last dose of the day (Dose 6) at bedtime.

Investigational Drug Products will be Manufacture and Supplied by SaNOtize Research and Development Corp. 25th Floor, 700 West Georgia Street, Vancouver, BC, Canada [Manufactured at Nextar Chempahrm Solutions Ltd. 13B Einstein St. Weizmann Science Park, Ness Ziona 74140 Israel.]

Comparator:

Placebo of Nitric oxide nasal spray, administered as a nasal spray, at a dose of two sprays each nostril, six times a day along with Standard supportive care.

- 1. Sample Size Considerations
     1. Sample Size Justifications

Based on the data of phase 2 study of Nitric Oxide Nasal Spray in the United Kingdom (Clinical Study Report IRAS ID 287727 NONS COVID Study), assuming a treatment effect of 5.0 log viral load in the primary endpoint, with a standard deviation of 10.0. One hundred seventy two (172) evaluable subjects (86 subjects per arm) will provide a power of 90% with two sided significance level of 5%. For the secondary endpoint (RT-PCR conversion), assuming RT-PCR conversion of 40% in the Placebo group by Day 8, 260 subjects (130 subjects in each arm) will provide a power of 90% at two-sided significance level of 5 %, to detect a treatment difference of 20% in proportion of subjects achieving RT-PCR conversion.

Assuming a dropout rate of 15 %, total 306 subjects (approx. 153 subjects per arm) will be randomized.

- - 1. Sample Size Re-estimation

Sample size may be adjusted based on the interim analysis results.

- 1. Randomization

After the SAS code and dummy randomization list were reviewed, validated and approved by senior biostatistician, an independent statistical programmer at Glenmark prepared the randomization list for the study using SAS version 9.4.

- 1. Clinical Assessments

All clinical assessments are listed in the following table. Safety assessments will consist of monitoring and recording all adverse events (AEs) and serious adverse events (SAEs), clinical laboratory measurements, vital signs, electrocardiograms, and physical examinations.

Schedule of Procedures and Assessments

| Study Period | Treatment Period | | | | | | | Early Withdrawal Visita | Post-Treatment Follow-Up (Telephonic or clinic visit^f^) |
| --- | --- | --- | --- | --- | --- | --- | --- | --- | --- |
|  | Screening & Randomization | |  | | | | End of Treatment  **(EOT)** |  |  |
| Visit | 1 | | 2 | | Lab Visit – Day 3 | 3 | 4 |  | 5 |
| Time Point (days) | 1 | | 2 | |  | 4 | 8+1 |  | 19±2 (or anytime between visit 4 and day 19) |
| Written informed consent | X | |  | |  |  |  |  |  |
| Demographics | X | |  | |  |  |  |  |  |
| Medical and surgical history | X | |  | |  |  |  |  |  |
| Review COVID-19 Symptoms and clinical status | X | | X | |  | X | X | X | X |
| Prior and concomitant medications | X | | X | |  | X | X | X | X |
| Physical examination | X | |  | |  |  | X | X |  |
| Height and Weight | X | |  | |  |  |  |  |  |
| Vital signs, including SpO_2_^b^ | X | | X | |  | X | X | X |  |
| 12-lead ECG^c^ | X | |  | |  |  | X | X |  |
| Hematology, serum chemistry, urinalysis | X | |  | |  |  | X | X |  |
| C-Reactive Protein | X | |  | |  |  |  |  |  |
| Chest X-ray^h^ | X | | X | |  | X |  |  |  |
| Methemoglobin^d^ | X | | X | |  | X | X | X |  |
| **Study Period** |  | **Treatment Period** | | | | | | **Early Withdrawal Visita** | **Post-Treatment Follow-Up (Telephonic or clinic visit^f^)** |
|  | **Screening & Randomization** | |  |  | | | End of Treatment  **(EOT)** |  |  |
| **Visit** | **1** | | **2** | |  | **3** | **4** |  | **5** |
| **Time Point (days)** | **1** | | **2** | |  | **4** | **8+1** |  | **19±2** |
| Quantitative RT PCR (nasopharyngeal swab) | X | | X | | X | X | X | X |  |
| Qualitative RT PCR (nasopharyngeal swab) | X | | X | | X | X | X | X | X^g^ |
| Review inclusion/exclusion criteria | X | |  | |  |  |  |  |  |
| Randomization | X | |  | |  |  |  |  |  |
| Pregnancy test^e^ | X | |  | |  |  | X | X |  |
| Assessment of AEs/SAEs | X | | X | |  | X | X | X | X |
| WHO Progression Scale Review | X | | X | |  | X | X | X | X |
| Spray Bottle Use Perception Questionnaire | X | | X | |  |  | X | X |  |
| Subject Diary dispensing | X | |  | |  |  |  |  |  |
| Subject Diary Review |  | | X | |  | X | X | X |  |
| Investigational product/study drug dispensing | X | |  | |  |  |  |  |  |
| Drug and Diary accountability |  | | X | |  | X | X | X |  |

AE = adverse event; ECG = electrocardiogram; SAE = serious adverse event.

^a^ Early withdrawal visit to be performed if applicable. If early withdrawal visit is performed on the day of scheduled visit, then both scheduled visit and early withdrawal visit assessments will be performed on the same day. Follow-up visit should be performed 11±2 days after early withdrawal visit, if there is subject’s consent.

^b^ If visits 2 (day 2) or 3 (day 4) are performed as home-based visits, blood pressure measurements and physical examination are not required

^c^ For 12-Lead ECG, if any clinically significant abnormality is detected, an additional triplicate ECG will be recorded.

^d^Methemoglobin assessment will be performed in only a subset of patients. In this subset, methemoglobin will be measured on day 1 (before randomization and 5 minutes after first dose of study medication), and days 2, 4 and 8

^e^ Female subjects only. Urine and Serum pregnancy test will be performed at Screening and urine pregnancy test will be performed at rest of the visits.

^f^ Visit 5 will only be conducted for patients whose baseline COVID-19 symptoms are present at visit 4 and/or whose RT-PCR is positive at visit 4. Visit 5 can be conducted any time between visit 4 and day 19, based on subject’s status change to symptom free and RT-PCR negative. If there is no change in such status, visit 5 will be conducted on day 19 ± 2 days. If clinic visit is performed on Day 19, any additional assessments made can be recorded on unscheduled visit eCRF page, at the discretion of the investigator

^g^ Nasopharyngeal swab will be collected for qualitative RT-PCR at visit 5 only if RT-PCR is positive on Day 8 visit 4.

^h^Only chest x-ray will be conducted at screening visit and visit 2. At visit 3 (day 4), based on investigator discretion, chest x-ray or CT scan can be performed.

1. Planned Analyses
   1. Interim Analysis

Interim analysis will be performed when ~50 % of the subjects complete the study according to group sequential design and sample size will be re-assessed.

Based on the pre specified analysis and as per Subject Expert Committee (SEC) recommendation dated 29/12/2021, to perform another analysis of current recruited subjects to include high risk population.

The interim results of the primary endpoint will be used to estimate the predictive probability of success (POS) at the end of the study by using Bayesian method with non-informative prior.

Here are the rules based on POS:

- If the POS of the primary endpoint (the Average of viral load) > 0.7, the study will continue, the study will continue.
- Otherwise the sample size may be revised and further decision will be taken based on the guidance from India regulator.

Because early stop for success will not be used, the alpha error will be controlled without alpha cost at the interim analysis.

- 1. Final Analyses

At the end of the study, a final analysis of data from all subjects will be planned.

Planned post-hoc analysis: A post-hoc analysis may be conducted in the subgroup of age.

1. General Considerations for Data Analyses and Handling
   1. General Summary Table and Individual Subject Data Listing Considerations

Summary tables and listings (e.g., post text tables and individual subject data listings are prepared according to ICH Guideline E3) include a “footer” providing explanatory notes that indicate as a minimum:

1. Date of data extraction.
2. Date of output generation.
3. SAS program name, including the path that generates the output.
4. The name of programmer and Date of outputs.
5. Any other output specific details that require further elaboration.

Post text tables also include reference(s) to the subject data listing(s) that supports the summary data. The data extraction date links the output to the archived database that is locked to ensure the replication of the results.

In general, post text tables will be organized with respect to treatment group and a column will be included to summarize all treated subjects. For comparative studies, the order of drug presentation will be investigational drug first followed by placebo (if it exists) and all other active comparative agents. A total column can appear as the last column. When appropriate, tables will display sub-group differences by treatment group. Row entries in post text tables are made only if data exists for at least one subject (e.g., a row with all zeros will not appear). The only exception to this rule applies to tables that summarize the study termination status of subjects (e.g., reasons for not completing the study). In this case, zeros will appear for study termination reasons that no subject satisfied. The summary tables clearly indicate the number of subjects to which the data apply and unknown or not performed are distinguished from missing data.

Summary tables for medications and medical conditions are coded according to standard dictionaries (e.g., WHO Drug standard dictionary, Sep 2018 version Sep 2018 version of Sep 2018 version of ). Adverse event preferred terms and body/organ systems are coded using dictionaries, such as, WHO ART, COSTART, and MedDRA. The MedDRA dictionary can be used, as well, in the coding of signs and symptoms, medical history, physical examination abnormalities, and clinical diagnoses to map to MH, AE, and Concomitant Procedures module MedDRA is mapped to MH, AE, and Concomitant Procedures module mapped to MH, AE, and Concomitant Procedures module to MH, AE, and Concomitant Procedures module.

Supportive individual Subject Data Listings, as a minimum, are sorted and presented by treatment group (group) and investigational site (center). Listings also include subject number, visit number, visit date, and days relative to the initiation of double-blind treatment.

Other subject data listings that do not support a specific summary table are included to provide an enumeration of the investigator’s general comments. This listing is also organized by treatment group and by investigator. Sorting is also performed with respect to subject, reference visit number and date, visit relative day, date of the comment, and the text of the comment.

No imputations are imposed for missing clinical data. Specific algorithms, however, can be discussed for imputing missing or partially missing dates, if deemed appropriate, under specific data topics. Imputed or derived data should be flagged in the individual subject data listings. Imputed data are not incorporated into any raw or primary datasets. These data are retained in derived analysis datasets.

- 1. General Post Text Summary Table and Individual Subject Data Listing Format Considerations

The default convention is to number tables and listings using a decimal system to reflect main levels of unique tables and listings and sub-levels of replicate tables and listings with two digits per level (e.g., Table XX.YY.ZZ. …).

1. The first level number should be consistent with the corresponding CSR appendix in which the tables or listings will appear. For example, the post text tables usually occupy Appendix 14 and the individual subject data listings are put in Appendix 16. All post text tables should have a main number level 14 and listings 16. The subject accounting and disposition table is usually first in the first section of the report and should be numbered Table 14.1. The supportive subject data listing would be Listing 16.1.
2. Subject accounting and final disposition should appear as the second level number (Table 14.1 series). Baseline and demographic profile occupies the next sub-level (Table 14.1.2 series). Efficacy should come next (14.2 series) followed by safety (table 14.3 series). Reasons for subjects’ being excluded from efficacy and protocol violation summary tables should appear as the last level (Table 14.4 series). Similar conventions should be applied to the subject data listings.
3. The title should be complete, accurate, and concise. The last line of the title should provide the analysis group being summarized (e.g., Intent-to-Treat Subjects or Per-Protocol Efficacy Subjects). If possible, the units of measurement for data contained in the table can appear in parentheses to conserve space in the body of the table. For example, the summary of vital signs title could read “Summary of Sitting and Supine Blood Pressure (mmHg) and Heart Rate (bpm).” Whether in the title or body of a table or listing, units must always be specified for all appropriate data.
4. If possible, variables being summarized and statistics reported should appear in the left most column of a table. The next columns for treatment groups should report the data from left to right for the investigational drug, placebo, comparative agents, and (optional) all treated subjects, respectively.

In general, the listings should be sorted and presented by treatment assignment, investigational site, and subject number. Treatment assignment and site can appear in the banner of the listing. From left to right, the subject number, visit number, visit date, and relative day should appear. All tables and listings must have explanatory notes that give, as a minimum, data extraction date, output generation date, programmer name, complete program name and path where it is stored, CRF pages from which the data were obtained, and supportive listings or tables supported, as appropriate. The definition of all derived variables and decodes for coded data must appear in the notes. Due to space limitations, tables and listings may require a page of notes as a one-time preface to the output.

Tables, Listings and Figures are numbered following the ICH structure. Table headers, variables names and footnotes will be modified as needed following data analyses. Additional Tables, Figures and Listings will be generated, as needed, following the data analysis (post-hoc).

- 1. Data Management

Study data will be entered into CRFs. Before data analysis, programmed edit checks will be run against the database to check for discrepancies and reasonableness of the data. All issues resulting from the computer-generated checks will be resolved.

- 1. Data Presentation Conventions

Continuous variables (e.g. age) are summarized using descriptive statistics (the number of subjects with available data, the mean, standard deviation (SD), median and minimum and maximum). Categorical variables (e.g. race) are summarized using counts and percentages. Percentages are calculated using the total subjects per treatment group.

The following conventions are applied to all data presentations and summaries.

- For continuous variables, all mean and median values are formatted to one more decimal place than the measured value. Standard deviation values are formatted to two more decimal places than the measured value. Minimum and maximum values are presented with the same number of decimal places as the measured value.
- For categorical variables, the number and percentage of responses are presented in the form XX (XX.X%) where the percentage is in the parentheses.
- Date variables are formatted as DDMMMYYYY for presentation. Time is formatted in military time as HH:MM for presentation.
- Wherever possible, data will be decimal aligned.
- P-values, if applicable, will be presented to 4 decimal places. If the p-value is less than 0.0001 then it will be presented as <0.0001. If the rounded result is a value of 1.000, it will be displayed as >0.9999.
- Unless otherwise stated, any statistical tests performed will use 2-sided tests at the 5% significance level.

The table and listing shells and table of contents as part of this SAP provide the expected layout and titles of the tables, listings and figures. Any changes to format, layout, titles, numbering, or any other minor deviation will not necessitate a revision to the SAP nor will it be considered a deviation from planned analyses. Only true differences in the analysis methods or data handling will necessitate such documentation. The appropriate listings supporting the tables will be included and are not specified in the individual sections throughout the document.

- 1. Analysis Populations

Analysis of the primary endpoint will be conducted using the modified ITT (mITT) analysis set. In addition, a supportive analysis will be performed for the primary efficacy endpoint using the Per Protocol Set (PPS).

**mITT analysis set**

The mITT analysis set will include all randomised subjects who received at least one dose of study medication, who have a non-missing baseline measurement and at least one post-baseline efficacy measurement for primary efficacy variable. Viral load values below limit of quantification at baseline visit will be considered as missing values. Subjects with negative RT-PCR at baseline will not be included in the mITT analysis set.

**Per protocol analysis set**

The per protocol analysis set (PPS) will include all subjects who are randomized, received at least one dose of study medication, completed the study and do not have any major protocol deviations. Major protocol deviations will be discussed and decided at the blinded data review meeting (BDRM) meeting before database lock.

**Safety analysis set**

The Safety analysis set (SAS) will include all subjects who are randomized and received at least one dose of study medication. All safety endpoints will use the safety analysis set unless otherwise specified.

- 1. Baseline Definitions

**Baseline value:** The Baseline value will be defined as pre-dose measurement at day 1 or the last non-missing value prior to first dose date.

**High risk population:** High-risk population is defined as subjects who meet one or more of these criteria:

• co-morbidity (diabetes, cardiovascular, PTs to be decided)

• age ≥45 years

• non-vaccinated

- 1. Derived and Transformed Data
     1. Baseline Age

Age at baseline will be calculated as follows:

- Baseline Age (years) = FLOOR((date of informed consent – date of birth day +1)/365.25).
- Age (year) = FLOOR((date of informed consent – date of birth)/365.25*12)

where FLOOR( ) function returns the integer part of the result.

- - 1. Study Day

Study day will be computed by subtracting the date of the first dose of study drug from the date of last dose and adding one. Thus, the first day of dosing is defined as Day 1.

- Treatment days = last dose date – first dose date + 1.
  - 1. Change from Baseline

Change from baseline will be calculated by subtracting the baseline measurement from the post baseline measurement.

- Change from baseline = post baseline measurement – baseline measurement.

Percent change from baseline is calculated as (change from baseline/baseline result * 100). If either the baseline or the post-baseline result is missing, the change from baseline and/or percentage change from baseline is set to missing as well.

6.7.4. Handling laboratory data out of the range of quantification

If any laboratory value (except baseline viral load) falls above or below the upper or lower level of quantification, the value of the upper or lower level of quantification will be taken (e.g. <0.2 will become 0.2) for summaries but left as recorded in the listing. For viral load at baseline, values below limit of quantification will be considered as missing values. For viral load at post-baseline visits, for values below limit of quantification (LoQ), LoQ value will be used.

- 1. Handling of Missing Data
     1. Missing Efficacy Endpoints

For the primary endpoint, only observed values of viral loading will be used to calculate the Average of viral load or normalized AUC and missing values will not be imputed. Outliers may be investigated by the Grubbs test.

Since MMRM which has built-in mechanism for missing data, no imputation will be done on the missing data for all the continuous endpoints with multiple time-points efficacy and safety parameters when MMRM is applied. The analysis will be done on the available non-missing data.

Imputation of missing data will be only performed when ANCOVA is used. Last observation carried forward (LOCF) will be performed for this study. If either the baseline or the post-baseline result is missing, the change from baseline is set to missing

For the time-to-event endpoints, censoring is used to deal with missing data in which time to event is not observed for reasons such as termination of study before all recruited subjects have shown the event of interest or the subject has left the study prior to experiencing an event. Subjects who terminated the study without documented event are censored at day 18. Subjects who die without documented event are censored at day 18 or the date of death whichever is later.

- - 1. Missing Start and Stop Dates for Prior and Concomitant Medication

For the purpose of inclusion in prior and/or concomitant medication tables, incomplete medication start and stop dates will be imputed as follows:

• If year and month are present and day is missing, then set day to first day of month for start date, and set day to last day of month for end date

• If year and day are present and month is missing, then set month to January for start date, and set month to December for end date

• If year is present and month and day are missing, then set month and day to January 1 for start date, and set month and day to December 31 for end date

• Completely missing date will not be imputed

The partial dates will be provided as imputed dates in the subject data listings.

- - 1. Missing Start and Stop Dates for Adverse Events

Due diligence will be done to obtain accurate AE information. If all planned methods to obtain accurate AE information have failed, missing and partial AE onset and end dates will be imputed. Imputed dates will be flagged in the individual supportive subject listings. Unless otherwise specified, the following conventions will be used:

Missing and Partial AE onset dates:

• If onset date is completely missing, then onset date is set to date of first dose

• If onset year is present and month and/or day are missing:

 If onset year = year of first dose, then set onset date to date of first dose

 If onset year < year of first dose, then set onset month and day to December 31st.

 If onset year > year of first dose, then set onset month and day to January 1st

• If onset month and year are present and day is missing:

If onset year = year of first dose and

 onset month = month of first dose then set onset date to date of first dose

 onset month < month of first dose then set onset date to last day of month

 onset month > month of first dose then set onset date to 1st day of month

If onset year < year of first dose then set onset date to last day of month

If onset year > year of first dose then set onset date to 1st day of month

• For all other cases, set onset date to date of first dose

Missing and Partial AE end dates:

• If end date is completely missing, end date is not imputed and the AE is flagged as “ongoing”

• If year is present and month and/or day are missing

 If year = year of last dose, then set end date to the date of last dose

 If year < year of last dose, then set end month and day to December 31st

 If year > year of last dose, then set end month and day to January 1st

• If month and year are present and day is missing:

If year = year of last dose and

 month = month of last dose then set day to day of last dose

 month < month of last dose then set day to last day of month

 month > month of last dose then set day to 1st day of month

If year < year of last dose, then set end date to last day of the month

If year > year of last dose, then set end date to 1st day of month

• For all other cases, set end date to date of last dose

1. Study Population
   1. Subjects Disposition

A detailed description of the subject disposition will be provided by treatment arm. It will include the following:

• A definition of subjects enrolled (per arm) in the trial

• A summary of data on subject completion and discontinuation

- 1. Screen Failures

Subjects who give informed written consent but are not dispensed study medication are considered screen failures. In general, no attempt will be made to further characterize the reason for screen failures in summary tables due to the paucity of information usually gathered. Number and percentage of subjects who were screened for the study and screen failure will be summarized.

- 1. Protocol Deviations

Deviations from the protocol including violations of inclusion/exclusion criteria will be assessed as ‘minor’ or ‘major’. Major protocol deviations are defined as those deviations from the Study Protocol likely to have an impact on the perceived efficacy and/or safety of study treatments. If required, the impact of major protocol deviations on the efficacy and/or safety results will be investigated by assessing the robustness of the study results and conclusions to the choice of analysis population, both including and excluding data potentially affected by major protocol deviations. Major protocol deviations and any action to be taken regarding the exclusion of subjects are defined in the Study Protocol deviation specifications. The final determination of major protocol deviations and the exclusion of subjects from any of the analysis populations will be made prior to un-blinding database lock.

A by-subject listing of major protocol deviations will be provided. In addition, the number and percentage of subjects who completed study treatment and discontinued study treatment before end of study Day will be summarized, along with the reason.

- 1. Demographic and Baseline Characteristics

The demographic and baseline characteristics will be summarized on the safety populations.

Age (calculated from the date of birth), sex, disease severity, disease signs and symptoms at screen will be summarized with descriptive statistics for each treatment arm.

- 1. Listing of Subject Inclusion and Exclusion Criteria

Subjects meeting any of the inclusion and exclusion criteria will be listed along with the reason. Any subject withdrawal during the study, along with the reason for withdrawal, will be documented in listings.

- 1. Prior and Concomitant Medications

Prior and Concomitant medications on study subjects collected and the conditions mentioned in study protocol will be summarized and provide listings.

- 1. Baseline Laboratory Data

Baseline for laboratory results is defined as the last assessment just prior to the first dose of study medication (Day 1) regardless of whether it was scheduled, retest, or unscheduled. All treated subjects with a baseline laboratory determination will be included. Summary tables are present for each category of data separately. Routine clinical laboratory data usually include hematology, serum chemistry, and urinalysis. Quantitative laboratory test result summaries will include N, mean, s.d., median, and range. Qualitative tests will be categorized accordingly. The set of laboratory parameters included in each table will correspond to those requested in the study protocol.

Subject data listings will include the laboratory test, test units, laboratory test result, and the laboratory standard normal ranges, if available.

- 1. Baseline Primary and Secondary Efficacy Evaluations

Not applicable.

1. Method of Analysis

All the efficacy and safety parameters will be analyzed till maximum of 18 days.

AEs and SAEs which are still on going at 18 days will be monitored and reported in the study analysis at 18 days. Which means, all AEs occurred during 18 days and completed after 18 days, will be included in the analysis for 18 days, as start date of AE occurred on/before 18 days.

All efficacy endpoint will be analyzed using ITT Population.

The statistical analysis will be performed using the statistical software SAS 9.4 version or latest available (SAS Institute Inc., Cary, North Carolina).

Quantitative data will be summarized using number of subjects, mean, standard deviation, median, minimum and maximum.

Qualitative data will be summarized using frequency and percentage.

Time to event data will be summarized using no of patients, no of events and percentage.

Categorical variables will be compared between the treatment arms using chi-square/Fisher’s exact test as appropriate.

For continuous variables with multiple time-points, mean change from baseline data will be compared between the treatment arms using MMRM. The Mixed-effect Model Repeated Measure (MMRM) model will be fitted when some of the response values are missing by assuming that they are missing at random (MAR). This will be done computationally using Restricted Maximum-Likelihood (REML) with a common unstructured covariance matrix among visits for each treatment group. If the model fails to converge, alternative structures (VC, AR1, TOEP1, and CS) or ANCOVA will be investigated. The adjusted means for each treatment and the estimated treatment differences for the treatment comparisons will be presented together with 95% confidence intervals (CIs).

Time to event variables will be analyzed using the Kaplan-Meier method to generate the K-M plot and to calculate the median time to event and its 95% confidence interval (CI) for each arm. log-rank test will be used to calculate the p-value to compare the two arms. Cox regression model will be used to calculate the hazard ration (HR) of the test vs control and its 95% CI.

1. Efficacy

Subjects with negative RT-PCR at baseline will be excluded from the analysis.

- 1. Analysis of the Primary Efficacy Endpoint
     1. Primary Efficacy Endpoints Analysis

**Change from baseline in log viral load through Day 8**

According to FDA guideline for easy interpretation, it will be measured using the average of viral load (log_10_ copies per milliliter) between day 1 to each visit day up to day 8.

As a sensitivity analysis, it will also be measured using the AUC of viral load (log_10_ copies per milliliter) between day 1 to each visit day up to day 8 normalized by day (day 2 - day 3, day 2 - day 4, day 2 - day 8). The time-weighted average change from baseline (day 1) to each visit will be calculated for each subject as the area under the concentration–time curve, with the use of the linear trapezoidal rule for change from baseline divided by the time interval of the observation period

The comparison of Nitric Oxide versus Placebo in the Average of viral load or AUC will be analyzed using Mixed-effect Model Repeated Measure (MMRM) model.

The null hypothesis is that there is no difference between Nitric Oxide and Placebo. The alternative hypothesis is that Nitric Oxide is not equal to Placebo in the average Change from baseline in log viral load through Day 8.

The MMRM model will include data from all visits until day 8 and the following covariates: treatment, visit, baseline value (log_10_ copies per milliliter), risk factor (high risk yes/no), center, and treatment by visit interaction; unstructured covariance matrix will be used, thus allowing adjustment for correlations between the time points within subjects. The differences between the Nitric Oxide (T|), and Placebo (P) will be compared based on the above MMRM model, and p-values and 95% Confidence intervals (CI) of T-P will be provided. If the upper bound of 95% CI is lower than 0, i.e. the p-value (two-sided) is less than 0.05, claiming the treatment arm is superior to the placebo arm.

Primary analysis will be conducted in the mITT population and sensitivity analysis will be conducted in the PP population. ANCOVA with LOCF analysis will be done in mITT as the sensitivity analysis as well. As RT-PCR conversion as early as day 2 suggests late COVID-19 infection at the time of screening and not appropriate to study treatment difference, separate analysis will be conducted including and excluding subjects who become RT-PCR negative on Day 2.

Sub-group analysis for high risk population and based on vaccination status sub-groups (yes/no), co-morbidity sub-group (yes/no) and baseline viral load sub-groups (>3, >4 and >5) will also be conducted.

Outliers may be investigated by the Grubbs test in sensitivity analysis.

- - 1. Analyses of the related secondary endpoints
- **Proportion of subjects with negative conversion of SARS-CoV 2 RT PCR on Day 2, 3, 4 or 8**

It will be analyzed using the chi-square test or Fisher’s exact test. Rate will only account the number of events which happened up to day 2/4/8 and will be based on the qualitative RT-PCR positive/negative results. The rate differences between the Nitric Oxide (T|), and Placebo (P) will be compared, and p-values and 95% Confidence intervals (CI) of T-P will be provided. As RT-PCR conversion as early as day 2 suggests late COVID-19 infection at the time of screening and not appropriate to study treatment difference, separate analysis will be conducted including and excluding subjects who become RT-PCR negative on Day 2.

- **Change from baseline in log Viral load at Day 2, 3, 4, and 8 [Time Frame: 7 days]**

It will be analyzed using a Mixed Model Repeated Measure (MMRM) method. The MMRM model will include data from all visits until day 19 and the following covariates: treatment, visit, baseline value, risk factor (high risk yes/no) center, and treatment by visit interaction; unstructured covariance matrix will be used, thus allowing adjustment for correlations between the time points within subjects. As RT-PCR conversion as early as day 2 suggests late COVID-19 infection at the time of screening and not appropriate to study treatment difference, separate analysis will be conducted including and excluding subjects who become RT-PCR negative on Day 2.

- - 1. Other additional Supportive Analyses of the Primary Efficacy Endpoints

The time to event (i.e. negative SARS-CoV2 RT-PCR result) related to the primary endpoint will be analyzed using the Kaplan-Meier method and log-rank test. Potential influencing factors of viral clearance will be analyzed by Cox regression model. In the Cox model, the time to event (i.e. negative SARS-CoV2 RT-PCR result) will be set as the Time variable, censoring (0 = no, 1 = yes) will be set as the status, and the variables including age-group populations (≤45 years and >45 years), treatment, co-morbidity yes/no and high risk yes/no as independent variables. As RT-PCR conversion as early as day 2 suggests late COVID-19 infection at the time of screening and not appropriate to study treatment difference, separate analysis will be conducted including and excluding subjects who become RT-PCR negative on Day 2.

Patients who do not have documented negative SARS-CoV2 RT-PCR event at the time of the data cut-off for the primary efficacy analysis will be censored at the day 18. Patients who have died within 18 days who have no documented event, will be censored at day 18. Patients who have died after 18 days who have no documented event, will be censored at date of death. Additional analysis will be conducted using alternate censoring method.

The null hypothesis (H0) is that the survival distributions of the primary endpoint in the two treatment arms (denoted as S<Test-drug> or S<Control>) are the same. The alternative hypothesis (H1) is that the survival distribution in the treatment arm and the control arm are different:

H0: S<Test-drug> = S<Control> vs. H1: S<Test-drug> ≠ S<Control>

The log rank test will be used to compare the distributions between treatment arms. The Kaplan-Meier approach will be used to estimate the median time to event for each treatment arm and the corresponding two-sided 95% CI. The Cox proportional hazard model will be used to estimate the hazard ratio (HR) between the two treatment arms and its 95% confidence interval (CI).

If HR (T/P) is higher than 1 and the p-value is less than 0.05, one can claim the Test arm is superior to the Placebo arm.

Sub-group analysis will be done for high and low baseline Ct value (higher and lower than median Ct value), high risk yes/no subgroups, in subgroups including and excluding subjects with worsening of COVID-19, and different age-group populations (as post-hoc analysis) for the primary endpoints.
Additional analysis will be conducted:

- using definition of RT-PCR conversion based on viral load thresholds of 3 for the log_10_ copies per milliliter.
- Using definition of RT-PCR conversion as change in Ct value from below 30 to 30 and above, and change in Ct value from below 35 to 35.
  1. Analysis of other Secondary Endpoints

The following time event endpoints will be analyzed the same way as the primary endpoint using the Kaplan-Meier method, log-rank test and Cox analysis.

- Determine effect of NO nasal spray on clinical recovery [Time Frame: 18 days]. Determine the time to clinical recovery in participants with COVID-19 by measuring the proportion of patients from enrollment to resolution of baseline flu-like symptoms. Following definitions of clinical recovery will be used:
  - Resolution of all the COVID-19 symptoms present at baseline. End date of last symptom (score 0) will be the date of clinical recovery.
  - Resolution of common flu-like symptoms fever, cough and headache. End date of last of the three symptoms (score 0) will be the date of clinical recovery. Subjects with at least one of these 3 symptoms at baseline, will be included in this analysis.

Additionally, time to complete recovery, i.e. virological cure in terms of negative conversion of qualitative RT-PCR AND clinical cure based on the above 2 definitions, will be analyzed using the Kaplan-Meier method, log-rank test and Cox analysis; and proportion of patients with events at days 2, 4, 8 and 19 will be compared between groups using the chi-square test or Fisher’s exact test. In addition, separate analysis will be conducted based on cure date defined by the investigator.

Symptoms not present at baseline will not be considered in the analysis.

Analysis of time to event of individual symptom scores (fever, sore throat, bodyache, tiredness/fatigue) will be run.

- Time from randomization to first time use of high flow supplemental oxygen/non-invasive ventilation/mechanical ventilation/ extracorporeal membrane oxygenation.

The following endpoints will be analyzed using a Mixed Model Repeated Measure (MMRM) method. The MMRM model will include data from all visits until day 19 and the following covariates: treatment, visit, baseline value, risk factor (high risk yes/no), center, and treatment by visit interaction; unstructured covariance matrix will be used, thus allowing adjustment for correlations between the time points within subjects.

- Change from baseline in COVID-19 related symptom score of participants with COVID- 19 at Day 2, 3, 4, 8 and 19.
- Total symptom score will be calculated for each subject at each time point as per the US-FDA guidance and compared within and between groups. In addition, change from baseline in individual symptom score will be compared.

Symptom Scoring: Symptom score for each subject will be calculated for symptom at baseline and each visit day (days 2, 3, 4 and 8) based on subject diary data.

Each symptom will be scored individually using the following response options and scoring values:

- Items 1–10: None = 0; Mild = 1; Moderate = 2; and Severe = 3
- Items 11 and 12: Sense of smell/taste same as usual = 0; Sense of smell/taste less than usual = 1; No sense of smell/taste (lost) = 2
- Items 13 and 14: Not at all = 0; 1–2 times = 1; 3–4 times = 2; 5 or more times = 3

For symptoms that are recorded twice a day (items 1-10), worst (highest) score in the day for that symptom will be considered as the score for that symptom on that day.

Baseline score: Baseline score for each symptom will be the first symptom record in the subject diary, ie. on the day of randomization if morning record is present, morning score will be the baseline score and if morning score is not present on randomization day, evening record will be considered as baseline score. Total score will also be calculated for each day by addition of scores of each symptom.

Change from baseline: Change from baseline will be calculated for each symptom as well as total score on days 2, 3, 4 and 8.

The following binary endpoints will be analyzed using the chi-square test or Fisher’s exact test.

- Proportion of subjects requiring hospitalization for the treatment of COVID 19 [Tim frame: 18 days]
- Proportion of patients achieving a 2-point worsening in WHO Progression scale on Day 2, 3, 4, 8 and 19: Improvement in WHO Progression scale score will also be analyzed. Improvement will be defined as achieving score 0 or 2-point improvement in WHO Progression score. In the analysis of improvement subjects with baseline score of ≥2 will only be included.

Sub-group analysis will be done for high risk yes/no and different age-group populations (as post-hoc analysis) for the secondary efficacy endpoints.

Similarly, proportion of immediate contacts of the patients having symptoms of COVID-19 and proportion of immediate contacts of the patients with positive COVID-19 test results will be analyzed using the chi-square test or Fisher’s exact test.

- 1. Summary of Efficacy Analyses

Table below summarizes all of the endpoint analyses that will be done and the analysis methods and populations.

| **Endpoint** | **Analyses** | **Population (s)** |
| --- | --- | --- |
| Change from baseline in log viral load through Day 8 (Average/normalized AUC) | MMRM  ANCOVA | mITT and PP  mITT and PP |
| Proportion of subjects with negative conversion of SARS-CoV 2 RT PCR on Day 2, 3, 4, and 8. | Chi-square test or  Fisher’s exact test | mITT and PP  mITT excluding worsening and  PP excluding worsening |
| Time to negative conversion of SARS-CoV 2 RT PCR | Kaplan-Meier method, log-rank test and Cox regression | mITT |
| Time to clinical recovery in participants with COVID-19 | Kaplan-Meier method, log-rank test and Cox regression | mITT |
| Time from randomization to first time use of high flow supplemental oxygen/non-invasive ventilation/mechanical ventilation/ extracorporeal membrane oxygenation | Kaplan-Meier method, log-rank test and Cox regression | mITT |
| Proportion of subjects requiring hospitalization for the treatment of COVID 19 [Tim frame: 18 days] | Chi-square test or  Fisher’s exact test | mITT |
| Proportion of patients achieving a 2-point worsening in WHO Progression scale on Day 2, 3, 4, 8 and 19 | Chi-square test or  Fisher’s exact test | mITT |
| Change from baseline in COVID-19 related symptom score of participants with COVID- 19 at Day 2, 3, 4, 8 and 19 | MMRM | mITT |
| Change from baseline in log Viral load at Day 2, 3, 4, and 8 [Time Frame: 7 days] | MMRM | mITT |
| Compliance with the study medication | Summary Statistics | Safety analysis Set |
| Number and percentage of patients with treatment emergent adverse events (TEAE) | Summary Statistics | Safety analysis Set |

1. Safety Analysis
   1. Compliance

Percentage compliance will be calculated as:

Compliance (%)= ((Number of doses taken)/(Number of doses expected))x 100%

The denominator is the total number of doses expected to be consumed (6 times of number of days from randomization to the last day of treatment period). For subjects withdrawn early last day of treatment period will be the day of withdrawal. The numerator is the number of doses actually consumed. Total doses consumed will be recorded in the CRF.

Subjects with ≥ 80% of compliance will be considered as medication compliant.

Number of compliant subjects will be summarized in terms of frequency count (n) and Percentages (%). Summarization will be provided based on safety population.

- 1. Adverse Events

The investigator’s verbatim term of each AE will be mapped to system organ class and preferred term using the MedDRA Version 23.0 or latest available.

Adverse events will be summarized by system organ class and preferred term; a subject will only be counted once per system organ class and once per preferred term within a treatment group.

The TEAEs will be defined as an event not present prior to exposure to the treatment or, if present prior to exposure, an event, which worsens in either intensity or frequency following exposure to the treatment.

Subject counts and percentages with event will be presented for each treatment groups and totaled for all treatment groups for the following summaries:

1. All AEs

2. All treatment emergent adverse events (TEAEs)

3. TEAEs by relationship to study drug

4. TEAEs by Severity

6. Serious treatment emergent adverse events (TEAEs)

7. TEAEs leading to death

8. TEAEs leading to permanent discontinuation of study drug.

9. Summary of Frequency Categories of TEAEs for Favipiravir Favipiravir Nitric Oxide Nasal Spray.

Drug related AEs will be defined as AE with relationship to study drug as ‘Yes’.

If subject has more than one episode of an AE, subject will be counted only once in system organ class (SOC) and once for specific preferred term (PT).

Listings will be presented by subject for all adverse events.

- 1. Routine Laboratory Data

Clinical laboratory results at each time point and for change from baseline will be displayed using summary statistics.

The safety laboratory tests are as follows:

| Panel | Test to be performed |
| --- | --- |
| Hematology | hemoglobin  hematocrit (PCV)  red blood cell (RBC) count  mean corpuscular volume (MCV)  mean corpuscular hemoglobin concentration (MCHC)  white blood cell (WBC) count with differential (absolute number and percentages) [neutrophils, lymphocytes, monocytes, eosinophils, and basophiles]  platelet count |
| Serum Chemistry | Random blood glucose  alanine transaminase (ALT)  albumin  alkaline phosphatase (ALP)  aspartate transaminase (AST)  bilirubin (total, direct and indirect)  blood urea nitrogen (BUN)  calcium (Ca)  chloride (Cl)  cholesterol (total, LDL, HDL)  C‑reactive protein (only at screening)  creatinine  gamma‑glutamyl transferase (GGT)  lactate dehydrogenase (LDH, only at screening)  magnesium  phosphorus  potassium (K)  sodium (Na)  plasma total protein  triglycerides  uric acid |
| Urinalysis | color  appearance  pH  specific gravity  presence of blood, glucose, protein, bilirubin and urobilinogen  microscopy including WBC/high power field (HPF), RBC/HPF |
| Panel | Test to be performed |
| Pregnancy Testing | serum beta-human chorionic gonadotropin (serum β-hCG) (only at screening and for confirmation of pregnancy if urine pregnancy test is positive)  Urine Pregnancy Test (at site) |
| Other | Quantitative SARS CoV2 RT‑PCR (swab from both sides of nose) for efficacy assessment  Qualitative SARS CoV2 RT‑PCR (swab from both sides of nose) for efficacy assessment |

All clinical laboratory data will be presented in listings. All continuous laboratory parameters and the change in these parameters at each time points from baseline to end of treatment (Day of discharge) will be summarized using descriptive statistics (n, mean, SD, median and range (Min., Max) overall for the safety population.

Absolute and change from baseline values of laboratory test parameters (Hematology and Serum Biochemistry) will be summarized descriptively by study treatment.

Summary and listing will be prepared for 12 Lead ECG which provides the detailed information of 12 Lead ECG parameters (Heart Rate, PR Interval, RR Interval QRS Interval, QT Interval, QTcF Interval, QTcB interval) and their significance by subjects at screening visit and End of the treatment visit.

Listing will be prepared for Chest X-Ray/CT scan; result of Chest X-Ray/CR scan will provide the significance of abnormality by patients at screening visit.

Listing will be prepared for examinations for SARS CoV2 RT-PCR, SpO_2_ and Check for Oxygen support/NIV/MV/ECMO support.

- 1. Vital Signs

Vital signs such as Body Temperature, systolic and diastolic blood pressure, pulse rate, respiratory rate, Body Weight and change in these vital signs from baseline to each relevant time point will be summarized by treatment for the safety population using descriptive statistics (n, mean, SD, median and range (Min., Max)).

- 1. Physical Examination

Listing will be prepared for Physical Examination which provides the detailed information about the Body Systems (include head, neck, thyroid, eyes, ears, nose, throat, cardiovascular system, Respiratory System, Gastrointestinal System, skin, musculoskeletal, urinary system and genitalia) by subjects at each visit.

- 1. Unscheduled Assessment

Extra assessments (laboratory data, ECG or vital signs associated with non-protocol clinical visits or obtained in the course of investigating or managing adverse events) will be included in listings, but not summaries except for the baseline values. If more than one laboratory value is available for a given visit, latest valid assessment at a visit is used for summary and the repeat assessments will be used for analysis in case if all the assessments are on same date and there is non-missing time, and in case if dates are same and time is missing for some assessments then the average of the assessments will be used as the value for that time point.

- 1. Pharmacokinetic Measurements Analyses

Not applicable.

1. Appendix

Appendix : Table of Contents for Data Display Specifications in the separate mock shells file.
