# Supplementary material for: SARS-CoV-2 accelerated clearance using a novel nitric oxide nasal spray (NONS) treatment: A randomized trial
Source: Lancet Reg Health Southeast Asia. 2022 Jun 29;3:100036. doi: 10.1016/j.lansea.2022.100036 (PMC9239922; doi:10.1016/j.lansea.2022.100036)
Supplement: Supplementary file 4 [file mmc4.docx]

File 1: Supplementary Material Tables and Figures

File 2: Supplementary Material Study Protocol
